# Supplementary material for: Determinants of adolescents’ depression, anxiety, and somatic symptoms in Northwest Ethiopia: A non-recursive structural equation modeling
Source: PLoS One. 2024 Apr 10;19(4):e0281571. doi: 10.1371/journal.pone.0281571 (PMC11006201; doi:10.1371/journal.pone.0281571)

**S1 Fig : Hyphothetical measurment model for the determinants of depression, anxiety, and somatic symptoms among adolescents in Northwest, Ethiopia, 2022**


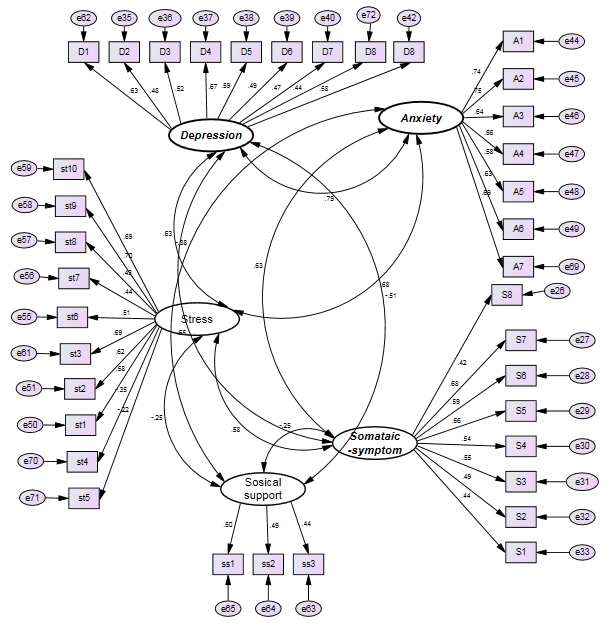

Supplement: S1 Fig — (DOCX) [file pone.0281571.s001.docx]
