# Supplementary material for: Determinants of adolescents’ depression, anxiety, and somatic symptoms in Northwest Ethiopia: A non-recursive structural equation modeling
Source: PLoS One. 2024 Apr 10;19(4):e0281571. doi: 10.1371/journal.pone.0281571 (PMC11006201; doi:10.1371/journal.pone.0281571)
Supplement: S1 Table — (DOCX) [file pone.0281571.s002.docx]

*S1 Table: Behavioral, academic, and relation-related factors of high and preparatory school adolescents in Northwest Ethiopia, 2022 (n=1379).*

| **Variables** | **Frequency** | **Percentage (%)** |
| --- | --- | --- |
| **Physical activity** | | |
| Inactive | 1155 | 83.76 |
| Active | 224 | 16.24 |
| **Ever use of alcohol** | | |
| No | 886 | 64.25 |
| Yes | 493 | 35.75 |
| **Extra school tutoring*(n=1378)** | | |
| No | 1011 | 73.3 |
| Yes | 367 | 26.7 |
| **Self-rated academic ability** | | |
| Very poor | 39 | 2.83 |
| Poor | 70 | 5.08 |
| Good | 656 | 47.57 |
| Very good | 390 | 28.28 |
| Excellent | 224 | 16.24 |
| **Father education *(n=1377)** | | |
| Can’t read and write | 266 | 19.29 |
| Grade 1- 8 | 488 | 35.43 |
| Grade 9-12 | 308 | 22.34 |
| Diploma and above | 315 | 22.84 |
| **Mother education* (n=1378)** | | |
| Can’t read and write | 429 | 31.12 |
| Grade 1- 8 | 436 | 31.63 |
| Grade 9-12 | 277 | 20.10 |
| Diploma and above | 236 | 1.13 |
| **Family academic pressure** | | |
| Not at all | 524 | 38 |
| Low | 375 | 27.19 |
| Medium | 144 | 10.44 |
| High | 336 | 24.37 |
| **Death of a loved one in the past 6 months** | | |
| No | 1058 | 76.72 |
| Yes | 321 | 23.28 |
| **Social support** |  |  |
| Poor | 564 | 40.9 |
| Medium | 559 | 40.54 |
| Good | 256 | 18.56 |
| **Current alcohol use(n=1377)*** | | |
| No | 368 | 26.73 |
| Yes | 1009 | 73.27 |
| Median  **Study time in hours(n=1379)** 3 | | IQR  ±3 |
| **Time spent for 2**  **extra school tutoring**  in hour(n=367) | | ±2 |

* Indicates variables with missing observations
