# Supplementary material for: Determinants of adolescents’ depression, anxiety, and somatic symptoms in Northwest Ethiopia: A non-recursive structural equation modeling
Source: PLoS One. 2024 Apr 10;19(4):e0281571. doi: 10.1371/journal.pone.0281571 (PMC11006201; doi:10.1371/journal.pone.0281571)
Supplement: S2 Table — (DOCX) [file pone.0281571.s003.docx]

***S2 Table: Summary of key variables used for the determinates of depression, anxiety, and somatic symptoms among adolescents in Northwest Ethiopia, 2022***

| Observed Exogenous | |
| --- | --- |
| Socio-demographic variables | age, sex, original residence, grade level, school type, father education, and mother education |
| Behavior related factors | physical activity, alcohol use |
| Academic related factors | extra school tutoring, number of study hours per day, self-rated academic ability, and family academic pressure |
| Relationships and related factors | Death of beloved one |
| Clinical factors | family history of mental illness, history of physical trauma, medically confirmed chronic illness |
| Exogenous latent variable | social support |
| Endogenous latent variable | Stress |
