# Supplementary material for: Determinants of adolescents’ depression, anxiety, and somatic symptoms in Northwest Ethiopia: A non-recursive structural equation modeling
Source: PLoS One. 2024 Apr 10;19(4):e0281571. doi: 10.1371/journal.pone.0281571 (PMC11006201; doi:10.1371/journal.pone.0281571)
Supplement: S3 Table — (DOCX) [file pone.0281571.s004.docx]

**S3 Table: Tool that was used for the determinants of depression, anxiety, and somat8ics symptoms among adolescents in Northwest Ethiopia, 2022.**

**Consent form**

I have read the information and recognize that my participation in this study is voluntary; I understand the objective, benefit and risk, procedure, and I am free to withdraw and not to answer any question that I don’t know without any consequences. I understand that the information from this study will be strictly confidential. I realize that I have the right to ask any question. I declare my voluntary consent to participate in this study with my signature below.

Are you voluntary to participate in this study?

Yes

No

If you say yes pleases put your signature here

Signature……………………………………….Date…………………..

Name of data collector------------------------ Sign-----------------Date of interview --------------

**Questionnaire**

The tool contains 9 sections with socio-demographic variables, behavioral related variables, academic related, relationship related variables, clinical factors, depression, anxiety, somatic symptom and stress indicators consecutively.

Students ID________

School type public private

Dear respected respondents, it is to kindly request you that, please encircle your choose for choose question and fill the blank spaces accordingly.

| Part I: Scio-demographic characteristics related variable | | | | | | | | | | | | | | | | | | | | | | | | | | | | | | | | | | | | | |
| --- | --- | --- | --- | --- | --- | --- | --- | --- | --- | --- | --- | --- | --- | --- | --- | --- | --- | --- | --- | --- | --- | --- | --- | --- | --- | --- | --- | --- | --- | --- | --- | --- | --- | --- | --- | --- | --- |
| Code | | Questions | Response | | | | | | | | | | | | | | | | | | | | | | | | | | | | | | | Remark | | | |
| 101 | | Age of the respondent | _______years old. | | | | | | | | | | | | | | | | | | | | | | | | | | | | | | |  | | | |
| 102 | | Sex of the respondent | 1. Male  2. Female | | | | | | | | | | | | | | | | | | | | | | | | | | | | | | |  | | | |
| 103 | | What is you grade level | 1. Grade 9 2. Grade 10 3. Grade 11 4. Grade 12 | | | | | | | | | | | | | | | | | | | | | | | | | | | | | | |  | | | |
| 104 | | What is your father educational status? | 1. Can’t read and write 2. 1-8 grade 3. 9-12 4. College and above | | | | | | | | | | | | | | | | | | | | | | | | | | | | | | |  | | | |
| 105 | | What is your mother educational status? | 1. Can’t read and write 2. 1-8 grade 3. 9-12 4. College and above | | | | | | | | | | | | | | | | | | | | | | | | | | | | | | |  | | | |
| 106 | | Residence of your family? | 1. Rural 2. Urban | | | | | | | | | | | | | | | | | | | | | | | | | | | | | | |  | | | |
| Part II: Behavioral related factors | | | | | | | | | | | | | | | | | | | | | | | | | | | | | | | | | |  | | | |
| 201 | | In the past one week how many days did you exercise for at least 60 minutes until we felt sweaty or shortness of breath | | | | | | 1. 0-1 days 2. 2-4 days 3. 5-7 days | | | | | | | | | | | | | | | | | | | | | | | | | |  | | | |
| 202 | | Have you a history of ever use of alcohol(like Tella, Beer, Tej, Katikala, Wine) | | | | | | | | | | | | | | 1. No 2. yes | | | | | | | | | | | | | | | | | | If no skip to Part III | | | |
| 203 | | If yes for Q 202, In the past 3 months do you have history of alcohol (Tella, katikala, beer, Woyn…..) drinking? | | | | | | | | | | | | | | 1. no 2. yes | | | | | | | | | | | | | | | | | |  | | | |
| Part III: Academics related factors | | | | | | | | | | | | | | | | | | | | | | | | | | | | | | | | | | | | | |
| 301 | | Is their additional tutorial than you regular school schedules? | | | | | | | | | | | | | | 1. No 2. Yes | | | | | | | | | | | | | | | | | | If no skip to 303 | | | |
| 302 | | If yes for Q205 how much times do you spend daily by extra school tutoring and its assignment. | | | | | | | | | | | | | | _____hour | | | | | | | | | | | | | | | | | |  | | | |
| 303 | | How much time do you study on average per day? | | | | | | | | | | | | | | _______hour | | | | | | | | | | | | | | | | | |  | | | |
| 304 | | How do you rate your perceived academic ability? | | | | | | | | | | | | | | 1. very poor 2. poor 3. good 4. very good 5. excellent | | | | | | | | | | | | | | | | | |  | | | |
| 305 | | How do you rate your family academic pressure? | | | | | | | | | | | | | | 1. no at all 2. a little 3. somewhat 4. medium 5. high | | | | | | | | | | | | | | | | | |  | | | |
| Part IV: Relationship related factors | | | | | | | | | | | | | | | | | | | | | | | | | | | | | | | | | |  | | | |
| 401 | | Is there a history of losing beloved one (mother, father, sister, brother, friend) due to death in the past 6 months? | | | | | | | | | | | | | | 1. no 2. yes | | | | | | | | | | | | | | | | | |  | | | |
| Perceived social support by using Oslo social support scale (OSS-3) | | | | | | | | | | | | | | | | | | | | | | | | | | | | | | | | | | | | | |
| 402 | | How many people are so close to you that you can count on them if you have great personal problems? | | | | | | | | | | | | | | 1.none  2. 1-2  3. 3-5  4. >5 | | | | | | | | | | | | | | | | | |  | | | |
| 403 | | How much interest and concern do people show in what you do? | | | | | | | | | | | | | | 1. none 2. little 3. uncertain 4. somewhat 5. a lot | | | | | | | | | | | | | | | | | |  | | | |
| 404 | | How easy is it to get practical help from neighbors if you should need it? | | | | | | | | | | | | | | 1.very difficult  2.difficult  3.possible  4.easy  5.very easy | | | | | | | | | | | | | | | | | |  | | | |
| Part V: Clinical factors | | | | | | | | | | | | | | | | | | | | | | | | | | | | | | | | | | | | | |
| 501 | | Is the any one in your family who had mental illness? | | | | | | | | | | | | | | 1.no  2. yes | | | | | | | | | | | | | | | | | | | | |  |
| 503 | | Have you a history of physical trauma? | | | | | | | | | | | | | | 1. no 2. yes | | | | | | | | | | | | | | | | | | | | |  |
| 505 | | Have we a history of physician diagnosed chronic illness?  **We can choose more than one answer.** | | | | | | | | | | | | | | 1. no 2. hypertension 3. diabetes mellitus 4. renal disease 5. other specify________ | | | | | | | | | | | | | | | | | | | | |  |
| Part VI: Patient Health Questionnaire (PHQ-9) to measure depression | | | | | | | | | | | | | | | | | | | | | | | | | | | | | | | | | | | | | |
| Over the last two weeks, how often have you been bothered  by any of the following problems? Click (✔) On the space provided in line with your answer. | | | | | | | Not at all | | | | | | Several days | | | | | | | | | More than half the days | | | | | | | Nearly every days | | | | |  | | | |
| 601 | | Feeling down, depressed, irritable, or hopeless | | | | |  | | | | | |  | | | | | | | | | | |  | | | | |  | | | | |  | | | |
| 602 | | Little interest or pleasure in doing things | | | | |  | | | | | |  | | | | | | | | | | |  | | | | |  | | | | |  | | | |
| 603 | | Trouble falling or staying asleep, or sleeping too much | | | | |  | | | | | |  | | | | | | | | | | |  | | | | |  | | | | |  | | | |
| 604 | | Poor appetite, weight loss, or overeating | | | | |  | | | | | |  | | | | | | | | | | |  | | | | |  | | | | |  | | | |
| 605 | | Feeling tired, or having little energy | | | | |  | | | | | |  | | | | | | | | | | |  | | | | |  | | | | |  | | | |
| 606 | | Feeling bad about yourself - or that you are a failure or have let yourself or your family down | | | | |  | | | | | |  | | | | | | | | | | |  | | | | |  | | | | |  | | | |
| 607 | | Trouble concentrating on things like school work,  Reading or watching TV | | | |  | | | | | | |  | | | | | | | | | | |  | | | | |  | | | | |  | | | |
| 608 | | Moving or speaking so slowly that other people could have noticed? Or the opposite - being so fidgety or restless that you have been moving around a lot more than usual | | | |  | | | | | | |  | | | | | | | | | | |  | | | | |  | | | | |  | | | |
| 609 | | Thoughts that you would be better off dead, or of hurting yourself in some way | | | | |  | | | | |  | | | | | | | | | | |  | | | | |  | | | | | |  | | | |
| Part VII : General anxiety scale(GAS-7) measure anxiety | | | | | | | | | | | | | | | | | | | | | | | | | | | | | | | | | | | | | |
| Over the last 2 weeks, how often have you been  Bothered by the following problems? Click (✔) On the space provided in line with your answer | | | | | Not at all | | | | | | Several days | | | | | | More than half the days | | | | | | | | | | | | | | Nearly every day | | |  | | | |
| 701 | | Feeling nervous, anxious or on edge | | |  | | | | | |  | | | | | |  | | | | | | | | | | | | |  | | | |  | | | |
| 702 | | Worrying too much about different things | | |  | | | | | |  | | | | | |  | | | | | | | | | | | | |  | | | |  | | | |
| 703 | | Not being able to stop or control worrying | | |  | | | | | |  | | | | | |  | | | | | | | | | | | | |  | | | |  | | | |
| 704 | | Trouble relaxing | | |  | | | | | |  | | | | | |  | | | | | | | | | | | | |  | | | |  | | | |
| 705 | | Being so restless that it is hard to sit still | | |  | | | | | |  | | | | | |  | | | | | | | | | | | | |  | | | |  | | | |
| 706 | | Becoming easily annoyed or irritable | | |  | | | | | |  | | | | | |  | | | | | | | | | | | | |  | | | |  | | | |
| 707 | | Feeling afraid as if something awful might happen | | |  | | | | | |  | | | | | |  | | | | | | | | | | | | |  | | | |  | | | |
| Part VIII. SSS-8 to measure somatic symptom | | | | | | | | | | | | | | | | | | | | | | | | | | | | | | | | | | | | | |
| During the past 7 days, how much have you been  Bothered by any of the following problems? Click (✔) On the space provided in line with your answer | | | | | | | | | | Not at all | | | | A little bit | | | | | | | somewhat | | | | Quite a bit | | | | | | | Very much | | |  | | |
| 801 | Stomach or bowel problems | | | | | | | | |  | | | | |  | | | | |  | | | | |  | | | | | | |  | | |  | | |
| 802 | Back pain | | | | | | | | |  | | | | |  | | | | |  | | | | |  | | | | | | |  | | |  | | |
| 8 | Pain in your arms, legs, or joints | | | | | | | | |  | | | | |  | | | | |  | | | | |  | | | | | | |  | | |  | | |
| 804 | Headaches | | | | | | | | |  | | | | |  | | | | |  | | | | |  | | | | | | |  | | |  | | |
| 805 | Dizziness | | | | | | | | |  | | | | |  | | | | |  | | | | |  | | | | | | |  | | |  | | |
| 806 | Chest pain or shortness of breath | | | | | | | | |  | | | | |  | | | | |  | | | | |  | | | | | | |  | | |  | | |
| 807 | Feeling tired or having low energy | | | | | | | | |  | | | | |  | | | | |  | | | | |  | | | | | | |  | | |  | | |
| 808 | Trouble sleeping | | | | | | | | |  | | | | |  | | | | |  | | | | |  | | | | | | |  | | |  | | |
| Section IX: perceived stress scale(PSS-10 ) to measure stress | | | | | | | | | | | | | | | | | | | | | | | | | | | | | | | | | | | | | |
| 901 | Click (✔) On the space provided in line with your answer  In the last month, how often have you been upset because of something that happened unexpectedly? | | | Never | | | | | Almost never | | | | | | | | | | Sometimes | | | | | | | | Fairly often | | | | | | Very often | | |  | |
|  |  |  |  |  | | | | |  | | | | | | | | |  | | | | | | | |  | | | | | | |  | | |  | |
| 902 | In the last month, how often have you felt that you were unable to control Important things in your life | | |  | | | | |  | | | | | | | | |  | | | | | | | |  | | | | | | |  | | |  | |
| 903 | In the last month, how often have you felt nervous and stressed? | | |  | | | | |  | | | | | | | | |  | | | | | | | |  | | | | | | |  | | |  | |
| 904 | In the last month, how often have you felt confident about your ability to handle your personal problems? | | |  | | | | |  | | | | | | | | |  | | | | | | | |  | | | | | | |  | | |  | |
| 905 | In the last month, how often have you felt that things were going your way? | | |  | | | | |  | | | | | | | | |  | | | | | | | |  | | | | | | |  | | |  | |
| 906 | In the last month, how often have you found that you could not cope with all the things that you had to do? | | |  | | | | |  | | | | | | | | |  | | | | | | | |  | | | | | | |  | | |  | |
| 907 | In the last month, how often have you been able to control irritations in your life? | | |  | | | | |  | | | | | | | | |  | | | | | | | |  | | | | | | |  | | |  | |
| 908 | In the last month, how often have you felt that you were on top of things? | | |  | | | | |  | | | | | | | | |  | | | | | | | |  | | | | | | |  | | |  | |
| 909 | In the last month, how often have you been angered because of things that happened that been outside of your control? | | |  | | | | |  | | | | | | | | |  | | | | | | | |  | | | | | | |  | | |  | |
| 910 | In the last month, how often have you felt difficulties were piling up so high that you could not overcome them? | | |  | | | | |  | | | | | | | | |  | | | | | | | |  | | | | | | |  | | |  | |
