# Supplementary material for: Determinants of adolescents’ depression, anxiety, and somatic symptoms in Northwest Ethiopia: A non-recursive structural equation modeling
Source: PLoS One. 2024 Apr 10;19(4):e0281571. doi: 10.1371/journal.pone.0281571 (PMC11006201; doi:10.1371/journal.pone.0281571)
Supplement: S4 Table — (DOCX) [file pone.0281571.s005.docx]

**S4 Table: Intra cluster correlation coefficients for depression, anxiety, and somatic symptom among high and preparatory school adolescents in Northwest Ethiopia, 2022.**

| Clustering variable | ICC | | |
| --- | --- | --- | --- |
|  | Anxiety | Depression | Somatic symptom |
| Grade level | 7.33e-14 | 1.76*10^-4 | 2.2*10^-3 |
| School | 0.025 | 0.003 | 0.01 |
| School type | .016 | 2.72e-16 | 0.008 |
