# Supplementary material for: Determinants of adolescents’ depression, anxiety, and somatic symptoms in Northwest Ethiopia: A non-recursive structural equation modeling
Source: PLoS One. 2024 Apr 10;19(4):e0281571. doi: 10.1371/journal.pone.0281571 (PMC11006201; doi:10.1371/journal.pone.0281571)
Supplement: S5 Table — (DOCX) [file pone.0281571.s006.docx]

*S5 Table: validity and strength of instrumental variable for depression, anxiety, and somatic symptoms among adolescents in Northwest Ethiopia, 2022.*

| Outcome variable in the non-recursive loop | Instrumental variable | Instrumented | Cragg-Donald Wald F statistic | Anderson canon. corr. LM statistic p value | Sargan statistics  p- value | Remark |
| --- | --- | --- | --- | --- | --- | --- |
| Anxiety | Perceived academic ability  Physical trauma | Depression &  Somatic symptom | 4.06 | 0.004 | 0.00 |  |
|  |  | Depression | 38.5 | 0.000 | 0.617 | Strong and valid |
|  |  | Somatic symptom | 25.3 | 0.000 | 0.016 |  |
| Depression | Perceived social support  Death of loved one | Anxiety and somatic symptom | 2.27 | 0.033 | 0.000 |  |
|  |  | Anxiety | 30.0 | 0.000 | 0.644 | Strong and valid |
|  |  | Somatic symptom | 21.04 | 0.000 | 0.035 |  |
| Somatic symptom | Extra-school tutoring  Study time | Anxiety and depression | 0.7 | 0.226 | 0.00 |  |
|  |  | Anxiety | 0.8 | 0.470 | 0.08 |  |
|  |  | Depression | 1.8 | 0.156 | 0.40 |  |
