# Supplementary material for: Determinants of adolescents’ depression, anxiety, and somatic symptoms in Northwest Ethiopia: A non-recursive structural equation modeling
Source: PLoS One. 2024 Apr 10;19(4):e0281571. doi: 10.1371/journal.pone.0281571 (PMC11006201; doi:10.1371/journal.pone.0281571)
Supplement: S6 Table — (DOCX) [file pone.0281571.s007.docx]

*S6 Table: Factorial average item parceling for the construct of depression, anxiety, somatic symptom, stress and social support among adolescents in Northwest Ethiopia, 2022.*

| Construct | Items | Parcel |
| --- | --- | --- |
| Anxiety | Item 3(unable to stop worrying)  Item 5(being so restless) | Parcel 1 |
|  | Item 2 (worrying to much)  item 6(becoming easily annoyed) | Parcel 2 |
|  | item 1(Feeling nervous)  item 7(feeling afraid as something awful might happen)  item 4(trouble relaxing) | Parcel 3 |
| Depression | Suicidal ideation  Trouble sleep  Concentration | Parcel 1 |
|  | Energy  Moving slowly  Eating | Parcel 2 |
|  | Hopelessness  Feeling bad about self/family  Little interest | Parcel 3 |
| Somatic symptom | Feeling tired  Trouble sleep | Parcel 1 |
|  | Dizziness  Stomach pain  Lower Back pain | Parcel 2 |
|  | Chest pain  Headache | Parcel 3 |
| Stress | Item_09(angered)  Item_05(feel that things were going in your way)  Item 07(able to control irritation) | Parcel 1 |
|  | Item_10(feeling difficulties)  Item_04(felt confident)  Item_01(upset )  Item_06(unable to cope) | Parcel 2 |
|  | Item_03(felt stressed)  Item_08(felt on the top of things)  Item_02(unable to control important things) | Parcel 3 |
| Social support | Interest or concern people show on what you do  Peoples you can count if you have grate personal problem  Easiness to get practical help | Parcel 1 |
