# Supplementary material for: Determinants of adolescents’ depression, anxiety, and somatic symptoms in Northwest Ethiopia: A non-recursive structural equation modeling
Source: PLoS One. 2024 Apr 10;19(4):e0281571. doi: 10.1371/journal.pone.0281571 (PMC11006201; doi:10.1371/journal.pone.0281571)
Supplement: S7 Table — (DOCX) [file pone.0281571.s008.docx]

**S7 Table: *Depression among high school and preparatory school adolescents in Northwest Ethiopia, 2022 (N=1379).***

| Over the last two weeks, how often have you been bothered by any of the following problems | Not at all | Several days | More than half the days | Nearly every days |
| --- | --- | --- | --- | --- |
|  | Frequency (%) | Frequency (%) | Frequency (%) | Frequency (%) |
| Feeling down, depressed, irritable, or hopeless | 611(44.3) | 600(43.5) | 59(4.3) | 109(7.9) |
| Little interest or pleasure in doing things | 437(31.7) | 672(48.7) | 140(10.2) | 130(9.4) |
| Trouble falling or staying asleep, or sleeping too much | 531(38.5) | 525(38) | 155(11.2) | 168(12.2) |
| Poor appetite, weight loss, or overeating | 781(56.6) | 397(29) | 109(7.9) | 92(6.67) |
| Feeling tired, or having little energy | 605(43.87) | 537(38.9) | 109(7.9) | 128(9.3) |
| Feeling bad about yourself | 703(50.98) | 399(28.9) | 108(7.83) | 169(12.26) |
| Trouble concentrating on things like school work, reading or watching TV | 606(43.9) | 473(34.3) | 121(8.77) | 179(12.98) |
| Moving or speaking so slowly that other people could have noticed | 848(61.49) | 336(24.37) | 113(8.19) | 82(5.95) |
| Thoughts that you would be better off dead, or of hurting yourself in some way | 899(65.19) | 296(21.46) | 86(6.24) | 98(7.111) |
