# Supplementary material for: Determinants of adolescents’ depression, anxiety, and somatic symptoms in Northwest Ethiopia: A non-recursive structural equation modeling
Source: PLoS One. 2024 Apr 10;19(4):e0281571. doi: 10.1371/journal.pone.0281571 (PMC11006201; doi:10.1371/journal.pone.0281571)
Supplement: S8 Table — (DOCX) [file pone.0281571.s009.docx]

**S8 Table : *Anxiety among high school and preparatory school adolescents in Northwest Ethiopia, 2022 (N=1379)***

| Over the last 2 weeks, how often have you been  Bothered by the following problems | Not at all | Several days | More than half the days | Nearly every day |
| --- | --- | --- | --- | --- |
|  | Frequency (%) | Frequency (%) | Frequency (%) | Frequency (%) |
| Feeling nervous, anxious or on edge | 575(41.7) | 573(41.5) | 97(7.0) | 134(9.7) |
| Worrying too much about different things | 416(30.2) | 575(41.7) | 159(11.5) | 229(16.6) |
| Not being able to stop or control worrying | 571(41.4) | 512(37.1) | 143(10.4) | 153(11.1) |
| Trouble relaxing | 634(46) | 504(36.5) | 106(7.7) | (135(9.8) |
| Being so restless that it is hard to sit still | 769(55.8) | 388(28.1) | 96(7.0) | 126(9.1) |
| Becoming easily annoyed or irritable | 419(30.4) | 596(43.2) | 114(8.3) | 250(18.1) |
| Feeling afraid as if something awful might happen | 497(36.0) | 550(39.9) | 130(10.0) | 194(14.1) |
