# Supplementary material for: Determinants of adolescents’ depression, anxiety, and somatic symptoms in Northwest Ethiopia: A non-recursive structural equation modeling
Source: PLoS One. 2024 Apr 10;19(4):e0281571. doi: 10.1371/journal.pone.0281571 (PMC11006201; doi:10.1371/journal.pone.0281571)
Supplement: S9 Table — (DOCX) [file pone.0281571.s010.docx]

**S9 Table: *Somatic symptom among high school and preparatory school adolescents in Northwest Ethiopia, 2022 (N=1379)***

| During the past 7 days, how much have you been  Bothered by any of the following problems | Not at all | A little bit | somewhat | Quite a bit | Very much |
| --- | --- | --- | --- | --- | --- |
|  | Frequency (%) | Frequency (%) | Frequency (%) | Frequency (%) | Frequency (%) |
| Stomach or bowel problems | 883(64) | 330(23.9) | 89(6.5) | 45(3.3) | 32(2.3) |
| Back pain | 902(65.4) | 293(21.3) | 102(7.4) | 42(3.0) | 40(2.9) |
| Pain in your arms, legs, or joints | 891(64.6) | 275(19.9) | 128(9.3) | 51(3.7) | 34(2.5) |
| Headaches | 476(34.5) | 469(34.0) | 176(12.8) | 135(9.8) | 123(8.9) |
| Dizziness | 637(46.2) | 413(30.0) | 151(11.0) | 92(6.7) | 86(6.2) |
| Chest pain or shortness of breath | 966(70.0) | 204(14.8) | 98(7.1) | 54(3.9) | 57(4.1) |
| Feeling tired or having low energy | 726(52.7) | 377(27.3) | 140(10.2) | 68(4.9) | 68(4.9) |
| Trouble sleeping | 988(71.7) | 217(15.7) | 84(6.1) | 43(3.1) | 47(3.4) |
