# Supplementary material for: Determinants of adolescents’ depression, anxiety, and somatic symptoms in Northwest Ethiopia: A non-recursive structural equation modeling
Source: PLoS One. 2024 Apr 10;19(4):e0281571. doi: 10.1371/journal.pone.0281571 (PMC11006201; doi:10.1371/journal.pone.0281571)
Supplement: S1 Annex — (DOCX) [file pone.0281571.s011.docx]

***S1 Annex: Result of pilot study***

***Fig 1: Confirmatory factor analysis for measurement components of anxiety, depression, somatic symptom, stress and social support among high and preparatory school adolescents in Northwest Ethiopia, 2022(pilot).***


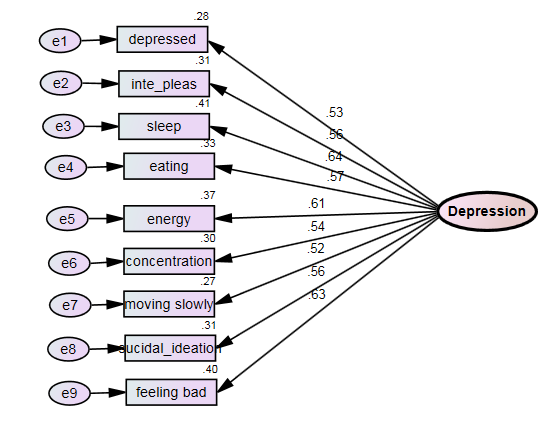

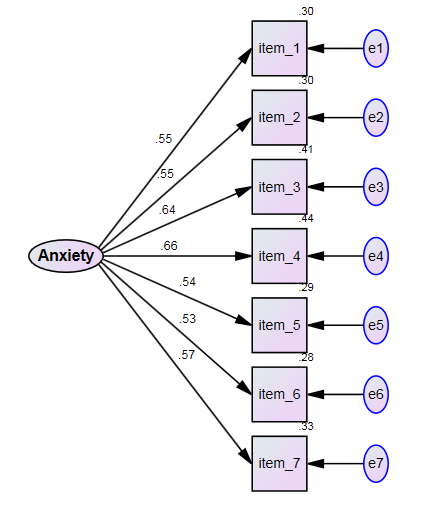


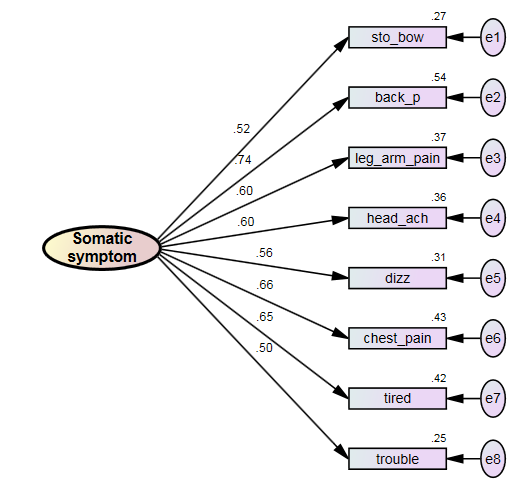

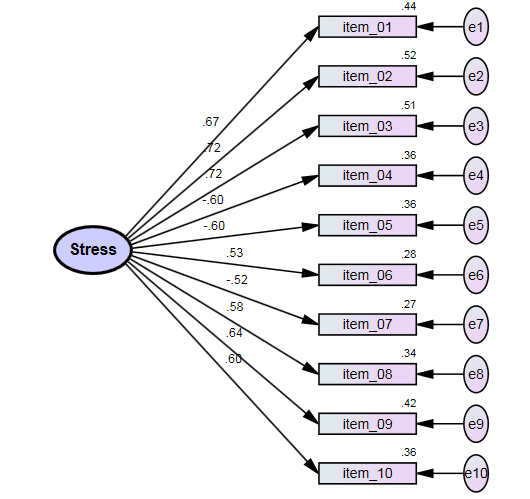

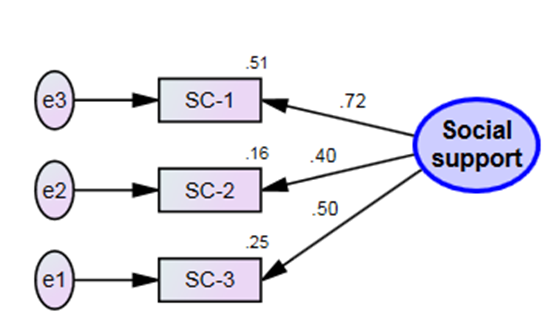


**Reliability and validity of the tool**

To validate the tool external participatory pilot study was conducted among 201 students, 177 from public school and 24 from private school. In the confirmatory factor analysis of the pilot study, reliability and validity of the tool were checked .Reliability of the tool was checked by using Cronbach alpha. The tool was reliable, since the Cronbach alpha values were above 0.7 for both overall and other particular factors except for social support (*Table1*).For social support, since it had low number item (<5) average inter-item covariance (AIIC) value were used, the value in this study were 0.4 which in the acceptable range (0.2 to 0.4) (141).

The convergent and discriminate validity of the tool was checked. For all factors .i.e. depression, anxiety, somatic symptom and stress all standardized factor loading were above 0.5(p value<0.05); for social support one item had loading of 0.4, but it was significant and its coefficient of determination was above 10%and significant (annexes VII).Even if the Average variance Extracted(AVE) was below 0.5, the convergent validity was maintained, since the composite reliability was above 0.6 (142)(*Table 1*). The discriminate validity of the tool were also evidenced, since all inter-factor correlations were less than the square root of AVE for each factor (*Table1*).

Table 2: Average variance extracted, composite reliability, and spearman rank correlation of tools used to measure anxiety,depression, somatic symptom ,stress and social support among high and preparatory school adolescents in Gondar town northwest Ethiopia 2022.

| Factors | AVE | CR | Correlation | | | | |
| --- | --- | --- | --- | --- | --- | --- | --- |
|  |  |  | Anxiety | Depression | Somatic symptom | Stress | Social support |
| Anxiety | 0.34 | 0.78 | 1.000 |  |  |  |  |
| Depression | 0.4 | 0.82 | 0.52 | 1.00 |  |  |  |
| Somatic symptom | 0.37 | 0.82 | 0.33 | 0.43 | 1.00 |  |  |
| Stress | 0.43 | 0.86 | 0.44 | 0.42 | 0.29 | 1.00 |  |
| Social support | 0.35 | 0.53 | -0.08 | 0.01 | -0.05 | 0.02 | 1.00 |

**Omitted variable bias**

Omitted variable bias (OVB) was also check in the pilot study. It occurs when a statistical model leaves one or more independent variable that is a determinant of the dependent variable and correlated with one or more of the included independent variables.Ramsey regression equation specification error test (RESET) was used to check OVB. Ramsey (RESET) value above 0.05 indicated the absence of omitted variable bias(143). In our case, the minimum Ramsey RESET p value was 0.2(*Table2*). Hence, there was no significant omitted variable bias.

Table 2: omitted variable bias test for anxiety, depression and somatic symptom considering all predictors in the conceptual framework

| Dependent variable | Ramsey RESET test P-value |
| --- | --- |
| Anxiety | 0.83 |
| Depression | 0.61 |
| Somatic symptom | 0.20 |
