# Supplementary material for: Determinants of adolescents’ depression, anxiety, and somatic symptoms in Northwest Ethiopia: A non-recursive structural equation modeling
Source: PLoS One. 2024 Apr 10;19(4):e0281571. doi: 10.1371/journal.pone.0281571 (PMC11006201; doi:10.1371/journal.pone.0281571)
Supplement: Attachment — Submitted filename: PONE-D-23-02244_R3_reviewerTS.pdf [file pone.0281571.s018.pdf]

PLOS ONE

Determinants of adolescents’ depression, anxiety, and somatic symptoms in Northwest Ethiopia: A Non-recursive structural equation modeling.

--Manuscript Draft--

**Manuscript Number:**	PONE-D-23-02244R3
**Article Type:**	Research Article
**Full Title:**	Determinants of adolescents’ depression, anxiety, and somatic symptoms in Northwest Ethiopia: A Non-recursive structural equation modeling.
**Short Title:**	Determinants of adolescents' depression, anxiety and somatic symptoms
**Corresponding Author:**	Zenebe Abebe Gebreegziabher, Masters<br>Debre Berhan University<br>Debre Berhan, Ethiopia ETHIOPIA
**Keywords:**	Anxiety, depression, somatic symptoms, adolescents, structural equation modeling, Ethiopia
**Abstract:**	Introduction: In low and middle-income countries, adolescent mental health is not only a major public health challenge but also a development concern. Depression and anxiety are the most common mental health disorders and somatic symptoms often co-exist with them. Adolescents with common mental health problems are associated with an increased risk of suicide, future unemployment, and poor quality of life. Little is known about the mental health of adolescents in Ethiopia. Thus, this study aimed to assess the determinants of depression, anxiety, and somatic symptoms among adolescents in Northwest Ethiopia, in 2022.<br>Methods: An institution-based cross-sectional study was conducted from June 8 to 24, 2022. Two-stage stratified random sampling was used to select 1407 adolescents in Northwest Ethiopia. Structured and standardized self-administered questionnaires were used to collect the data. Non-recursive structural equation modeling was employed to assess the direct, indirect, and total effects of predictors. Adjusted regression coefficients and corresponding 95% confidence intervals were used to interpret the strength of the association.<br>Results: The prevalence of depression, anxiety, and somatic symptoms were 28.21 (95% CI: 25.8, 31%), 25.05% (95% CI: 22.8, 27.5), and 25.24(95% CI: 23, 27.6%) respectively. Ever use of alcohol had a significant positive effect on depression [β= 0.14, 95% CI: 0.073, 0.201], anxiety [β= 0.11, 95% CI: 0.041, 0.188], and somatic symptoms [β= 0.12, 95% CI: 0.062, 0.211]. Stress had a significant positive effect on depression [β= 0.76, 95% CI: 0.642, 0.900], anxiety [β= 1.10, 95% CI: 0.955, 1.264], and somatic symptoms [β= 086, 95% C: 0.700, 1.025]. High levels of anxiety were substantially connected to higher levels of depression [β= 0.74, 95% CI: 0.483, 1.081], and depression had a direct positive influence on anxiety [β = 0.74, 95% CI: 0.508, 1.010].<br>Conclusion: In this study, the prevalence of depression, anxiety, and somatic symptoms was moderate. Alcohol use and stress were significantly related to depression, anxiety, and somatic symptoms. The bidirectional relationship between anxiety and depression was significant. Therefore, public health interventions should focus on the bidirectional relationship between depression and anxiety, as well as on identified factors to reduce the burden of mental illness in adolescents.
**Order of Authors:**	Zenebe Abebe Gebreegziabher, Masters
	Rediet Eristu, Masters
	Ayenew Molla, Masters
**Response to Reviewers:**	eviewer #2: In general the paper is greatly strengthened and I appreciate the close attention to the reviewers' comments and the changes made to the manuscript. My main remaining comments are 1. As another reviewer/editor has pointed out, the occurrence of anxiety, depression and somatic symptoms in adolescence is not a new hypothesis or finding. The unique contribution of the paper is documenting these issues in an Ethiopian adolescents and the exploration of potential contributing factors in that population. Hence, I would start the paper by making this point before moving

	on to define anxiety, depression (I would use this order) and somatic disorders, their prevalence in other regions, other parts of Africa and then Ethiopia, perhaps pointing out the dearth of research on Ethiopian adolescents in the conclusion of this section of the introduction. and 2. Efforts at writing in English which may not be the native language of the authors is alway admirable, in my view, and should not prohibit the publishing of important and interesting data as we have here. However, for maximum impact, it would make sense for the authors to have a reviewer read the paper for smooth flow in the English language as a final polish before submitting. In general, the paper is clear but this is scientific writing and precision in language counts. No criticism of the authors, just a suggestion to "professionalize" the writing further before being considered for acceptance. I wish the authors the best of luck in getting the paper accepted for publication.<br><br>Author:We tried to address the comments point by point
**Additional Information:**	
**Question**	**Response**
**Financial Disclosure**<br><br>Enter a financial disclosure statement that describes the sources of funding for the work included in this submission. Review the submission guidelines for detailed requirements. View published research articles from *PLOS ONE* for specific examples.<br><br>This statement is required for submission and **will appear in the published article** if the submission is accepted. Please make sure it is accurate.<br><br>**Unfunded studies**<br>Enter: *The author(s) received no specific funding for this work.*<br><br>**Funded studies**<br>Enter a statement with the following details:<br>• Initials of the authors who received each award<br>• Grant numbers awarded to each author<br>• The full name of each funder<br>• URL of each funder website<br>• Did the sponsors or funders play any role in the study design, data collection and analysis, decision to publish, or preparation of the manuscript?<br>• **NO** - Include this sentence at the end of your statement: *The funders had no role in study design, data collection and analysis, decision to publish, or preparation of the manuscript.*<br>• **YES** - Specify the role(s) played.<br><br>* typeset	The author(s) received no specific funding for this work.

Powered by Editorial Manager® and ProduXion Manager® from Aries Systems Corporation

**Competing Interests**<br><br>Use the instructions below to enter a competing interest statement for this submission. On behalf of all authors, disclose any competing interests that could be perceived to bias this work—acknowledging all financial support and any other relevant financial or non-financial competing interests.<br><br>This statement is required for submission and **will appear in the published article** if the submission is accepted. Please make sure it is accurate and that any funding sources listed in your Funding Information later in the submission form are also declared in your Financial Disclosure statement.<br><br>View published research articles from *PLOS ONE* for specific examples.<br><br>**NO authors have competing interests**<br><br>Enter: *The authors have declared that no competing interests exist.*<br><br>**Authors with competing interests**<br><br>Enter competing interest details beginning with this statement:<br><br>*I have read the journal's policy and the authors of this manuscript have the following competing interests: [insert competing interests here]*<br><br>* typeset	The authors have declared that no competing interests exist
**Ethics Statement**<br><br>Enter an ethics statement for this submission. This statement is required if the study involved:<br><br>• Human participants<br>• Human specimens or tissue<br>• Vertebrate animals or cephalopods	Ethical approval was obtained from the Institutional Review Board of the University of Gondar Institute of Public Health. The ethical approval letter was submitted to the Gondar town administrative educational department and all selected high schools and preparatory schools. Permission was obtained from school directors. Parental informed consent was waived as the study had no/minimal risk. Following permission from the school directors, each student was given a detailed participant information sheet and they completed an assent form to indicate their willingness to participate. Only students who signed an assent form were eligible to take part. Students with moderate to severe anxiety, depression, or somatic symptoms were referred to the University of Gondar

Powered by Editorial Manager® and ProduXion Manager® from Aries Systems Corporation

• Vertebrate embryos or tissues<br>• Field research<br><br>Write "N/A" if the submission does not require an ethics statement.<br><br>General guidance is provided below. Consult the submission guidelines for detailed instructions. **Make sure that all information entered here is included in the Methods section of the manuscript.**	Comprehensive Specialized Hospital Psychiatry Clinic. To ensure the privacy and confidentiality of participants, all information gathered was kept in a locked cabinet in the investigator's home, no personal identifiers were mentioned, and the collected data were used solely for research purposes. Overall, this study was carried out per the Helsinki Declaration.

Powered by Editorial Manager® and ProduXion Manager® from Aries Systems Corporation

**Format for specific study types**<br><br>**Human Subject Research (involving human participants and/or tissue)**<br>• Give the name of the institutional review board or ethics committee that approved the study<br>• Include the approval number and/or a statement indicating approval of this research<br>• Indicate the form of consent obtained (written/oral) or the reason that consent was not obtained (e.g. the data were analyzed anonymously)<br><br>**Animal Research (involving vertebrate animals, embryos or tissues)**<br>• Provide the name of the Institutional Animal Care and Use Committee (IACUC) or other relevant ethics board that reviewed the study protocol, and indicate whether they approved this research or granted a formal waiver of ethical approval<br>• Include an approval number if one was obtained<br>• If the study involved *non-human primates*, add *additional details* about animal welfare and steps taken to ameliorate suffering<br>• If anesthesia, euthanasia, or any kind of animal sacrifice is part of the study, include briefly which substances and/or methods were applied<br><br>**Field Research**<br><br>Include the following details if this study involves the collection of plant, animal, or other materials from a natural setting:<br>• Field permit number<br>• Name of the institution or relevant body that granted permission	
**Data Availability**<br><br>Authors are required to make all data underlying the findings described fully available, without restriction, and from the time of publication. PLOS allows rare exceptions to address legal and ethical concerns. See the PLOS Data Policy and FAQ for detailed information.	Yes - all data are fully available without restriction

Powered by Editorial Manager® and ProduXion Manager® from Aries Systems Corporation

A Data Availability Statement describing where the data can be found is required at submission. Your answers to this question constitute the Data Availability Statement and **will be published in the article**, if accepted.<br><br>**Important:** Stating 'data available on request from the author' is not sufficient. If your data are only available upon request, select 'No' for the first question and explain your exceptional situation in the text box.<br><br>Do the authors confirm that all data underlying the findings described in their manuscript are fully available without restriction?	
**Describe where the data may be found in full sentences. If you are copying our sample text, replace any instances of XXX with the appropriate details.**<br><br>• If the data are **held or will be held in a public repository**, include URLs, accession numbers or DOIs. If this information will only be available after acceptance, indicate this by ticking the box below. For example: *All XXX files are available from the XXX database (accession number(s) XXX, XXX.*).<br>• If the data are all contained **within the manuscript and/or Supporting Information files**, enter the following: *All relevant data are within the manuscript and its Supporting Information files.*<br>• If neither of these applies but you are able to provide **details of access elsewhere**, with or without limitations, please do so. For example:<br><br>*Data cannot be shared publicly because of [XXX]. Data are available from the XXX Institutional Data Access / Ethics Committee (contact via XXX) for researchers who meet the criteria for access to confidential data.*<br><br>*The data underlying the results presented in the study are available from (include the name of the third party*	All relevant data are within the manuscript and its Supporting Information files

and contact information or URL).<br>• This text is appropriate if the data are owned by a third party and authors do not have permission to share the data.<br><br>* typeset	
Additional data availability information:	Tick here if your circumstances are not covered by the questions above and you need the journal’s help to make your data available.

Powered by Editorial Manager® and ProduXion Manager® from Aries Systems Corporation

Determinants of adolescents' depression, anxiety, and somatic symptoms in Northwest Ethiopia: A Non-recursive structural equation modeling.

Zenebe Abebe Gebreegziabher[1*], Rediet Eristu[2], & Ayenew Molla[2]

[1] Department of Epidemiology and Biostatistics, School of Public Health, Asrat Woldeyes Health Science Campus, Debre Berhan University, Debre Berhan, Ethiopia

[2] Department of Epidemiology and Biostatistics, Institute of Public Health, College of Medicine and Health Sciences, University of Gondar, Gondar, Ethiopia

* Corresponding author

Email: zenebeabe7054@gmail.com (ZA)

Abstract

Introduction: In low and middle-income countries, adolescent mental health is not only a major public health challenge but also a development concern. Depression and anxiety are the most common mental health disorders and somatic symptoms often co-exist with them. Adolescents with common mental health problems are associated with an increased risk of suicide, future unemployment, and poor quality of life. Little is known about the mental health of adolescents in Ethiopia. Thus, this study aimed to assess the determinants of depression, anxiety, and somatic symptoms among adolescents in Northwest Ethiopia, in 2022.

Methods: An institution-based cross-sectional study was conducted from June 8 to 24, 2022. Two-stage stratified random sampling was used to select 1407 adolescents in Northwest

Ethiopia. Structured and standardized self-administered questionnaires were used to collect the data. Non-recursive structural equation modeling was employed to assess the direct, indirect, and total effects of predictors. Adjusted regression coefficients and corresponding 95% confidence intervals were used to interpret the strength of the association.

Results: The prevalence of depression, anxiety, and somatic symptoms were 28.21 (95% CI: 25.8, 31%), 25.05% (95% CI: 22.8, 27.5), and 25.24(95% CI: 23, 27.6%) respectively. Ever use of alcohol had a significant positive effect on depression [β= 0.14, 95% CI: 0.073, 0.201], anxiety [β= 0.11, 95% CI: 0.041, 0.188], and somatic symptoms [β= 0.12, 95% CI: 0.062, 0.211]. Stress had a significant positive effect on depression [β= 0.76, 95% CI: 0.642, 0.900], anxiety [β= 1.10, 95% CI: 0.955, 1.264], and somatic symptoms [β= 086, 95% C: 0.700, 1.025]. ~~High levels of anxiety were substantially connected to higher levels of depression [β= 0.74, 95% CI: 0.483, 1.081], and~~ depression had a direct positive influence on anxiety [β = 0.74, 95% CI: 0.508, 1.010].

Conclusion: In this study, the prevalence of depression, anxiety, and somatic symptoms was moderate. Alcohol use and stress were significantly related to depression, anxiety, and somatic symptoms. The bidirectional relationship between anxiety and depression was significant. Therefore, public health interventions should focus on the bidirectional relationship between depression and anxiety, as well as on identified factors to reduce the burden of mental illness in adolescents.

Keywords: Anxiety, depression, somatic symptoms, adolescents, structural equation modeling, Ethiopia

Introduction

Mental health disorders account for 16% of the global burden of disease and injuries and more than 80% of people with mental health disorders live in low and middle-income countries, with an increase in incidence and mortality [1].

Adolescence marks the transition from infancy to adulthood, involving physical, psychological, social, and cognitive ~~development~~, ~~leading to~~ [illegible]ble confusion, stress, and emotional instability [2]. Adolescents are especially vulnerable to developing mental illness [3]. In this population mental health disorders increased by 32.18% from 1999 to 2019 [4]. According to the World Health Organization, mental health issues impact 10–20% of adolescents in the world [5]. In low and middle-income countries, adolescent mental health is not only a major public health challenge but also a development concern [6].

Human suffering and financial costs associated with mental health disorders are substantial and growing globally [7]. More than 12 billion working days are lost annually as a result of mental illness. More than diabetes, cancer, and respiratory diseases combined, it is predicted that between 2011 and 2030, the world economy will lose 16 trillion US dollars due to mental illness [6]. Depression and anxiety are among the common mental health disorders [8], and often somatic symptoms coexist with them [9, 10].

Depression, which is the second most common mental health disorder in adolescents, is characterized by loss of interest or pleasure, feelings of guilt or low self-worth, feelings of tiredness, disturbed sleep or appetite, and poor concentration and sadness [11]. In 2018, over one million adolescents died from preventable causes, with depression playing a significant role [12]. In Asia, its prevalence ranges from 24.3% to 57.7% [13], and in Sub-Saharan Africa, it ranges from 15.5% [14] to 45.90% [15, 16]. In Ethiopia, 28% of adolescents in Jimma [17] and 36.2%

of adolescents in Aksum [18] were depressed. Previous studies documented that age [16], female sex [17, 19-21], being in public school [22], high family academic pressure [23, 24], poor self-rated academic ability [25, 26], alcohol use [27, 28], cigarette smoking [29, 30], stress [31-33], somatic symptoms [17, 19, 20, 31, 34, 35], and anxiety [36, 37] had a positive relation with depression. Other factors, such as social support [25, 26, 34, 38], and family education [35] had, negative associations with depression.

Anxiety is characterized by emotional feelings of excessive fear, nervousness, avoiding threats in the environment perceived by them, and physical symptoms such as fast respiration, increased blood pressure, and tightness of the chest [39, 40]. Globally, 301.39 million people are affected by anxiety [41]. It is the leading cause of mental disorders in the world, accounting for approximately 28.68 million disability-adjusted life years in the global burden of disease [42]. Anxiety is the most common mental health disorder in adolescents [43], with a high prevalence in developed as well as in developing countries [44]. In adolescents, it ranges from 42.1% [45] to 80.85% in Asia [46], and in sub-Saharan Africa, 29.8% of adolescents had anxiety [3]. A study conducted in Kenya showed that 37.99% of high school adolescents had anxiety [16]. A systematic review conducted among children and youth in Ethiopia revealed that general anxiety disorder ranges from 0.5-23% [47]. In combination with depression, anxiety contributes to 45% of the overall burden of disease [48]. Different pieces of literature reported that female sex [46, 49], age [16, 45], smoking [30, 50], alcohol use, low self-rated academic ability [45, 51], family academic pressure [23], stress [52, 53], somatic symptom disorder [54] and depression [55] have a positive relationship with anxiety.

A somatic symptom is characterized by a comprehensive list of symptoms such as pain, breathlessness, numbness, palpitation, tiredness, headache, dizziness, and gastroenterological

problems. Somatic symptom disorder is a disorder in which individuals excessively or disproportionally think or experience feelings about the symptoms, which results in significant disturbance in daily life [56]. Depression and anxiety often coexist with somatic symptoms [57-59], which is one of the remarkably widespread issues in primary health care and subspecialty settings [10]. Somatic symptoms result in more than half of the patient visits to primary health care [60]. Its prevalence is estimated to be 5 to 7% [61] in the general population and 5 to 30% [62, 63] in adolescents. Somatic symptoms delay the diagnosis of depression and anxiety because most people seek medical attention for bodily symptoms. Finally, individuals with somatic symptoms may experience physical harm from unnecessary medical procedures [64]. Different scholars reported that female sex [65-67], being in private school [65], physical inactivity [68, 69], extra school tutoring [70], stress [71-73], anxiety [66, 74, 75] and depression [66, 76, 77] had significant positive relations with somatic symptoms.

Although mental health is a key part of sustainable development goals that affect every other goal [6], the prevalence of mental health disorders is still high [46]. To decrease the burden of mental illness, targeting adolescents is important; since more than half of mental health problems start at this age [6], and 50% of adolescent mental health problems will proceed to adulthood [78]. Adolescents in low and middle-income countries are disproportionally affected by mental health disorders; however, there is limited data on the prevalence and determinants of mental illness in Sub-Saharan Africa [79, 80].

In Ethiopia, although mental health services were included in the national health policy, there is a paucity of evidence on the prevalence and determinants of anxiety, somatic symptoms, and their relation with depression. Even studies conducted outside of Ethiopia had methodological flaws, as they used logistic or linear regression to assess multiple mental health

outcomes, which cannot address the bidirectional relationship between outcome variables and the indirect effect of predictors. In such cases, non-recursive structural equation modeling, which is a multivariate statistical framework used to measure the complex relationships between different observed and latent variables simultaneously is preferable. Adolescents in northwest Ethiopia are especially vulnerable to mental illness, due to repeated internal conflicts in northern Ethiopia [81].

~~Therefore, this study intended to fill this gap by assessing the~~ prevalence, ~~relationship~~, and determinants of depression, anxiety, and somatic symptoms among high school and preparatory school adolescents in Gondar town by using non-recursive structural equation modeling. The findings from this study will help psychologists, psychiatrists, policymakers, students, the community, and the government to tackle the increasing burden of mental health problems. Moreover, it will be used as input for sustainable development and as baseline data for further research.

Methods and materials

Study design and context

An institution-based cross-sectional study was conducted from June 8 to June 24, 2022, among high and preparatory school adolescents in Gondar town, Northwest, Ethiopia. It is located 728 kilometers from Ethiopia's capital city of Addis Ababa, and 180 kilometers from Bahir Dar, the capital city of the Amhara regional state. There are 395, 000 residents [82], and 48 healthcare facilities including one comprehensive specialized hospital, eight health centers, one private general hospital, fifteen specialist clinics, fifteen medium clinics, and eight primary clinics. Adolescents comprise more than 25% of the Ethiopian population and the health of

adolescents determines the future development of the country. According to the town administrative educational department report, the town has seventeen (twelve public and five private high schools), with a total of 24,308 students. Among them, 2408 students were from private schools (1457 girls and 951 boys) and 21900 students were from public schools (12052 girls and 9848 boys).

Participants

All high and preparatory school adolescents in Gondar town who were registered for the second semester of the 2021-2022 academic year were included in the study. Night preparatory and high school students and students who were transferred in from other areas after January 8, 2022, were excluded.

The general rule for sample size calculation in structural equation modeling ranges from 5 to 20 times per free parameter [83]. In this study, a 5:1 ratio was used to obtain the minimum adequate sample size. Based on the hypothesized model ***Fig 1***, a total of 134 free parameters were available (***Fig 1***).

Considering 134 free parameters, the sample size was

134*5=670: Since the sampling procedure is multistage sampling with 2 stages, a design effect of 2 was considered.

Therefore, the sample size becomes 670*2 =1340

After adjusting for 5% for non-response rate, the final sample size became

1340+1340*5%=**1407**

Fig 1: Hypothetical structural equation modeling on the prevalence and determinants of depression, anxiety, and somatic symptoms among high school and preparatory school adolescents in Northwest Ethiopia, 2022 (both measurement and structural model): *Circles indicate latent variables or error terms or disturbances, rectangles indicate observed variables, single arrows indicate factor loadings or regression coefficients, and double arrows indicate the covariance between latent variables. A1-A7=items of anxiety, D1-D9=items for depression, S1-S8=items for somatic symptoms, ST1-ST10=items for stress, and e=error term.*

In this study, a two-stage stratified random sampling technique was used to select participants. A study conducted in Telangana state, India, showed that adolescents in public schools had a higher proportion of depression than those in private schools [22]. As a result, stratification by school type was performed and study subjects were proportionally allocated. Then, two private schools and four public schools were selected randomly. After that, heterogeneity by grade level was considered and, stratification was performed by grade level (***Fig 2***).

Fig 2: Diagrammatical representation of the sampling procedure that was used to assess the prevalence and determinants of depression, anxiety, and somatic symptoms among high school and preparatory school adolescents in Northwest Ethiopia, 2022. N indicates the total number of students.

Variables of the study

Outcome variables (endogenous constructs):

Depression, anxiety, and somatic symptoms

Independent variables: summary of key variables used in the study**: S1 Table 1**

Data collection procedures and tools

Primary data in pen and pencil format were gathered using a structured questionnaire through a self-reported questionnaire for those who could see and by face-to-face interview for 3 individuals who could not see. Four trained data collectors with a first degree in public health were assigned for data collection.

The questionnaire was designed to gather data on socio-demographic characteristics, behavioral factors, academic-related factors, relationship-related factors, clinical factors, depression, anxiety, somatic symptoms, and stress domains (**Table 1**).

Table 1: Instruments used for the determinants of depression anxiety, somatic symptom, their description and criteria for categorization

**Variables**	**Instruments**	**Description**	**Criteria for categorization**	**Reference**
Depression	Patients Health Questionnaire 9 for adolescents (PHQ-9A)	It is a depression screening tool with 9 items; each item was measured by a Likert scale ranging from 0 "not at all" to 3 "nearly every day" and its total score ranged from 0 to 27.	Students were classified as having depression if their total score on the PHQ-9A was 10 or higher.	[84]
Anxiety	General Anxiety Scale-7 (GAS-7)	A brief measure of anxiety contains seven items, which are rated on four-point Likert scales that range from 0 "not at all" to 3 nearly every day”. Its summed score ranges from 0- 21.	Students were classified as anxious if their total score on the GAS-7 was 10 or higher.	[85]
Somatic	Somatic	It contains a total of 8 items,	Based on the SSS-8 score,	[86]

symptom	symptom scale-8 (SSS-8)	each item was measured by a Likert scale ranging from 0 “not at all” to 4”very much’; a total scoring range from minimal 0 to maximum 32.	students were classified as having low 4 to 7, medium 8 to 11, high 12 to 15, and very 16 to 32 very high somatic symptom levels.	
Stress	Perceived Stress Scale 10 (PSS-10)	It contains 10 items. Each item scored with an ordinal scale of 0–4, where 0 represents “never” and 3 represents “very often”. Individual scores range from 0 to 40.	Students who have a total perceived stress scale 10 scores of 0-13, 14-26, and 27-40 were categorized as having low, moderate, and high perceived stress levels respectively.	[87]
Social support	Oslo Social Support Scale three	It has 3 items; with a Likert scale that ranges from 1 to 4 for the first item (number of people you can count if you face a great personal problem) and 1 to 5 ranges for the rest items. It’s summed score ranges from 3 to 14.	Students were categorized as having poor social support 3–8 score, moderate social support 9–11 score, and strong social support 12-14 score.	[88].
Ever use of alcohol	Alcohol, smoking, and substance involvement test(ASSIST) was used to assess current and ever use of alcohol	Do you have a history of alcohol (like beer, Tella, Katikala, Wine) intake in your lifetime?<br><br>Do you drink alcohol within the past 3 months?	Respondents who answered yes were considered positive for ever-use of alcohol<br><br>Students who answered yes were considered as positive for current alcohol user	[89]

Physical activity	Self-reported physical activity assessment for adolescents was used	In the past week, how many days did you exercise for at least 60 minutes until you felt sweaty or shortness of breath	Respondents who answered 2 days and above were categorized as physically active and those who answered one day and not were categorized as physically inactive.	[90]

Data quality assurance

~~To maintain the quality of the data, the questionnaire that was developed in the English language was translated into Amharic and re-translated into English by another person to check consistency. Four public health experts and two psychiatrists checked the face validity of the tool. Moreover, a pilot study was conducted among 201 students (177 from public schools and 24 from private schools) to check the validity and reliability of the tool. Reliability was checked by using Cronbach's alpha and the tool was reliable since Cronbach's alpha values were above 0.7 for the overall and particular factors except for social support. For social support, since it had a small number of items (<5), the average inter-item covariance (AIIC) value was used, which was 0.4, which is in the acceptable range (0.2 to 0.4). The convergent and discriminate validity of the tool was also checked. The convergent validity was maintained since all standardized factor loadings were significant (p-value <0.05) and the composite reliability was above 0.6. The discriminate validity of the tool was also evidenced since all inter-factor correlations were less than the square root of AVE **S3 annex 1**. A pilot study was also used to check the way that each questionnaire was understood by different participants and modifications were made accordingly based on the feedback. Additionally, the Ramsey regression equation specification error test (RESET) was used to check omitted variable bias in the pilot study. In our study, the minimum~~

Ramsey RESET p value was 0.2 **S3 annex 1**. Hence; there was no significant omitted variable bias [91].

Data processing, model building, and analysis

Collected data were coded and entered into Epi-data software version 4.6, and exported to STATA version 16 and AMOS version 21 for further analysis. Non-recursive structural equation modeling was employed to assess the complex relationship between different latent and observed variables. Model assumptions such as: specified and identified model, multivariate normality, missing, outlier, sample size adequacy, independency, common method bias, and strong and valid instrumental variable were checked.

Specified model: In this study, we specified the model after extensive literature searching and reading as presented in ***Fig 1***.

Identified Model: In this study, identification of non-recursive structural equation modeling with complete correlation of error terms was used. There are three methods of identification in non-recursive structural equation modeling; which are: the presence of unique instrumental variables, the order condition, and the rank condition. Among these, only the rank condition is sufficient for identification, and the remaining two are necessary, but not sufficient [92]. In this study, all endogenous variables in the reciprocal loop had unique instrumental variables. Order condition: the number of excluded variables for that specific endogenous variable minus the total number of endogenous variables minus one should be greater than or equal to 0 [92]. In our model, to satisfy the order condition, each endogenous variable in the recursive loop should have at least 3-1 =2 excluded variables. For depression, anxiety, and somatic symptoms 9, 8, and 11 variables were excluded respectively. Therefore, the model fulfills the requirement of the order

condition. Rank condition: In this particular study, there are 3 endogenous variables in the non-recursive loop 17 exogenous variables, and one endogenous variable out of the feedback loop (***Fig 1***). Rank conditions were determined using the system matrix. To formulate the system matrix, a separate equation for each endogenous variable in the feedback loop is mandatory. Therefore, the equations for depression, anxiety, and somatic symptoms disorder are presented as follows:

$$\textbf{\textit{Depression}}(\boldsymbol{y}1) = \text{x2 alcohol use} + \text{x3 chronic illness} + \text{x4 physical activity} + \text{x5 family mental ilness} + \text{x7 school ty} + \text{x11 death of beloved one} + \text{x12 social support} + \text{x16family pressur} + \text{x18 stress} + \text{x20}(\text{y2}) + \text{x21}(\text{y3})\ \text{somaticsymptom}$$

$$\textbf{\textit{Anxiety}}(\boldsymbol{y}2) = \text{x2 alcohol use} + \text{x3 chronic illness} + \text{x4 physical activity} + \text{x5 family mental ilness} + \text{x7 school type} + \text{x13 physical trauma} + \text{x17 acadamic ability} + \text{x18 stress} + x19(y1) + x21(y3)$$

$$\textbf{Somaticsymptom}(\textbf{y}3) = \text{x2 alcohol use} + \text{x3 chronic illness} + \text{x4 physical activity} + \text{x5 family mental ilness} + \text{x7 school type} + \text{x14 study time} + \text{x15 extra school tutoring} + \text{x18 stress} + \text{x19}(\text{y1}) + \text{x20}(\text{y2})$$

Rank conditions in the system matrix below begin by constructing a system matrix based on the above equations. Endogenous variables are presented in the row and all variables are presented in the column. In each row, zero or one appears in the column that corresponds to that row. One indicates that the variable represented by the column has a direct effect on the endogenous variable in that row or on the endogenous variable itself, and 0 indicates the excluded variables for that specific endogenous variable in that row [92].

$$\begin{bmatrix} x & 2 & 3 & 4 & 5 & 6 & 7 & 8 & 9 & 10 & 11 & 12 & 13 & 14 & 15 & 16 & 17 & 18 & y1 & y2 & y3 \\ y1 & 1 & 1 & 1 & 1 & 0 & 1 & 0 & 0 & 0 & 1 & 1 & 0 & 0 & 0 & 1 & 0 & 1 & 1 & 1 & 1 \\ y2 & 1 & 1 & 1 & 1 & 0 & 1 & 0 & 0 & 0 & 0 & 0 & 1 & 0 & 0 & 0 & 1 & 1 & 1 & 1 & 1 \\ y3 & 1 & 1 & 1 & 1 & 0 & 1 & 0 & 0 & 0 & 0 & 0 & 0 & 1 & 1 & 0 & 0 & 1 & 1 & 1 & 1 \end{bmatrix}$$

The following steps were followed for the identification of rank condition

1. Start with the first row of the system matrix. Cross out all entries in that row and cross out any column in the system matrix with one in that same row.

2. Simplify the reduced matrix further by deleting any row with all zeros entries; delete any row that is an exact duplicate of another or can be reproduced by adding other rows (i.e., it is a linear combination of other rows). The number of remaining rows is the rank.

If the rank of the reduced matrix is larger than or equal to the sum of the endogenous variables in the recursive loop minus one, then the rank condition is satisfied [92].

The rank condition for this particular study became;

For depression (y1), after we cross the entire first row and the entire column that contains 1 in the row:

The reduced matrix becomes:

$$\begin{bmatrix} 1 & 0 \\ 0 & 1 \end{bmatrix} \text{Rank=2}$$

For anxiety (y2), after we cross the entire second row and the entire column that contains 1 in that row:

The reduced matrix becomes:

$$\begin{bmatrix} 1 & 0 \\ 0 & 1 \end{bmatrix} \quad \text{Rank=2}$$

For Somatic symptom (y3), after we cross the entire third row and the entire column that contains one in that row:

The reduced matrix becomes:

$$\begin{bmatrix} 1 & 0 \\ 0 & 1 \end{bmatrix} \text{Rank=2}$$

The hypothesized model was identified since all endogenous variables have a rank of two; which is equal to the number of endogenous variables in the feedback loop minus one.

Multivariate normality, outliers, and missing: Full information maximum likelihood estimation was used. Multivariate normality was assessed using Mardia's kurtosis and its critical ratio. The data were not normally distributed, since Mardia's kurtosis was above 7 and its critical ratio was above 5 [92]. Although item parceling and outlier deletion were attempted, there was no improvement. As a result, bootstrap maximum likelihood estimation with 3000 samples was used.

Mahalanobis distance p values less than 0.001 based on chi-square distribution were used to declare observation as a multivariate outlier [93]. There were 62 observations with Mahalanobis distance p-values less than 0.001. The data were examined to determine whether they were outliers or caused by data entry errors. However, this was not due to a data entry error, and as mentioned above, multivariate normality did not improve significantly with their exclusion. Therefore, those outlier observations were kept to maintain the study's power.

From the total observations, there were six observations with missing values. All missing values were from predictors and had no relationship with the outcome variable; thus, list-wise deletion was performed because they constituted less than 5% of the total sample and were considered missing completely at random, so 1373 observations were used for the final analysis [94].

Sample size adequacy and strength of correlation: The critical ratio for Bartlett's test of sphericity in this study was high and significant for all constructs. KMO was above 0.7 overall, as well as for specific constructs. Hence, the sample is sufficient, the population matrix was not identified, and factor analysis was evidenced [92] (**Table 2**).

Table 2: *KMO and Bartlett's test of sphericity for the measurement of depression, anxiety, and somatic symptoms among high and preparatory school adolescents in Northwest Ethiopia, 2022.*

Factors	KMO	Bartlett's tests of sphericity	
		Chi-square	P value
Anxiety	0.88	3100	0.0001
Depression	0.87	2391	0.0001
Somatic	0.85	2155	0.0001
Stress	0.86	3354	0.0001
Overall	0.94	13938	0.0001

Independency of observations: The intra-class correlation coefficients (ICCs) were used to test the independence of observations. School type and grade level were employed as clustering variables. The ICC was less than 0.1 in our study (**S1 Table 2)**, and a classical structural equation model was used.

Common Method Bias: Harman's one-factor test was used to check common method bias (CMB). There was no CMB in this study since the overall variance covered by one factor was 25.2%, which is below the suggested cut-off (50%) [95].

Strong and valid instrumental variables: The strength and validity of instrumental variables were checked by using the Cragg-Donald Wald F statistic and Sargan Hansen test, respectively. A Sargan Hansen test P-value greater than 0.05 was used to ponder valid instruments, and F-test values above 10 were used as a cut-off point to declare strong IVs [96]. In the present study, the strength and validity of the instrumental variables between anxiety and somatic symptoms, and between somatic symptoms and depression were invalid and weak. As a result, the non-recursive paths from somatic symptoms to anxiety and depression were excluded; since deleting a non-recursive path is one treatment for a weak and faulty instrument. Finally, an

identified model with valid and strong instrumental variables in a non-recursive loop was preserved in **S1 Table 3**.

Ethical consideration

Ethical approval was obtained from the Institutional Review Board of the University of Gondar Institute of Public Health. ~~The ethical approval letter was submitted to the Gondar town administrative educational department and all selected high schools and preparatory schools.~~ Permission was obtained from school directors. Parental informed consent was waived as the study had no/minimal risk [97]. Following permission from the school directors, each student was given a detailed participant information sheet and they completed an assent form to indicate their willingness to participate. Only students who signed an assent form were eligible to take part. Students with moderate to severe anxiety, depression, or somatic symptoms were referred to the University of Gondar Comprehensive Specialized Hospital Psychiatry Clinic. ~~To ensure the privacy and confidentiality of participants, all information gathered was kept in a locked cabinet in the investigator's home, no personal identifiers were mentioned, and the collected data were used solely for research purposes. Overall, this study was carried out per the Helsinki Declaration.~~

Results

Socio-demographic and clinical factors of respondents

Out of 1407 randomly selected individuals, 1379 completed the questionnaire; hence, the response rate was 98%. The mean (± SD) age of the respondents was 17.23 ± 1.25 years with a range of 15 to 19 years. The majority, 1238(89.78%) of the respondents were from public schools, and regarding residence, two-thirds 915(66.35%) of respondents were from urban areas.

Approximately half, (654, 47.43%) of the respondents had moderate stress. The majority, 1237 (89.7%), of the respondents did not have a history of chronic illness **(*Table 3*).**

Table 3: Socio-demographic and clinical characteristics of high and preparatory school adolescents in Northwest Ethiopia, 2022 (n=1379).

Variable	Frequency	Percentage (%)
**Sex**		
Male	536	38.87
Female	843	61.13
**Age**		
15	140	10.20
16	291	21.19
17	297	21.63
18	405	29.50
19	240	17.48
**School type**		
Public	1238	89.78
Private	141	10.22
**Grade Level**		
Grade 9	544	39.45
Grade 10	325	23.57
Grade 11	296	21.46
Grade 12	214	15.52
**Residence**		
Urban	915	66.35
Rural	464	33.65
**Physical trauma**		
No	1076	78.03
Yes	303	21.97
**Medically confirmed chronic illness**		
No	1237	89.7
Yes	142	10.3
**Stress**		
Low	653	47.35
Moderate	654	47.43
High	72	5.22

**Family history of mental illness**		
No	1278	92.68
Yes	101	7.32

Behavioral, academic, and relationship-related factors

More than one-third, (493, 35.75%) of the respondents had a history of alcohol consumption. Regarding physical activity, only 224(16.24%) of the respondents were physically active. The majority, 656 (47.57%) of the respondents, had good self-rated academic ability. When we see perceived social support, two-fifths of 564(40.9%) of the respondents had low perceived social support (***Table 4***).

Table 4: Behavioral, academic, and relation-related factors of high and preparatory school adolescents in Northwest Ethiopia, 2022 (n=1379).

Variables	Frequency	Percentage (%)
**Physical activity**		
Inactive	1155	83.76
Active	224	16.24
**Ever use of alcohol**		
No	886	64.25
Yes	493	35.75
**Extra school tutoring*(n=1378)**		
No	1011	73.3
Yes	367	26.7
**Self-rated academic ability**		
Very poor	39	2.83
Poor	70	5.08
Good	656	47.57
Very good	390	28.28
Excellent	224	16.24
**Father education *(n=1377)**		
Can't read and write	266	19.29
Grade 1- 8	488	35.43

Grade 9-12	308	22.34
Diploma and above	315	22.84
**Mother education* (n=1378)**		
Can’t read and write	429	31.12
Grade 1- 8	436	31.63
Grade 9-12	277	20.10
Diploma and above	236	1.13
**Family academic pressure**		
Not at all	524	38
Low	375	27.19
Medium	144	10.44
High	336	24.37
**Death of a loved one in the past 6 months**		
No	1058	76.72
Yes	321	23.28
**Social support**		
Poor	564	40.9
Medium	559	40.54
Good	256	18.56
**Current alcohol use(n=1377)***		
No	368	26.73
Yes	1009	73.27
	Median	IQR
**Study time in hours(n=1379)**	3	±3
**Time spent for extra school tutoring** in hour(n=367)	**2**	±2

* Indicates variables with missing observations

Prevalence of depression

In this study, the overall prevalence of self-reported depression was 28.21(95% CI= 25.8, 31%)**.** Regarding severity, 34.8% had mild depression, 18.5% had moderate depression, 7.72% had moderately severe depression and 2.04% o had severe depression. More than one-third, 34.8% of participants had a feeling of hurting themselves or betting off dead, and more than two-thirds of the respondents (68.3%) had little interest or pleasure in doing things **(S1 Table 4)**.

Prevalence of Anxiety

The overall prevalence of self-reported anxiety in this study was 25.05% (95% CI: 22.8, 27.5%). Regarding the level of anxiety, 34.08% of respondents had mild anxiety, 16.82% had moderate anxiety, and 8.27% had severe anxiety. Among the total respondents, 16.61% worried too much about different things nearly every day, and 11.54% were not able to stop worrying too much nearly every day. More than half (58.38%) of the respondents had a feeling of anxiety or nervousness (**S1 Table 5)**.

Prevalence of somatic symptoms

In this study, the overall prevalence of self-reported SSD was 25.24% (95% CI= 23, 27.6%). Regarding its level, among the total respondents, 27.27% had mild, 15.88% had moderate, 8.19% had high, and 6.38% had very high somatic symptoms. Regarding the response to specific indicators, approximately, two-thirds (65.42%) of the participants had headaches, more than one-half (53.68%) of the respondents had dizziness and 35.97% of the respondents had abdominal pain or gastrointestinal problem**s (S1 Table 6)**.

Structural equation modeling

Confirmatory factor analysis (CFA) was computed to test the measurement model. The model fit measures (CMIN/DF, CFI, TLI, and RMSEA) were used to assess the model's overall goodness of fit. In the hypothesized measurement mod**el S2 Fig 1,** the goodness of fit was below the acceptable level: CMIN/DF=5.10, CFI=0.81 and RMSEA=0.05. To improve the fit of the model, factorial item parceling was performed [98] **S1 Table 7.** After that, the model fits were improved (CMIN/DF=2.60, CFI=0.99, TLI=0.99, and RMSEA=0.001(***Fig 3*)).**

Fig 3: Final measurement model for the constructs of anxiety, depression, somatic symptoms, stress, and social support.

Key: a =anxiety, d= depression, SO=somatic symptoms, st=stress, SS= social support, e =error term, and bidirectional arrow indicates covariance

For the model containing the structural and measurement parts, model comparisons were performed using AIC, CMIN/DF, TLI, CFI, and RMSEA. The model with only significant variables with significant disturbance after the parcel; which had small AIC, small CMIN/DF, and RMSEA, and large CFI and TLI was selected as a good fitted model (***Table 5***).

Table 5: Model selection for determinants of depression, anxiety, and somatic symptoms among high and preparatory school adolescents in Northwest Ethiopia, 2022.

Models	AIC	CMIN/DF	CFI	TLI	RMSEA	Remark
A model with all predictors and covariance after the parcel	2468	6.8	0.8	0.7	0.1	
A model with only significant variables with significant disturbance after the parcel	951	3.00	0.94	0.92	0.027	**selected**

Factors related to depression, anxiety, and somatic symptoms

Self-rated academic ability, perceived social support, physical trauma, death of loved one, ever use of alcohol, having medically confirmed chronic illness, sex, family pressure, school type, and stress were all significantly related to anxiety, depression, and somatic symptoms, either directly or indirectly. There was a significant bidirectional relationship between anxiety and depression (***Fig 4***, ***Table 6***, ***Table 7*****, and *****Table 8***).

Fig 4: Standardized structural equation modeling for factors related to anxiety, depression, and somatic symptoms among high and preparatory school adolescents in Gondar town, Northwest, Ethiopia, 2022.

Factors related to depression among high and preparatory school adolescents in Gondar town, 2022.

Perceived social support, having a history of the death of a loved one within the past six months, and anxiety had a significant direct effect on depression. However, having medically confirmed chronic illness, having a history of alcohol use, perceived family academic pressure, sex, stress, self-rated academic ability, and having a history of physical trauma had a statistically significant indirect relationship with depression. School type and anxiety were significantly related to depression both directly and indirectly.

Anxiety [adjusted β= 0.74, 95% CI: 0.483, 1.081] and perceived social support [adjusted β= -0.13, 95% CI: -0.229,-0.029] had a positive direct effect on depression. Self-rated academic ability had a significant indirect effect [adjusted β=-0.05, 95% CI: -0.083,-0.031] on depression (***Table 6***).

Table 6: Direct, indirect, and total effects of socio-demographic, behavioral, relationship-related factors and clinical factors on depression among adolescents in Northwest Ethiopia, 2022: ***Unstandardized estimate.***

Variables	Direct effect	Indirect effect	Total effect
**DV: Depression**	Estimate (95%CI)	Estimate(95% CI)	Estimate (95%CI)
Social support	-0.13[-0.229,-0.029]*	----	-0.13[-0.229,-0.029]*
Death of a loved one	0.03[0.004, 0.087]*	-----	0.03[0.004, 0.087]*
Self-rated academic ability	----	-0.05[-0.083, -0.031]*	-0.05[-0.083, -0.031]*
Physical trauma	----	0.10[0.037, 0.166]*	0.10[0.037, 0.166]*
Ever use of alcohol	0.06[-0.004, 0.112]	0.08[0.029, 0.139]*	0.14[0.073, 0.201]*
History of chronic illness	0.04[-0.053, 0.124]	0.17[0.085, 0.265]*	0.21[0.114, 0.311]*
Sex	-0.03[-0.099, 0.025]	0.09[0.042,0.162]*	0.06[0.003, 0.109]*
School type	-0.09[-0.194, -0.004]*	0.20[0.113, 0.320] *	0.11[0.016 , 0.207]*

Family pressure	0.02[-0.004, 0.040]	0.02[0.002, 0.043]*	0.04[0.016, 0.060]*
Stress	-0.06[-0,454, 0.247]	0.82[0.530, 1.250]*	0.76[0.642, 0.900]*
Anxiety	0.74[0.483, 1.081*]		0.74[0.483, 1.081*]

**Indicates significant variables, DV= dependent variable, SE= standard error, and CI=confidence interval*

Factors related to anxiety among high and preparatory school adolescents

Self-rated academic ability and physical trauma had a significant direct effect on anxiety. However, ever use of alcohol, and having a medically confirmed chronic illness had a significant indirect effect on anxiety. School type, sex, stress, and depression had statistically significant direct and indirect relationships with anxiety.

Depression [adjusted β= 0.74, 95% CI: 0.508, 1.010] and having a history of physical trauma [adjusted β= 0.06, 95% CI: 0.014, 0.125] had a positive direct effect on anxiety. Self-rated academic ability had a direct negative effect [adjusted β= -0.03, 95% CI: -0.065, -0.006] on anxiety. Perceived social support had a significant negative indirect effect [adjusted β= -0.22, 95% CI: -.304, -0.139] on anxiety. Stress had both direct [adjusted β= 0.54, 95% CI: 0.293, 0.745] and indirect [adjusted β= 0.57, 95% CI: 0.379, 0.814] positive effect on anxiety, bringing a total positive effect of 1.10 [adjusted β= 1.10, 95% CI: 0.955, 1.264] (***Table** 7*).

*Table 7: Direct, indirect, and total effect of socio-demographic, behavioral, relationship-related, and clinical factors on anxiety among adolescents in Northwest Ethiopia, 2022**: Unstandardized estimate.***

**Variables**	Direct effect	Indirect effect	Total effect
**DV: Anxiety**	Estimate (95% CI)	Estimate (95%CI)	Estimate (95% CI)
Self-rated academic	-0.03[-0.065, 0.006] *	----	-0.03[-0.065, 0.006]*

ability			
Physical trauma	0.06[0.014, 0.125]*	----	0.06[0.014, 0.125] *
Social support	----	-0.22[-0.304,-0.139] *	-0.22[-0.304,-0.139]*
Death of a loved one	----	0.06[0.010, 0.122] *	0.06[0.010, 0.122]*
Ever use of alcohol	-0.03[0.088, 0.035]	0.14[0.071, 0.213] *	0.11[0.041, 0.188] *
History of chronic illness	-0.02[-0.124, 0.078]	0.27[0.165, 0.384] *	0.25[0.132 , 0.371] *
Sex	0.08[0.019, 0.135] *	0.04[0.004, 0.092] *	0.12[0.059, 0.179] *
School type	0.12[0.017, 0.226] *	0.16[0.056, 0.269] *	0.28[0.159, 0.415] *
Family academic pressure	----	0.03[0.012, 0.050] *	0.03[0.001, 0.50] *
Stress	0.54[0.293, 0.745] *	0.57[0.379, 0.814] *	1.10[0.955, 1.264]*
Depression	0.74[0.508, 1.010] *	----	0.74[0.508, 1.010] *

**Indicates significant variables, DV= dependent variable, SE= standard error, and CI=confidence interval*

Factors related to somatic symptoms among high and preparatory school adolescents

Self-rated academic ability, perceived social support, physical trauma, and the death of a loved one in the past 6 months had a significant indirect effect on somatic symptoms. However, having a medically confirmed chronic illness, sex, stress, and depression were all directly and indirectly related to somatic symptoms.

Social support [adjusted β =-0.11, 95% CI: -0.176, -0.067] and self-rated academic ability [adjusted β =-0.02, 95% CI: -0.039, -010] had a significant negative indirect effect on somatic symptoms. Having medically confirmed chronic illness had significant direct [adjusted β= 0.17, 95% CI= 0.045, 0.290] and indirect [adjusted β= 0.20, 95% CI: 0.122, 0.289] effects on somatic symptoms, bringing a total positive effect of 0.37 [adjusted β= 0.37, 95% CI: 0.239, 0.505].

Stress had both direct [adjusted β= 0.53, 95% CI: 0.326, 0.768] and indirect [adjusted β= 0.33, 95% CI: 0.186, 0.472] positive effect on somatic symptoms resulting in a total positive effect of 0.86 [adjusted β= 086, 95% CI: 0.700, 1.025]. Depression had significant direct [adjusted β= 0.38, 95% CI= 0.167, 0.540] and indirect [adjusted β= 0.58, 95% CI: 0. 0.167, 3.629] positive effect on somatic symptoms resulting in a total positive effect of 0.96 [adjusted β= 0.96, 95% CI: 0.433, 1.456] (***Table** 8*).

*Table 8: Direct, indirect, and total effects of socio-demographic, personal, relationship-related factors, stress, anxiety, and depression on somatic symptom disorders among adolescents in Northwest Ethiopia, 2022**: Unstandardized estimate**.*

**Variables**	**Direct effect**	**Indirect effect**	**Total effect**
**DV: Somatic symptoms**	Estimate (95%)	Estimate (95%)	Estimate (95%)
Self-rated academic ability	----	-0.02[-0.039, -010]*	-0.02[-0.039, -010]*
Physical trauma	----	0.04[0.012, 0.083]*	0.04[0.012, 0.083]*
Social support	----	-0.11[-0.176, -0.067]*	-0.11[-0.176, -0.067]*
Death of a loved one within the past 6 months	----	0.03[0.005, 0.067]*	0.03[0.005, 0.067]*
Ever use of alcohol	0.03[-0.022, 0.105]	0.09[0.044, 0.144]*	0.12[0.062, 0.211]*
History of medically confirmed chronic illness	0.17[0.045, 0.290]*	0.20[0.122, 0.289]*	0.37[0.239 , 0.505]*
Sex	0.11[0.050, 0.166]*	0.03[0.002, 0.053]*	0.14[0.074, 0.198]*
School type	0.03[-0.074, 0.144]	0.13[0.053, 0.216]*	0.16[0.046, 0.292]*

Stress	0.53[0.326, 0.768]*	0.33[0.186, 0.472]*	0.86[0.700, 1.025]*
Depression	0.38[0.167, 0.540]*	0.58[0.167, 3.629]*	0.96[0.433, 3.774]*
Anxiety	0.06[-0.101, 0.212]	0.60[0.243, 3.825]*	0.66[0.270, 3.825]*

**Indicates significant variables, DV= dependent variable, SE= standard error, and CI=confidence interval*

Discussion

In this study, the prevalence and determinants of depression, anxiety, and somatic symptoms were examined using non-recursive structural equation modeling. [illegible]he prevalence rates of depression, anxiety, and somatic symptoms were [illegible]21% [95% CI: 25.8%, 31%], 35.82% [(95% CI: 33.3%, 38.4%), and 25.24% [95% CI: 23.0%, 27.6%] respectively. Sex, alcohol use, stress, self-rated academic ability, perceived social support, school type, and physical trauma were significant determinants of anxiety, depression, and somatic symptoms. The bidirectional relationship between anxiety and depression was significant. Depression and anxiety were significant determinants of somatic symptoms.

Prevalence of depression, anxiety, and somatic symptoms

In the current study, the overall prevalence of self-reported depression was 28.21% (95% CI= 25.8%, 31%). This is in line with the study conducted in Jimma 28% [17], but it was higher than the meta-analysis conducted in China (24.3%) [13], and lower than the studies conducted in Aksum (38.2%) [18], Nepal (44.2%) [99], Bangladesh (36%) [100], and India (57.7%) [101]. In this study, the prevalence of anxiety was 25.05% (95% CI: 22.8, 27.5%). This is consistent with the study conducted among children and youth in Ethiopia 0.5 to 23% [47]. However, it is lower than the study conducted in Kenya at 37.99% [16], Saudi Arabia at 63.5% to 66% [102, 103], Jordan at 42.1% [45], and Chandigarh at 80.85% [46]. In contrast, it was higher than the

worldwide estimates of anxiety among children and adolescents (6.5 to 10%). In our study, the prevalence of self-reported somatic symptom disorders was 25.24% (95% CI= 23, 27.6%). This is consistent with the study conducted among children and adolescents in Tarragona which reported 5 to 30% [62]. However, it was higher than the prevalence in the general population (5–7%) [104] and lower than that in a study conducted in Qatar (47.8% in females and 52.2% in males) [105]. This disparity [illegible]uld be explained by differences in screening tools and cutoff points [18, 46, 106]. Furthermore, since mental health is the combination of physical, social, cultural, and religious environments this could be explained by socio-cultural differences in different settings. Poverty, violence, and other stressful social conditions are not unique to any one region of the world, nor are the symptoms and manifestations that result from them. However, factors that are frequently associated with race or ethnicity, such as socioeconomic position or country of origin, can enhance the likelihood of being exposed to these sorts of stressors, and thus stressors may lead to depression, anxiety, and somatic symptoms [107]. Additionally, [illegible]ernal conflict in northern Ethiopia may raise the frequency of depression, anxiety, and somatic symptoms [81].

Factors related to depression, anxiety, and somatic symptoms

Female sex had a statistically significant positive effect on depression [adjusted β= 0.06, 95% CI: 0.003, 0.109], anxiety [adjusted β= 0.12, 95% CI: 0.059, 0.179], and somatic symptoms [adjusted β=0.13, 95% CI: 0.074, 0.198]; this implies that being female increases the levels of depression, anxiety, and somatic symptoms compared to being male, controlling for other factors. This finding is consistent with other studies [17, 19-21]. This could be explained by the fact that men and women have different brain chemistry and hormonal variations. Menstrual fluctuations in progesterone and estrogen may affect neurotransmitters in women, and low

neurotransmitters may lead to depression, anxiety, and somatic symptoms. Moreover, this might be due to the existence of androgen receptors in males, which may provide protection [108]. It could also be that females are more likely to report on their mental illness. Cultures differ in how men and women deal with issues such as depression, which may influence how open or unwilling men and women are to reveal being depressed or having other mental health disorders [65, 66, 109-111].

Ever use of alcohol had a positive effect on depression [β= 0.14, 95% CI: 0.073, 0.201], anxiety [adjusted β= 0.11, 95% CI: 0.041, 0.188], and somatic symptoms [adjusted β= 0.12, 95% CI: 0.062, 0.211]. This implied that adolescents who had a history of alcohol drinking had higher levels of depression, anxiety, and somatic symptoms than those who did not drink. This finding is congruent with the study conducted in the USA [27] and in China [28, 112]. This could be because alcohol affects brain chemicals such as serotonin and dopamine, which are responsible for happiness, and the decrease in these chemicals leads to feelings of depression [113]. Furthermore, alcohol consumption may contribute to sadness, anxiety, and somatic symptoms in adolescents by reducing age-appropriate activities such as physical activity and social relationships [114]. Moreover, it could be due to the direct effect of ethanol on nerve cells [115] which leads to heightened awareness of normal body sensations.

Stress had a significant positive effect on depression [adjusted β= 0.76, 95% CI: 0.642, 0.900], anxiety [adjusted β= 1.10, 95% CI: 0.955, 1.264], and somatic symptoms [adjusted β= 086, 95% C: 0.700, 1.025]. This indicates that participants with high levels of stress had higher levels of depression, anxiety, and somatic symptoms, holding other predictors constant. Our finding is supported by different studies [31, 32, 71]. The possible justification for this could be that high-level stress impairs brain function, which may lead to fluctuations in neurotransmitters

such as dopamine, which leads to depression. In addition, immune dysregulation during stressful life events leads to anxiety, and anxiety leads to depression and depression may lead to somatic symptoms.

As the self-rated academic ability of adolescents increases, the levels of depression [adjusted β=-0.05, 95% CI: -0.083,-0.031], anxiety [adjusted β= -0.03, 95% CI: -0.065, -0.006], and somatic symptoms [adjusted β =-0.02, 95% CI: -0.039, -010] decrease. This result is compatible with the study conducted in Iran [116], and Slovakia [117] and with other studies [45, 51, 118]. The possible explanation for this might be, that students with low academic ability are terrified of negative responses from teachers, parents, and friends, which may lead to fear about their future career, which will lead them to become anxious, and anxiety will lead to depression and somatic symptoms.

Perceived social support had a significant direct negative effect [adjusted β= -0.13, 95% CI: -0.229,-0.029] on depression. This means that as social support increased, the level of depression decreased or students with low social support had higher levels of depression than those who had high social support. This finding is compatible with different reports [25, 26, 34, 38]. The probable reason for this might be that low social support may increase feelings of loneliness, worthlessness, and hopelessness, leading to a loss of interest in activities and depression. Moreover, students with low social support are more likely to have low self-esteem and negative cognition than those with higher social support, which may lead to depression [119].

Learning in a private school had a statistically significant positive effect [adjusted β= 0.10, 95% CI: 0.016, 0.207] on depression. This means that participants in private schools had

higher levels of depression than their counterparts. In contrast to our finding, the study conducted in India [22] showed that public school students had a higher level of depression than private school students; this disparity may be explained by differences in sample size and method of analysis. Our study had a larger sample size than the India study, and the India study did not address the confounding effect. Higher levels of depression in private schools may be due to parents' expectations of high academic achievement in private schools; which leads students to be stressed when they try to meet their parents' expectations, and stress leads to depression.

Our findings showed that holding other predictors constant, having a history of physical trauma had a direct positive effect [adjusted β= 0.06, 95% CI: 0.014, 0.125] on anxiety. This indicates that students with physical trauma had higher levels of anxiety than those who did not. Our finding was supported by the study conducted in the Netherlands [120]. This consistency could be justified by the fact that in reaction to trauma, neurotransmitters such as dopamine, serotonin, and gamma amino butyric acid may be impacted by cortisol and depletion of these neurotransmitters may lead to anxiety. In addition, individuals with physical trauma may internalize the event and may have subsequent negative thoughts about themselves, which affect their emotional experience and might lead to anxiety [121].

[illegible]s the level of depression increases the level of somatic symptoms will also increase [adjusted β= 0.96, 95% CI= 0.167, 3.629]. In the same way, as the level of anxiety increases the level of somatic symptoms also increases [adjusted β=0.66, 95% CI= 0.270, 3.825]. This result is supported by other studies [66, 76, 77] [66, 74, 75]. A possible explanation for this consistency could be that in depressed individuals’ neurotransmitters such as nor-epinephrine and serotonin are low; a decrease in neurotransmitters may lead individuals to focus on physical symptoms. Moreover, it could be explained by the co-occurrence of somatic symptoms, anxiety, and

depression. The sharing of common sets of gene and environmental factors may also be a possible justification.

Strengths and limitations

A bidirectional relationship between anxiety and depression was assessed. In addition, the direct and indirect effects of different independent variables on different dependent variables were assessed simultaneously. However, this study is not without limitations. First, only school adolescents in Northwest Ethiopia were included in the study which may affect generalization to all adolescents in Ethiopia. Second, self-reported screening tools were used, which may overestimate the effect size. Third, we only asked about alcohol intake once, not how much, how frequently, or for how long, which could influence the outcome. Last, the temporal relationship between predictors and outcomes was not evaluated due to the cross-sectional nature of the data.

Conclusion

In this study, a moderate prevalence of depression, anxiety, and somatic symptoms was found. Self-rated academic ability, physical trauma, school type, sex, stress, ever use of alcohol, perceived social support, death of a loved one, and having medically confirmed chronic illness were significant determinants of depression, anxiety, and somatic symptoms. The bidirectional relationship between anxiety and depression was significant. Depression and anxiety were significant determinants of somatic symptoms. Based on the study findings, the authors suggest the following points: Policymakers should strengthen the prevention and management of adolescent mental health disorders. It is better to initiate and implement school-based mental health screening services, particularly for female students and students with low academic ability by educational offices and schools. It is important to emphasize the two-way relationship

between anxiety and depression, and somatic symptoms should be suspected in individuals with depression and anxiety. Studies including out-of-school adolescents are recommended and it is better to assess temporal relationships by using follow-up studies.

Abbreviations

AIC: Akaike information Criteria, OVB: Omitted variable bias, CFI: Comparative Fix index, CMB: Common Method Bias, OVB: Omitted Variable Bias, PHQ-9A: Patent Health Questionnaire for Adolescent, RMSEA: Rooted Mean Square Error of Approximation, TLI: Tucker Lewis Index,

Acknowledgment

The authors are grateful to the University of Gondar, data collectors, supervisors, and study participants.

Authors' contribution

Conceptualization: Zenebe Abebe Gebreegziabher

Data curation: Zenebe Abebe Gebreegziabher. Ayenew Molla, Rediet Eristu

Formal analysis: Zenebe Abebe Gebreegziabher

Investigation: Zenebe Abebe Gebreegziabher. Ayenew Molla, Rediet Eristu

Methodology: Zenebe Abebe Gebreegziabher. Ayenew Molla, Rediet Eristu

Project administration: Zenebe Abebe Gebreegziabher. Ayenew Molla, Rediet Eristu

Resource: Zenebe Abebe Gebreegziabher. Ayenew Molla, Rediet Eristu

Software: Zenebe Abebe Gebreegziabher, Ayenew Molla, Rediet Eristu

Supervision: Zenebe Abebe Gebreegziabher, Ayenew Molla, Rediet Eristu

Validation: Zenebe Abebe Gebreegziabher, Ayenew Molla, Rediet Eristu

Visualization: Zenebe Abebe Gebreegziabher

Writing: original draft: Zenebe Abebe Gebreegziabher

Writing: review and editing: Zenebe Abebe Gebreegziabher. Ayenew Molla, Rediet Eristu

Supporting Information

S1 Table 1: Summary of key variables in the study

S1 Table 2: Intraclass correlation coefficients for each outcome variable

S1 Table 3: Instrumental variable validity and strength

S1 Table 4: Depression among adolescents in Gondar town, 2022

S1 Table 5: Anxiety among adolescents in Gondar town, 2022

S1 Table 6: Somatic symptoms among adolescents in Gondar town, 2022

S1 Table 7**:** Factorial average item parceling for depression, somatic symptoms, and their determinants among adolescents in Gondar town, 2022

S2 Fig 1: Hypothetical measurement model for depression, somatic symptoms, and their determinants among adolescents in Gondar town, 2022

S3 annex 1: Results of the pilot study

S4 Table: Tools to assess anxiety, depression, somatic symptoms, and their determinants among adolescents in Gondar town, 2022

S5 Data set: Data set of adolescents in Gondar town, 2022

References

1. World Health Organization, *The global burden of disease: 2004 update*. 2008: World Health Organization.
2. Organization, W.H., *Mental health status of adolescents in South-East Asia: Evidence for action.* 2017.
3. Jörns-Presentati, A., et al., *The prevalence of mental health problems in sub-Saharan adolescents: A systematic review.* Plos one, 2021. **16**(5): p. e0251689.
4. Armocida, B., et al., *Burden of non-communicable diseases among adolescents aged 10–24 years in the EU, 1990–2019: a systematic analysis of the Global Burden of Diseases Study 2019.* The Lancet Child & Adolescent Health, 2022.
5. WHO, *Adolescents mental health.* 2019.
6. Ryan, G., O. Qureshi, and N. Salaria, *Mental health and the 2030 Sustainable Development Agenda: Global inaction on mental health is putting the brakes on development.* London, UK: London School of Hygiene and Tropical Medicine, 2018.
7. Kassebaum, N.J., et al., *Global, regional, and national disability-adjusted life-years (DALYs) for 315 diseases and injuries and healthy life expectancy (HALE), 1990–2015: a systematic analysis for the Global Burden of Disease Study 2015*. The Lancet, 2016. **388**(10053): p. 1603-1658.
8. Sayers, J., *The World Health Report 2001-Mental health: new understanding, new hope.* Bulletin of the World Health Organization, 2001. **79**: p. 1085-1085.
9. Organization, W.H., *Depression and other common mental disorders: global health estimates*. 2017, World Health Organization.

10. Perez, D.L., et al., *A neural circuit framework for somatosensory amplification in somatoform disorders.* J Neuropsychiatry Clin Neurosci, 2015. **27**(1): p. e40-50.

11. WHO. *depression definition* 2019; Available from: https://www.who.int/news-room/fact-sheets/detail/adolescent-mental-health.

12. WHO., *Adolescents: health risks and solutions. Geneva: World Health Organization.* 2018.

13. Tang, X., et al., *Prevalence of depressive symptoms among adolescents in secondary school in mainland China: A systematic review and meta-analysis.* J Affect Disord, 2019. **245**: p. 498-507.

14. El-Missiry, A., et al., *Screening for depression in a sample of Egyptian secondary school female students.* J Affect Disord, 2012. **136**(1-2): p. e61-e68.

15. Erskine, H.E., et al., *The global coverage of prevalence data for mental disorders in children and adolescents.* Epidemiol Psychiatr Sci, 2017. **26**(4): p. 395-402.

16. Osborn, T.L., et al., *Depression and Anxiety Symptoms, Social Support, and Demographic Factors Among Kenyan High School Students.* Journal of Child and Family Studies, 2020. **29**(5): p. 1432-1443.

17. Girma, S., et al., *Depression and its determinants among adolescents in Jimma town, Southwest Ethiopia.* PLoS One, 2021. **16**(5): p. e0250927.

18. Tirfeneh, E. and M. Srahbzu, *Depression and its association with parental neglect among adolescents at governmental high schools of Aksum town, Tigray, Ethiopia, 2019: a cross-sectional study*. Depression research and treatment, 2020. **2020**.

19. Trivedi, D., et al., *Depression among adolescent students in South India: How serious is the issue?* Indian Journal of Psychiatry, 2016. **58**(3): p. 349-350.

20. Raheel, H., *Depression and associated factors among adolescent females in Riyadh, Kingdom of Saudi Arabia, a cross-sectional study.* International journal of preventive medicine, 2015. **6**.

21. Rehm, J. and K.D. Shield, *Global burden of disease and the impact of mental and addictive disorders.* Current Psychiatry Reports, 2019. **21**(2): p. 1-7.

22. Kumar, R.K., et al., *The prevalence of depression, anxiety, and stress among high school adolescent's children in public and private schools in Rangareddy district Telangana state: A cross-sectional study.* Journal of Education and Health Promotion, 2022. **11**(1): p. 83.

23. BP, C., *Stress, anxiety, and depression among adolescent students of public schools in Kathmandu.* Journal of Institute of Medicine, 2018. **40**(3).

24. Nguyen, D.T., et al., *Depression, anxiety, and suicidal ideation among Vietnamese secondary school students and proposed solutions: a cross-sectional study.* BMC Public Health, 2013. **13**(1): p. 1-10.

25. Addy, N.D., et al., *Mental health difficulties, coping mechanisms and support systems among school-going adolescents in Ghana: A mixed-methods study.* PLoS One, 2021. **16**(4): p. e0250424.

26. Deb, S., E. Strodl, and H. Sun, *Academic stress, parental pressure, anxiety, and mental health among Indian high school students.* International Journal of Psychology and Behavioral Science, 2015. **5**(1): p. 26-34.

27. Galaif, E.R., et al., *Suicidality, depression, and alcohol use among adolescents: a review of empirical findings.* International journal of adolescent medicine and health, 2007. **19**(1): p. 27-36.

28. Huang, R., et al., *Reported alcohol drinking and mental health problems in Hong Kong Chinese adolescents.* Drug and alcohol dependence, 2016. **164**: p. 47-54.

29. Shah, S.M., et al., *Self-esteem and other risk factors for depressive symptoms among adolescents in the United Arab Emirates.* PloS one, 2020. **15**(1): p. e0227483.

30. Gülsen, A. and B. Uygur, *Psychological Features of Smokers.* Respir Care, 2018. **63**(12): p. 1492-1497.

31. Ibrahim, N., et al., *Prevalence and predictors of depression and suicidal ideation among adolescents attending government secondary schools in Malaysia.* Medical Journal of Malaysia, 2017. **72**(4): p. 221-227.

32. Ghobadzadeh, M., et al., *Relationships between adolescent stress, depressive symptoms, and sexual risk behavior in young adulthood: A structural equation modeling analysis.* Journal of Pediatric Health Care, 2019. **33**(4): p. 394-403.

33. Lindholdt, L., et al., *Perceived stress among adolescents as a marker for future mental disorders: A prospective cohort study.* Scandinavian Journal of Public Health, 2021: p. 1403494821993719.

34. Cao, C., et al., *Anxiety, depression, and PTSD symptoms among high school students in china in response to the COVID-19 pandemic and lockdown.* Journal of Affective Disorders, 2022. **296**: p. 126-129.

35. AlAzzam, M., et al., *Predictors of depression and anxiety among senior high school students during COVID-19 pandemic: The context of home quarantine and online education.* The Journal of School Nursing, 2021. **37**(4): p. 241-248.

36. Wittchen, H.-U., C.A. Essau, and J.-C. Krieg, *Anxiety disorders: similarities and differences of comorbidity in treated and untreated groups.* The British Journal of Psychiatry, 1991. **159**(S12): p. 23-33.

37. Essau, C.A., J. Conradt, and F. Petermann, *Frequency, comorbidity, and psychosocial impairment of anxiety disorders in German adolescents.* Journal of anxiety disorders, 2000. **14**(3): p. 263-279.

38. Chang, C.-W., R. Yuan, and J.-K. Chen, *Social support and depression among Chinese adolescents: The mediating roles of self-esteem and self-efficacy.* Children and Youth Services Review, 2018. **88**: p. 128-134.

39. Bandelow, B. and S. Michaelis, *Epidemiology of anxiety disorders in the 21st century.* Dialogues in clinical neuroscience, 2022.

40. Beesdo, K., et al., *Incidence and risk patterns of anxiety and depressive disorders and categorization of generalized anxiety disorder.* Archives of General Psychiatry, 2010. **67**(1): p. 47-57.

41. Yang, X., et al., *Global, regional and national burden of anxiety disorders from 1990 to 2019: results from the Global Burden of Disease Study 2019.* Epidemiology and Psychiatric Sciences, 2021. **30**: p. e36.

42. Vos, T., et al., *Global burden of 369 diseases and injuries in 204 countries and territories, 1990–2019: a systematic analysis for the Global Burden of Disease Study 2019.* The Lancet, 2020. **396**(10258): p. 1204-1222.

43. Kessler, R.C., et al., *Twelve‐ month and lifetime prevalence and lifetime morbid risk of anxiety and mood disorders in the United States.* International journal of methods in psychiatric research, 2012. **21**(3): p. 169-184.

44. Biswas, T., et al., *Global variation in the prevalence of suicidal ideation, anxiety, and their correlates among adolescents: a population-based study of 82 countries.* EClinicalMedicine, 2020. **24**: p. 100395.

45. Malak, M.Z. and A.H. Khalifeh, *Anxiety and depression among school students in Jordan: Prevalence, risk factors, and predictors.* Perspectives in psychiatric care, 2018. **54**(2): p. 242-250.

46. Sandal, R.K., et al., *Prevalence of depression, anxiety and stress among school going adolescent in Chandigarh.* Journal of family medicine and primary care, 2017. **6**(2): p. 405.

47. Bifftu, B. and Y. Guracho, *A Systematic Review and Meta-Analysis of Anxiety among Children and Youth in Ethiopia.* J Depress Anxiety Disorder, 2021. **3**(2): p. 89-100.

48. Lancet, T., *Better understanding of youth mental health*. 2017. p. 1670.

49. Tramonte, L. and D. Willms, *The prevalence of anxiety among middle and secondary school students in Canada.* Canadian Journal of Public Health/Revue Canadienne de Sante'e Publique, 2010: p. S19-S22.

50. Smith, L., et al., *Correlates of symptoms of anxiety and depression and mental wellbeing associated with COVID-19: a cross-sectional study of UK-based respondents.* Psychiatry Res, 2020. **291**: p. 113138.

51. Syokwaa, K.A., P.J. Aloka, and S.N.F. Ndunge, *The relationship between anxiety levels and academic achievement among students in selected secondary schools in Lang'ata District, Kenya.* Journal of Educational and Social Research, 2014. **4**(3): p. 403-403.

52. Khanum, F. and S. Razack, *Anxiety–Herbal treatment: A review.* Res Rev Biomed Biotech, 2010. **1**(2): p. 83-89.

53. Kumari, A. and J. Jain, *Examination stress and anxiety: A study of college students.* Global Journal of Multidisciplinary Studies, 2014. **4**(1): p. 31-40.

54. Bekhuis, E., et al., *Differential associations of specific depressive and anxiety disorders with somatic symptoms.* Journal of Psychosomatic Research, 2015. **78**(2): p. 116-122.

55. Rice, F., M.B.M. van den Bree, and A. Thapar, *A population-based study of anxiety as a precursor for depression in childhood and adolescence.* BMC Psychiatry, 2004. **4**(1): p. 43.

56. Eriksen, T.E. and M.B. Risør, *What is called symptom?* Med Health Care Philos, 2014. **17**(1): p. 89-102.

57. Edgcomb, J.B., C.H. Tseng, and B. Kerner, *Medically unexplained somatic symptoms and bipolar spectrum disorders: A systematic review and meta-analysis.* J Affect Disord, 2016. **204**: p. 205-13.

58. Tomenson, B., et al., *Total somatic symptom score as a predictor of health outcome in somatic symptom disorders.* Br J Psychiatry, 2013. **203**(5): p. 373-80.

59. Löwe, B., et al., *Depression, anxiety and somatization in primary care: syndrome overlap and functional impairment.* General hospital psychiatry, 2008. **30**(3): p. 191-199.

60. Kroenke, K. and A.D. Mangelsdorff, *Common symptoms in ambulatory care: Incidence, evaluation, therapy, and outcome.* The American Journal of Medicine, 1989. **86**(3): p. 262-266.

61. Kurlansik, S.L. and M.S. Maffei, *Somatic symptom disorder.* American family physician, 2016. **93**(1): p. 49-54.

62. Martin, J.C.S., et al., *Somatic complaints and symptoms of anxiety and depression in a school-based sample of preadolescents and early adolescents. Functional impairment*

and implications for treatment. Journal of Cognitive and Behavioral Psychotherapies, 2011. **11**(2): p. 191-208.

63. Carlsson, J., *Prevalence of headache in schoolchildren: relation to family and school factors.* Acta Paediatr, 1996. **85**(6): p. 692-6.

64. Murray, A.M., et al., *Barriers to the diagnosis of somatoform disorders in primary care: protocol for a systematic review of the current status.* Systematic reviews, 2013. **2**(1): p. 1-8.

65. Karaca, S., et al., *Somatic Symptoms in Secondary School Students and Parental Attitudes.* Journal of Psychiatric Nursing/Psikiyatri Hemsireleri Dernegi, 2015. **6**(3).

66. Haraldstad, K. and T.H. Stea, *Associations between pain, self-efficacy, sleep duration, and symptoms of depression in adolescents: a cross-sectional survey.* BMC Public Health, 2021. **21**(1): p. 1-7.

67. Larsson, B.S., *Somatic complaints and their relationship to depressive symptoms in Swedish adolescents.* Journal of Child Psychology and Psychiatry, 1991. **32**(5): p. 821-832.

68. Heidari, Z., et al., *Demographic and lifestyles Determinants of Somatic Complaints' Structures: A Cross-sectional Study on a Large Sample of Iranian Adults Using Factor Mixture Model.* Int J Prev Med, 2017. **8**: p. 8.

69. Ruotsalainen, H., et al., *Systematic review of physical activity and exercise interventions on body mass indices, subsequent physical activity, and psychological symptoms in overweight and obese adolescents.* Journal of Advanced Nursing, 2015. **71**(11): p. 2461-2477.

70. Noh, J.-W., et al., *Relationships between Extra-School Tutoring Time, Somatic Symptoms, and Sleep Duration of Adolescent Students: A Panel Analysis Using Data from the Korean Children and Youth Panel Survey*. International Journal of Environmental Research and Public Health, 2020. **17**(21): p. 8037.

71. Glise, K., G. Ahlborg, and I.H. Jonsdottir, *Prevalence and course of somatic symptoms in patients with stress-related exhaustion: does sex or age matter*. BMC Psychiatry, 2014. **14**(1): p. 1-13.

72. Garralda, M.E., *Unexplained physical complaints*. Pediatric Clinics, 2011. **58**(4): p. 803-813.

73. Glise, K., G. Ahlborg, Jr., and I.H. Jonsdottir, *Prevalence and course of somatic symptoms in patients with stress-related exhaustion: does sex or age matter*. BMC Psychiatry, 2014. **14**: pp. 118-118.

74. van Boven, K., et al., *Do unexplained symptoms predict anxiety or depression? Ten-year data from a practice-based research network*. British Journal of General Practice, 2011. **61**(587): p. e316-e325.

75. Kingery, J.N., G.S. Ginsburg, and C.A. Alfano, *Somatic symptoms and anxiety among African American adolescents*. Journal of Black Psychology, 2007. **33**(4): p. 363-378.

76. Janssens, K.A., et al., *Anxiety and depression are risk factors rather than consequences of functional somatic symptoms in a general population of adolescents: the TRAILS study*. Journal of Child Psychology and Psychiatry, 2010. **51**(3): p. 304-312.

77. Knook, L.M., et al., *Psychiatric disorders in children and adolescents presenting with unexplained chronic pain: what is the prevalence and clinical relevancy?* European child & adolescent psychiatry, 2011. **20**(1): p. 39-48.

78. Abdulghani, H.M., et al., *Stress and its effects on medical students: a cross-sectional study at a college of medicine in Saudi Arabia.* Journal of health, population, and nutrition, 2011. **29**(5): p. 516.

79. Organization, W.H., *World health statistics overview 2019: Monitoring health for the SDGs, sustainable development goals*. 2019, World Health Organization.

80. Erskine, H., et al., *The global coverage of prevalence data for mental disorders in children and adolescents.* Epidemiology and psychiatric sciences, 2017. **26**(4): p. 395-402.

81. Dadi, A.F., *The mental health consequences of war in northern Ethiopia: why we should be concerned.* The Lancet Psychiatry, 2022. **9**(3): p. 194-195.

82. report, G.c.a., *total population estimation report.* Gondar City Administrative Office, 2022.

83. Bentler, P.M. and C.-P. Chou, *Practical issues in structural modeling.* Sociological methods & research, 1987. **16**(1): p. 78-117.

84. Kroenke, K., R.L. Spitzer, and J.B. Williams, *The PHQ‐9: validity of a brief depression severity measure.* Journal of General Internal Medicine, 2001. **16**(9): p. 606-613.

85. Spitzer, R.L., et al., *A brief measure for assessing generalized anxiety disorder: the GAD-7.* Archives of internal medicine, 2006. **166**(10): p. 1092-1097.

86. Gierk, B., et al., *The somatic symptom scale–8 (SSS-8): a brief measure of somatic symptom burden.* JAMA Internal Medicine, 2014. **174**(3): p. 399-407.

87. Cohen, S., T. Kamarck, and R. Mermelstein, *A global measure of perceived stress.* Journal of Health and Social Behavior, 1983: p. 385-396.

88. Bøen, H., O.S. Dalgard, and E. Bjertness, *The importance of social support in the associations between psychological distress and somatic health problems and socio-economic factors among older adults living at home: a cross-sectional study.* BMC geriatrics, 2012. **12**(1): p. 1-12.
89. Group, W.A.W., *The alcohol, smoking and substance involvement screening test (ASSIST): development, reliability, and feasibility.* Addiction, 2002. **97**(9): p. 1183-1194.
90. Biddle, S.J., et al., *An assessment of self-reported physical activity instruments in young people for population surveillance: Project ALPHA.* International Journal of Behavioral Nutrition and Physical Activity, 2011. **8**(1): p. 1-9.
91. Shukur, G. and P. Mantalos, *Size and power of the RESET test as applied to systems of equations: a bootstrap approach.* Journal of Modern Applied Statistical Methods, 2004. **3**(2): p. 10.
92. Kline, R.B., *Principles and practice of structural equation modeling*. 2015: Guilford publications.
93. Kumar, S. and S. Kumar, *Structure equation modeling basic assumptions and concepts: A novices guide.* Asian Journal of Management Sciences, 2015. **3**(07): p. 25-28.
94. Roth, P.L. and F.S. Switzer III, *A Monte Carlo analysis of missing data techniques in an HRM setting.* Journal of Management, 1995. **21**(5): p. 1003-1023.
95. Aguirre-Urreta, M.I. and J. Hu, *Detecting common method bias: Performance of Harman's single-factor test.* ACM SIGMIS Database: The Database for Advances in Information Systems, 2019. **50**(2): p. 45-70.
96. Paxton, P., et al., *Nonrecursive models: Endogeneity, reciprocal relationships, and feedback loops*. Vol. 168. 2011: Sage Publications.

97. Technology, E.F.M.o.S.a., *national-research-ethics-review-guidline*. 2014.

98. Steiger, J.H., *Structural model evaluation and modification: An interval estimation approach.* Multivariate behavioral research, 1990. **25**(2): p. 173-180.

99. Bhattarai, D., N. Shrestha, and S. Paudel, *Prevalence and factors associated with depression among higher secondary school adolescents of Pokhara Metropolitan, Nepal: a cross-sectional study.* BMJ Open, 2020. **10**(12): p. e044042.

100. Anjum, A., et al., *Investigating the prevalence of and factors associated with depressive symptoms among urban and semi-urban school adolescents in Bangladesh: a pilot study.* International Health, 2019.

101. Urmila, K., et al., *Prevalence and risk factors associated with depression among higher secondary school students residing in a boarding school of North Kerala, India.* Int J Contemp Pediatr, 2017. **4**(3): p. 735.

102. Khalid S, A.G., A.A. Hasan S, and M. Ossama A, *Prevalence of depression, anxiety and stress as measured by the depression, anxiety, and stress scale [DASS-42] among secondary school girls in Abha, Saudi Arabia.* 2009.

103. Alharbi, R., et al., *Depression and anxiety among high school student at Qassim Region.* Journal of family medicine and primary care, 2019. **8**(2): p. 504.

104. Widiger, T.A., P.T. Costa, and A.P. Association, *Personality disorders and the five-factor model of personality*. 2013: JSTOR.

105. Bender, A., S. Ghuloum, and F.T. Burgut, *Gender differences in the prevalence of somatoform disorders in patients visiting primary care centers.* Journal of primary care & community health, 2010. **1**(1): p. 37-42.

106. Anjum, A., et al., *Investigating the prevalence of and factors associated with depressive symptoms among urban and semi-urban school adolescents in Bangladesh: a pilot study.* International health, 2022. **14**(4): p. 354-362.

107. Satcher, D., *Mental health: Culture, race, and ethnicity—A supplement to mental health: A report of the surgeon general*. 2001: US Department of Health and Human Services.

108. McEwen, B.S. and T.A. Milner, *Hippocampal formation: shedding light on the influence of sex and stress on the brain.* Brain Res Rev, 2007. **55**(2): p. 343-55.

109. Johnson, D.P. and M.A. Whisman, *Gender differences in rumination: A meta-analysis.* Personality and individual differences, 2013. **55**(4): p. 367-374.

110. Wharton, W., et al., *Neurobiological underpinnings of the estrogen-mood relationship.* Current Psychiatry Reviews, 2012. **8**(3): p. 247-256.

111. Narbonne, M., et al., *Hormonal dysregulation and catamenial epilepsy.* Functional Neurology, 1990. **5**(1): p. 49-53.

112. Huang, R., et al., *Reported alcohol drinking and mental health problems in Hong Kong Chinese adolescents.* Drug and Alcohol Dependence, 2016. **164**: p. 47-54.

113. Deykin, E.Y., J.C. Levy, and V. Wells, *Adolescent depression, alcohol and drug abuse.* American Journal of Public Health, 1987. **77**(2): p. 178-182.

114. Miller, J.W., et al., *Binge drinking and associated health risk behaviors among high school students.* Pediatrics, 2007. **119**(1): p. 76-85.

115. Guerri, C. and M. Pascual, *Mechanisms involved in the neurotoxic, cognitive, and neurobehavioral effects of alcohol consumption during adolescence.* Alcohol, 2010. **44**(1): p. 15-26.

116. Yousefi, F., et al., *The relationship between gender, age, depression and academic achievement.* 2010.

117. Demuthova, S., *The School Environment as a Source of Somatic Problems in Adolescents.* Revista Românească pentru Educaţie Multidimensională, 2019. **11**(2): p. 59-71.

118. El-Anzi, F.O., *Academic achievement and its relationship with anxiety, self-esteem, optimism, and pessimism in Kuwaiti students.* Social Behavior and Personality: An international journal, 2005. **33**(1): p. 95-104.

119. Li, Y., et al., *Social support, attachment closeness, and self-esteem affect depression in international students in China.* Frontiers in Psychology, 2021. **12**: p. 618105.

120. Hovens, J.G., et al., *Childhood life events and childhood trauma in adult patients with depressive, anxiety and comorbid disorders vs. controls.* Acta Psychiatrica Scandinavica, 2010. **122**(1): p. 66-74.

121. Association, A.P., *DSM 5 Diagnostic and statistical manual of mental disorders*, in *DSM 5 Diagnostic and statistical manual of mental disorders*. 2013. p. 947 p.-947 p.

Click here to access/download;Figure;fig 1 specification.tif

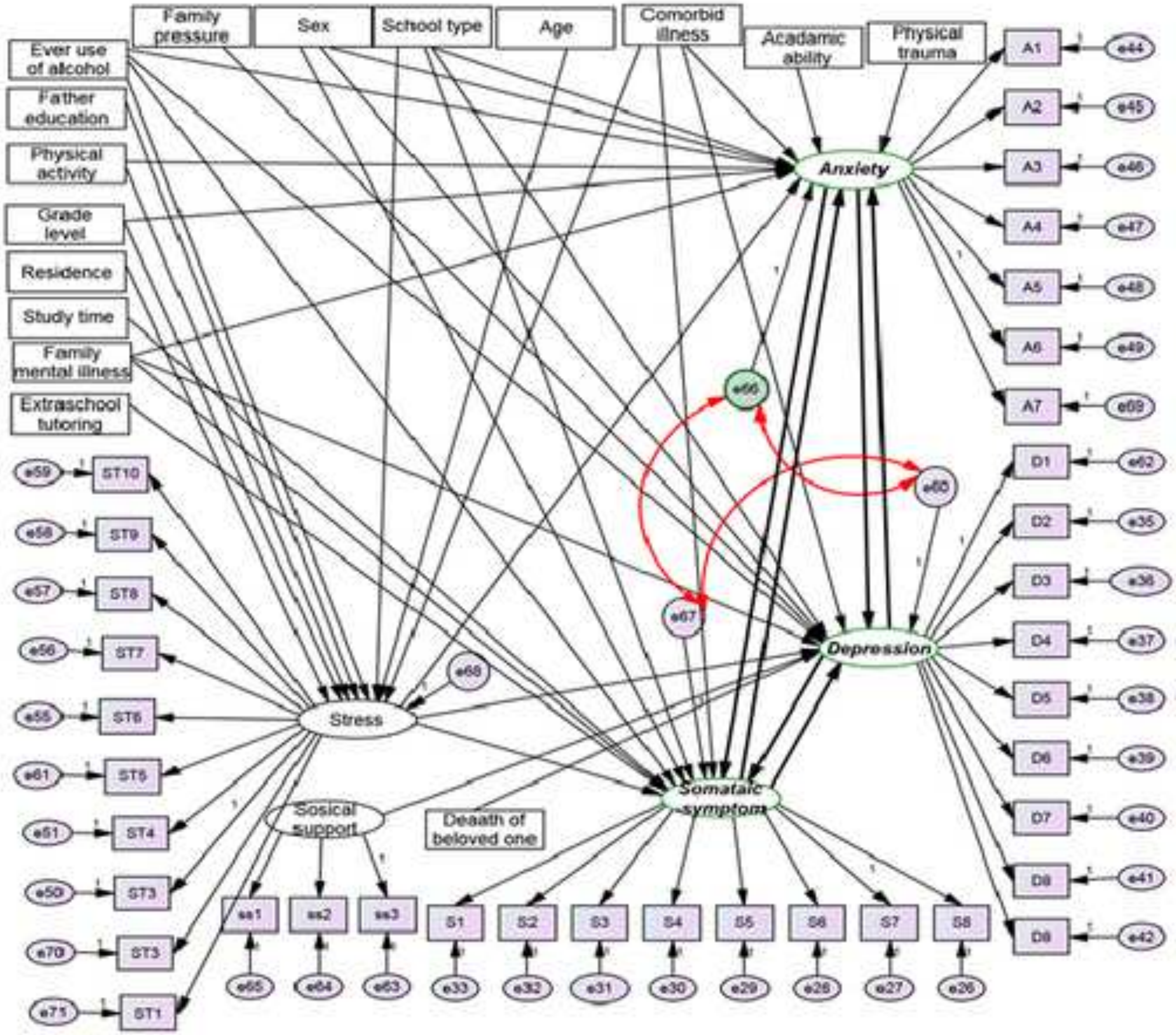

Click here to access/download;Figure;fig 3 final measurment model.tif

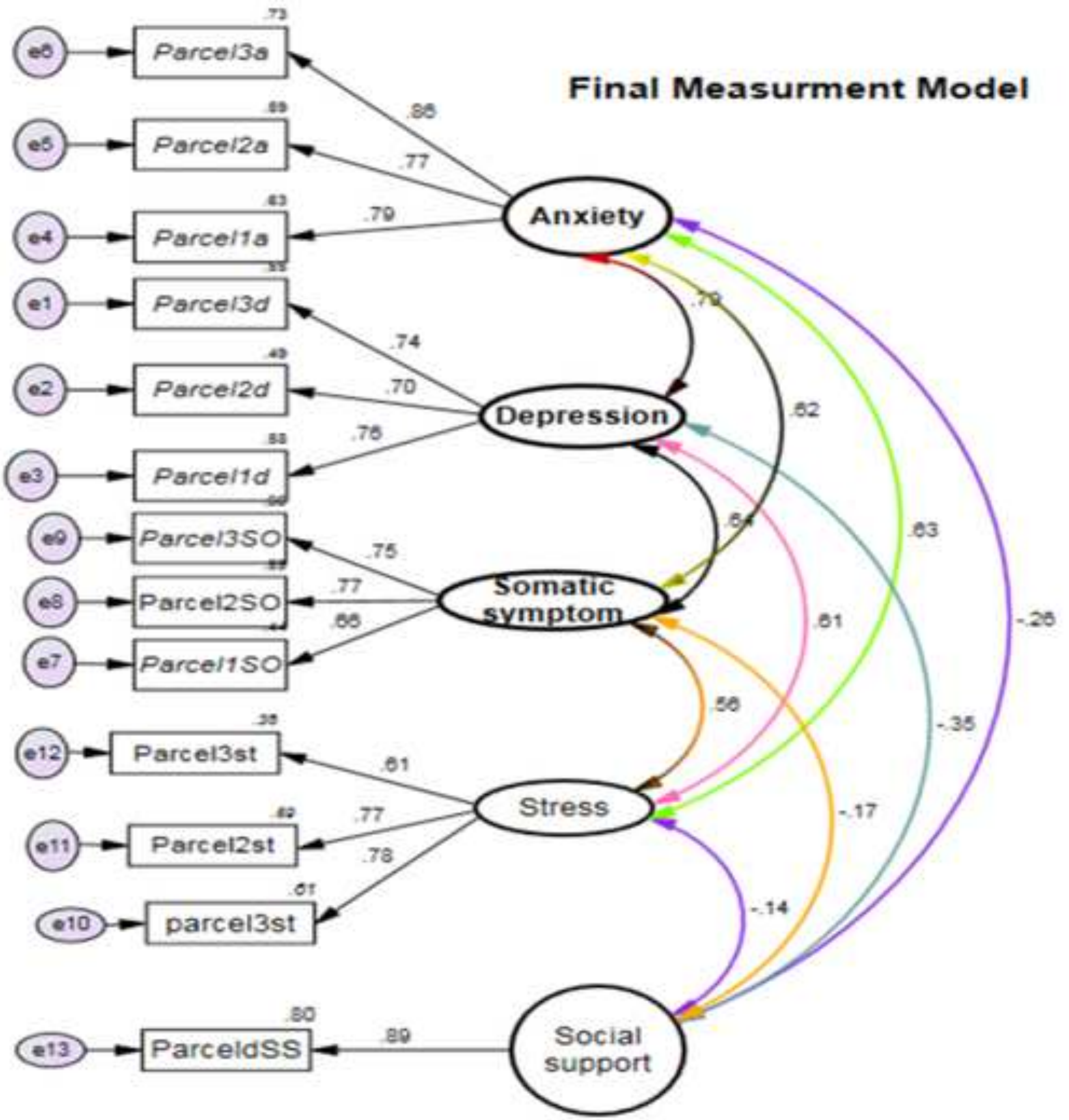

Click here to access/download;Figure;Fig 2 sampling procedure.tif

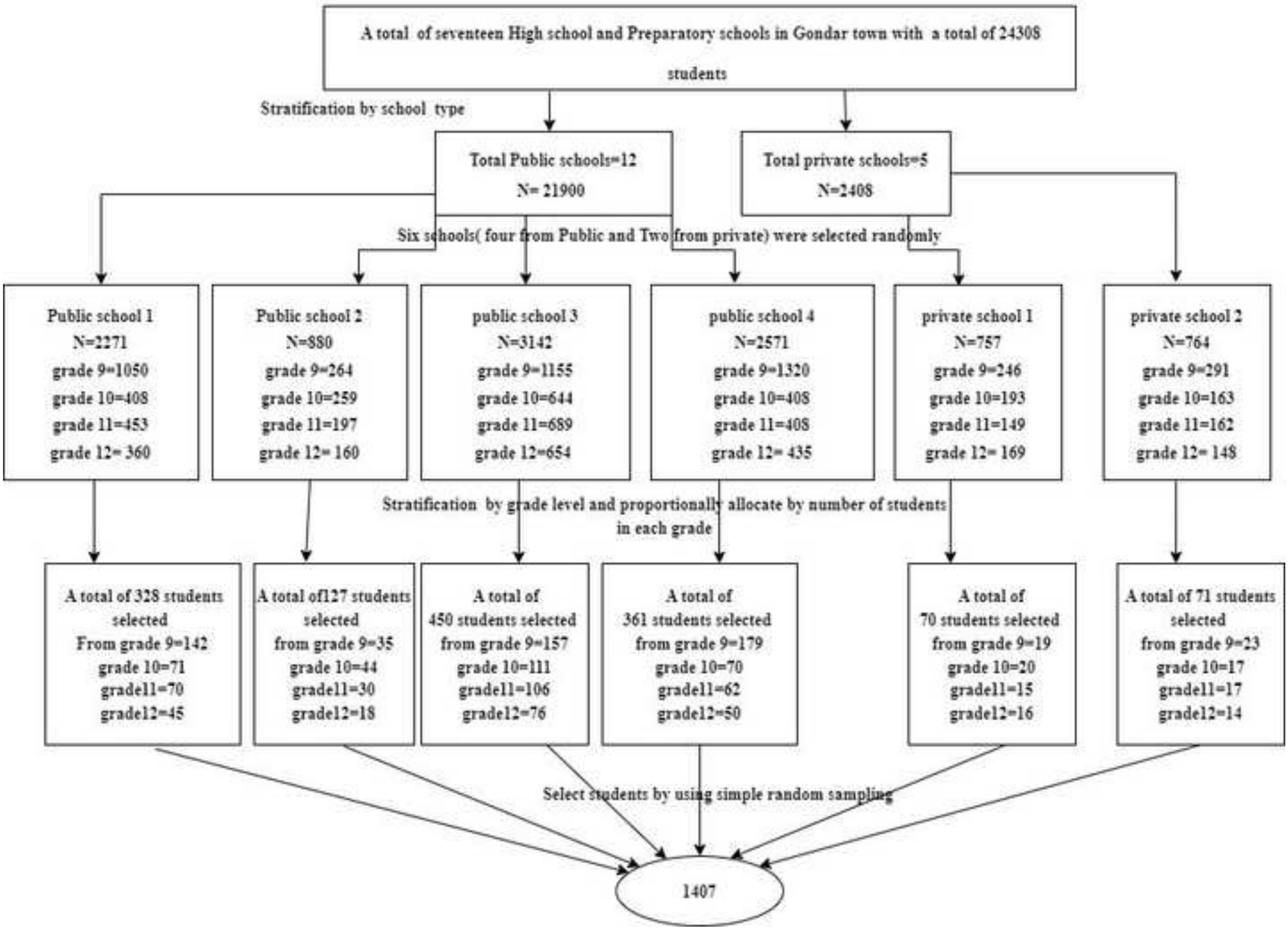

Click here to access/download;Figure;fig 4 final model.tif

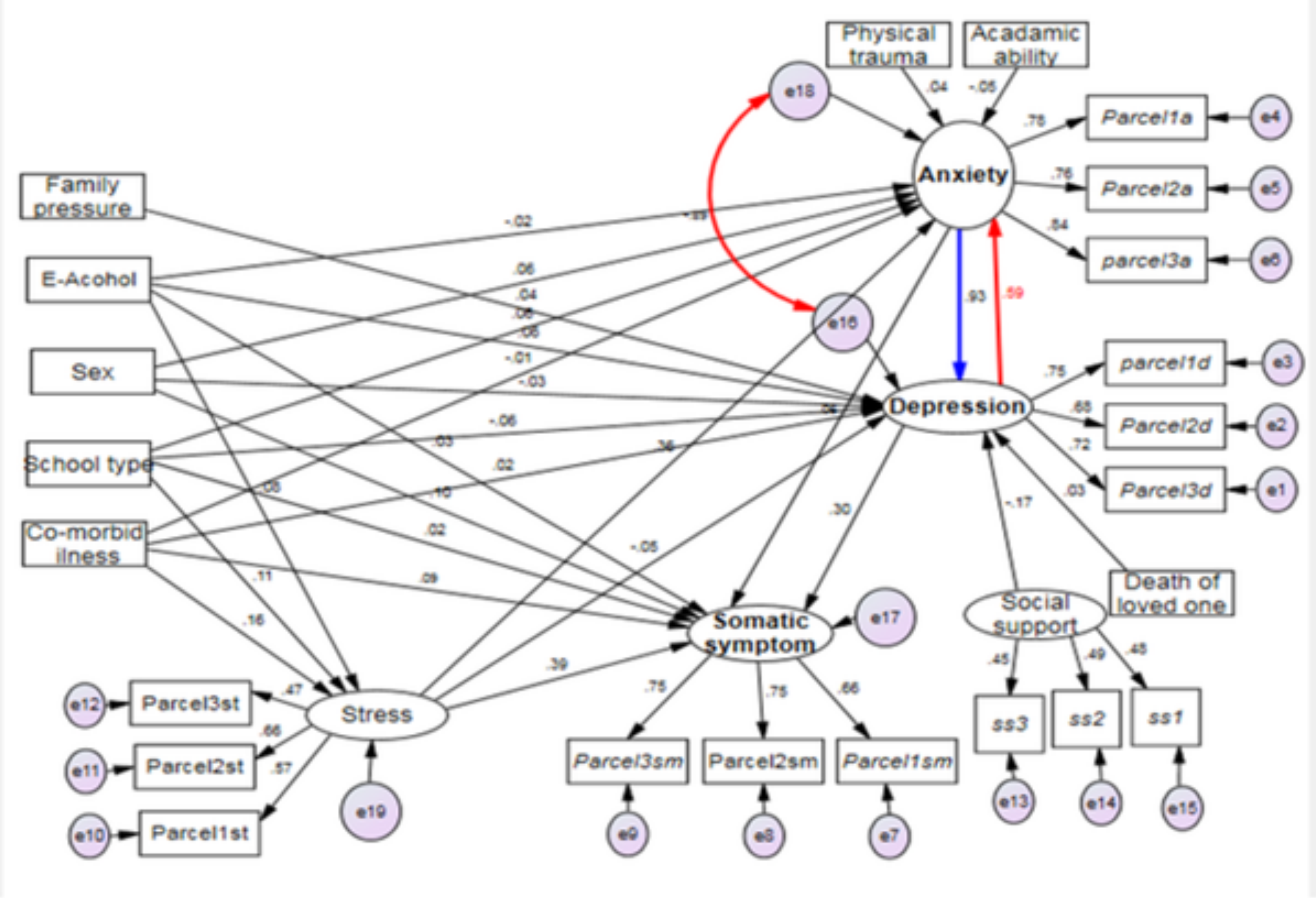

Click here to access/download
Supporting Information
S5 data set in excel format.xls

Click here to access/download
Supporting Information
Supplementary material 1.docx

Click here to access/download
Supporting Information
S2 fig 1.docx

Click here to access/download
Supporting Information
S3 annex 1 result of pilot study.docx

Click here to access/download

Supporting Information

supplementary material 4.rtf

Determinants of adolescents’~~adolescent’s~~ depression, anxiety, and somatic symptoms in Northwest Ethiopia: A Non-recursive structural equation modeling.

Zenebe Abebe Gebreegziabher[1*], Rediet Eristu[2], & Ayenew Molla[2]

[1] Department of Epidemiology and Biostatistics, School of Public Health, Asrat Woldeyes Health Science Campus, Debre Berhan University, Debre Berhan, Ethiopia

[2] Department of Epidemiology and Biostatistics, Institute of Public Health, College of Medicine and Health Sciences, University of Gondar, Gondar, Ethiopia

* Corresponding author

Email: zenebeabe7054@gmail.com (ZA)

Abstract

Introduction: In low and middle-income~~developing~~ countries, adolescent~~ including Ethiopia,~~ mental health is not only a major public health challenge~~concern;~~ but also a development concern.~~al issue.~~ Depression and anxiety are the most common~~commonest~~ mental health disorders and ~~often~~ somatic symptoms often co-exist with them. Adolescents with common mental health problems are associated with an increased risk of suicide, future unemployment, and poor quality of life. Little is known about the mental health of adolescents in ~~the~~ Ethiopia. Thus, this study aimed to assess the determinants of depression, anxiety, and somatic symptoms among adolescents in Northwest Ethiopia, in 2022.

Methods: An institution-~~Institution~~ based cross-sectional study was conducted from June 8 to 24, 2022. Two-stage stratified random sampling was used to select 1407 adolescents in Northwest Ethiopia. Structured and standardized self-administered questionnaires were used to collect the data. Non-recursive structural equation modeling was employed to assess the direct, indirect, and total effect~~s~~ of predictors. Adjusted regression coefficients and corresponding 95% confidence intervals were used to interpret the strength of the association.

Results: The prevalence of depression, anxiety~~,~~ and somatic symptoms were 28.21 (95% CI: 25.8, 31%), 25.05% (95% CI: 22.8, 27.5), and 25.24(95% CI: 23, 27.6%) respectively. Ever use of alcohol had a significant positive effect on depression [β= 0.14, 95% CI: 0.073, 0.201], anxiety [β= 0.11, 95% CI: 0.041, 0.188]~~,~~ and somatic symptoms [β= 0.12, 95% CI: 0.062, 0.211]. Stress had a significant positive effect on depression [β= 0.76, 95% CI: 0.642, 0.900], anxiety [β= 1.10, 95% CI: 0.955, 1.264], and somatic symptoms [β= 086, 95% C: 0.700, 1.025]. High levels of anxiety were substantially connected to higher levels of depression [β= 0.74, 95% CI: 0.483, 1.081], and depression had a direct positive influence on anxiety [β = 0.74, 95% CI: 0.508, 1.010].

Conclusion: In this study, the prevalence of depression, anxiety, and somatic symptoms was ~~were~~ moderate. Alcohol use and stress were significantly related to~~with~~ depression, anxiety, and somatic symptoms. The bidirectional relationship between anxiety and depression was significant. Therefore, public health interventions should focus on the bidirectional relationship between depression and anxiety, as well as on identified factors to reduce the burden of mental illness in adolescents.

Keywords: Anxiety,~~anxiety,~~ depression, somatic symptoms, adolescents, structural equation modeling, Ethiopia

Introduction

Mental health disorders account~~s~~ for 16% of ~~the~~ global burden of disease and injuries and more than 80% of people with mental health disorders live in low and middle~~-~~ income countries, with ~~an~~ increase in incidence and mortality [1].

Adolescence marks the transition from infancy to adulthood, involving physical, psychological, social, and cognitive development, leading to role confusion, stress, and emotional instability [2]. Adolescents are especially vulnerable to develop~~ing~~ mental illness [3]. In this population mental health disorders ~~is~~ increased by 32.18% from 1999 to 2019 [4]. According to the World Health Organization, mental health issues impact 10–20% of adolescents in the world~~wide~~ [5]. In low and middle-income countries, adolescent~~s~~ mental health is ~~-~~not only a major public health challenge~~,~~ but also a development concern [6].

Human suffering and financial costs associated with mental health disorders are substantial and growing globally [7]. More than 12 billion working days are lost annually as a result of mental illness. More than diabetes, cancer, and respiratory diseases combined, it is predicted that between 2011 and 2030, the world economy will lose 16 trillion US dollars due to mental illness [6]. Depression and anxiety are among the common mental health disorders [8], and ~~–~~often somatic symptoms coexist ~~co-exist~~ with them [9, 10].

Depression, which is the second most common mental health disorder in adolescents, is characterized by loss of interest or pleasure, feelings of guilt or low self-worth, feelings of tiredness, disturbed sleep or appetite, and poor concentration and sadness [11]. In 2018, over one million adolescents died from preventable causes, with depression playing a significant role [12]. In Asia, its prevalence ranges from 24.3% to 57.7% [13], and in Sub-Saharan Africa, it ranges from 15.5% [14] to 45.90% [15, 16]. In Ethiopia, 28% of adolescents in Jimma [17] ~~of~~

Formatted: Pattern: Clear

Formatted: Font color: Light Blue, Check spelling and grammar

~~adolescents in Jimma~~ and 36.2% of adolescents in Aksum [18] ~~of adolescents in Aksum~~ were depressed. Previous studies documented that~~-~~ age [16], female sex [17, 19-21], being in public school [22], high family academic pressure [23, 24], poor self-rated academic ability [25, 26], alcohol use [27, 28], cigarette smoking [29, 30], stress [31-33], somatic symptoms [17, 19, 20, 31, 34, 35], and anxiety [36, 37] had a positive relation with depression. Other~~While other~~ factors, such as ~~like~~ social support [25, 26, 34, 38], and family education [35] had, negative associations~~s~~ with depression.

Anxiety is characterized by emotional feelings of excessive fear, nervousness, avoiding threats in the environment perceived by them, and physical symptoms such as fast respiration, increased blood pressure, and tightness of the chest [39, 40]. Globally, 301.39 million people~~s~~ are affected by anxiety [41]. It ~~-~~is the leading cause of mental disorders in the world, accounting~~which accounts~~ for approximately~~about~~ 28.68 million disability-adjusted life years in the global burden of disease [42]. Anxiety is the most common~~commonest~~ mental health disorder in adolescents [43], with a high prevalence in developed as well as in developing countries [44]. In adolescents, it ranges from 42.1% [45] to 80.85% in Asia [46], and in sub-Saharan Africa,~~-~~ 29.8% of adolescents had anxiety [3]. A study conducted in Kenya showed that 37.99% of high school adolescents had anxiety [16]. A systematic review conducted among children and youth in Ethiopia revealed that general anxiety disorder ranges from 0.5-23% [47]. In combination with depression, anxiety contributes to~~for~~ 45% of the overall burden of disease [48]. Different pieces of literature ~~Literatures~~ reported that female sex [46, 49], age [16, 45], smoking [30, 50], alcohol use, low self-rated academic ability [45, 51], family academic pressure [23], stress [52, 53], somatic symptom disorder [54] and depression [55] have a~~had~~ positive relationship with anxiety.

A somatic~~Somatic~~ symptom is characterized by a comprehensive list of symptoms such as pain, breathlessness, numbness, palpitation, tiredness, headache, dizziness, and gastroenterological problems. Somatic symptom disorder is a disorder in which individuals excessively or disproportionally think or experience feelings~~-~~ about the symptoms, which results in significant disturbance in daily life [56]. Depression~~Often depression~~ and anxiety often coexist~~co-exists~~ with somatic symptoms [57-59], which is one of the remarkably widespread issues in primary health care and subspecialty settings [10]. Somatic symptoms result~~results~~ in more than half of the patient visits to~~in~~ primary health care [60]. Its prevalence is estimated to be 5 to 7% [61] in the general population and ~~-~~5 to 30~~-~~% [62, 63] in adolescents.~~adolescent.~~ Somatic symptoms delay the diagnosis of depression and anxiety~~,~~ because most people seek medical attention for bodily symptoms. Finally, individuals~~Individuals~~ with somatic symptoms may experience physical harm from unnecessary medical procedures [64]. Different scholars reported that female sex [65-67]~~-~~, being in private school [65], physical~~ly~~ inactivity [68, 69], extra school tutoring [70], stress [71-73], anxiety [66, 74, 75] and depression [66, 76, 77] had significant positive relations with somatic symptoms.~~symptom.~~

Although ~~Despite the fact that~~ mental health is a key part of ~~the~~ sustainable development goals that ~~which~~ affect every other goal~~s~~ [6], the prevalence of mental health disorders is still high [46]. To decrease the burden of mental illness, targeting adolescents is ~~-~~important; since more than half of mental health problems start~~s~~ at this age [6], and 50% of adolescent mental health problems will proceed to adulthood [78]. Adolescents in low and middle-income countries are disproportionally affected by mental health disorders; however, there is~~are~~ limited data on the prevalence and determinants of mental illness~~-~~ in Sub-Saharan Africa [79, 80].

In Ethiopia, al~~Due to the internal conflict in northern Ethiopia, adolescents in northwest Ethiopia are more prone to mental illness~~though mental health services were included in the national health policy, there is a paucity of evidence on the prevalence and determinants of anxiety, somatic symptoms, and their relation with depression. Even studies conducted outside of Ethiopia had methodological flaws, as they used logistic or linear regression to assess multiple mental health outcomes, which cannot address the bidirectional relationship between outcome variables and the indirect effect of predictors. In such cases, non-recursive structural equation modeling, which is a multivariate statistical framework used to measure the complex relationships between different observed and latent variables simultaneously is preferable. Adolescents in northwest Ethiopia are especially vulnerable to mental illness, due to repeated internal conflicts in northern Ethiopia [81].~~ In Ethiopia, though mental health services were included in the national health policy, there is a paucity of evidence on the prevalence and determinants of anxiety, somatic symptoms, and their relation with depression. Even studies conducted outside of Ethiopia had methodological flaws, as they used logistic or linear regression to assess multiple mental health outcomes, which cannot address the bidirectional relationship between outcome variables and the indirect effect of predictors. In such cases, non-recursive structural equation modeling, which is a multivariate statistical framework used to measure the complex relationships between different observed and latent variables simultaneously is preferable.~~

Therefore, this study intended to fill this gap by assessing the prevalence, relationship, and determinants of depression, anxiety, and somatic symptoms among high school and preparatory school adolescents in Gondar town by using non-recursive structural equation modeling. The findings from this study will help psychologists,~~the psychologist,~~ psychiatrists, policy~~-~~makers,

students, the community, and the government to tackle the increasing burden of mental health problems. Moreover, it will be used as ~~an~~ input for~~toward~~ sustainable development and as ~~a~~ baseline data for further research.

Methods and materials

Study design~~,~~ and context

An institution-~~Institution~~ based cross-sectional study was conducted from June 8 to -June 24, 2022, among high and preparatory school adolescents in Gondar town, Northwest, Ethiopia. It is located 728 kilometers from Ethiopia's capital city of Addis Ababa, and 180 kilometers from Bahir Dar, the capital city of the Amhara regional state. There are 395, 000 residents [82], and 48 healthcare facilities including one comprehensive specialized hospital, eight health centers, one private general hospital, fifteen specialist clinics, fifteen medium clinics, and eight primary clinics. Adolescents comprise ~~contains~~ more than 25% of the Ethiopian population and the health of adolescents determines the future development of the country. According to the town administrative educational department report, the town has seventeen (twelve public and five private high schools), with a total of 24,308 students. Among them, 2408 students were from private schools (1457 girls and 951 boys) and 21900 students were from public schools (12052 girls and 9848 boys).

Participants

All high and preparatory school adolescents in Gondar town who were registered for the second semester of the 2021-2022 academic year~~s~~ were included in the study. Night preparatory and high school students and students who were transferred in from other areas after January 8, 2022, were excluded.

The general rule for sample size calculation in structural equation modeling ranges from 5 to 20 times per free parameter~~s~~ [83]. In this study, ~~a~~ 5:1 ratio was used to obtain~~get~~ the minimum adequate sample size. Based on the hypothesized model ***Fig 1***, a total of 134 free parameters were available (***Fig 1***).

Considering 134 free parameters, the sample size was

134*5=670: Since the sampling procedure is multistage sampling with 2 stages, a design effect of 2 was considered.

Therefore, the sample size becomes 670*2 =1340

After adjusting for 5% for non-response rate, the final sample size became

1340+1340*5%=**1407**

Fig 1: Hypothetical structural equation modeling on the prevalence and determinants of depression, anxiety, and somatic symptoms among high school and preparatory school adolescents in Northwest Ethiopia, 2022 (both measurement and structural model): *Circles indicate latent variables or error~~s~~ terms or disturbances, rectangles indicate observed variables, single arrows indicate factor loadings or regression coefficients, and double arrows indicate the covariance between latent variables. A1-A7=items of anxiety, D1-D9=items for depression, S1-S8=~~-~~items for somatic ~~symptoms,~~symptom, ST1-ST10=items for stress, and e=error term.*

In this study, a two-stage stratified random sampling technique was used to select participants. A study conducted in Telangana state, India, showed that adolescents in public schools had a higher proportion of depression than those in private schools [22]. As a result, stratification by school type was performed~~done~~ and study subjects were proportionally allocated. Then, two ~~schools from~~ private schools and four ~~public~~ schools ~~from public~~ were

selected randomly. After that, heterogeneity by grade level was considered and, stratification was performed~~done~~ by grade level (***Fig 2***).

Fig 2: Diagrammatical representation of the sampling procedure that was used to assess the prevalence and determinants of depression, anxiety, and somatic symptoms among high school and preparatory school adolescents in Northwest Ethiopia, 2022. N indicates the~~N=~~ total number of students.

Variables of the study

Outcome variables (endogenous constructs):

Depression, anxiety, and somatic symptoms

Independent variables: summary of key variables used in the study**: S1 Table 1**

Data collection procedures and tools

Primary data in ~~with~~ pen and pencil format were gathered using a structured questionnaire through a self-reported questionnaire for those who could see and by face-to-face interview~~s~~ for 3 individuals who could not see. Four trained data collectors with ~~who have~~ a first degree in public health ~~officer~~ were assigned for data collection.

The questionnaire~~Questionnaire~~ was designed to gather data on socio-demographic characteristics, behavioral factors, academic-~~-~~related factors, ~~and~~ relationship-~~-~~related factors, clinical factors, depression, anxiety, somatic symptoms,~~symptom,~~ and stress domains (**Table 1**).

Table 1: Instruments~~instruments~~ used for the determinants~~determinates~~ of depression anxiety, somatic symptom, their description and criteria for categorization

**Variables**	**Instruments**	**Description**	**Criteria for categorization**	**Reference**

Depression	Patients Health Questionnaire 9 for adolescents (PHQ-9A)	It is a depression screening tool with 9 -items; -each item waswere measured by a Likertlikert scale ranging from 0 "not"not at all"all" to -3 "nearly"nearly every day"day" and its total score ranged from 0 to 27.	Students were classified as having depression, if their total score on the PHQ-9A was 10 or higher.	[84]
Anxiety	General Anxiety Scale-7 (GAS-7)	A brief measure of anxiety that contains seven items, which are rated onin four--point Likertlikert scales that range from 0 "not"not at all"all" to 3 nearly every day". Its summed score ranges from 0-21.	Students were classified as anxious, if their total score on the GAS-7 was 10 or higher.	[85]
Somatic symptom	Somatic symptom scale-8 (SSS-8)	It contains a total of 8 items, each item was measured by a Likert scale ranging from 0 "not at all" to 4"very much'; a total scoring range from minimal 0 to maximum 32.	Based on the SSS-8 score, students were classified as having low 4 to 7, medium 8 to 11, high 12 to 15, and very 16 to 32 very high somatic symptom levels.	[86]
Stress	Perceived Stress Scalestress scale 10 (PSS-10)	It contains 10 items. Each item scored with an ordinal scale of 0–4, where 0 represents "never" and 3 represents "very often". Individual scores range from 0 to 40.	Students who have a total perceived stress scale 10 scores of 0-13, 14-26, and 27-40 were categorized as having low, moderate, and high perceived stress levels respectively.	[87]

Social support	Oslo Social Support Scalesocial support scale three	It has 3 items; with a Likertlikert scale that ranges from 1 to 4 for the first item (number of people you can count if you face a great personal problem) and 1 to 5 ranges for the rest items. It's summed score ranges from 3 to 14.	Students were categorized as having poor social support 3–8 score, moderate social support 9–11 score, and strong social support 12-14 score.	[88].
Ever use of alcohol	Alcohol, smoking, and substance involvement test(ASSIST) was used to assess current and ever use of alcohol	Do you have a history of alcohol (like beer, Tella, Katikala, Wine) in-take in your life-time?<br><br>Do you drink alcohol within the past 3 months?	Respondents who answered yes were considered as positive for ever--use of alcohol<br><br>Students who answered yes were considered as positive for current alcohol user	[89]
Physical activity	Self--reported physical activity assessment for adolescents was used	In the past one week, how many days did you exercise for at least 60 minutes until youwe felt sweaty or shortness of breath	Respondents who answered 2 days and above were categorized as physically active; and those who answered one day and not were categorized as physically inactive.	[90]

Data quality assurance

To maintain the quality of the data, the questionnaire that was developed in the English language was translated in–to Amharic and re-–translated in–to English by another person to check consistency. Four public health experts and two psychiatrists checked the face validity of the

tool. Moreover, a pilot study was conducted among 201 students (177 from public schools and 24 from private schools) to check the validity and reliability of the tool. Reliability was checked by using Cronbach's alpha and the tool was reliable~~,~~ since ~~the~~ Cronbach's alpha values were above 0.7 for the~~both~~ overall and particular factors except for social support. For social support, since it had a small number of items (<5), the average inter-item covariance (AIIC) value was used, which~~it~~ was 0.4, which is in the acceptable range (0.2 to 0.4). The convergent and discriminate validity of the tool was also checked. The convergent validity was maintained~~,~~ since all standardized factor loadings were significant (p-~~-~~value <0.05) and the composite reliability was above 0.6. The discriminate validity of the tool was also evidenced~~,~~ since all inter-factor correlations were less than the square root of AVE **S3 annex 1**. A pilot~~Pilot~~ study was also used to check the way that each questionnaire was understood by different participants and modifications were made~~done~~ accordingly based on the feedback. Additionally,~~ the~~ Ramsey regression equation specification error test (RESET) was used to check omitted variable bias in the pilot study. In our study, the minimum Ramsey RESET p value was 0.2 **S3 annex 1**. Hence; there was no significant omitted variable bias [91].

Data processing, model building, and analysis

Collected data were coded and entered in~~-~~to Epi-data software version 4.6, and exported to STATA version 16 and AMOS version 21 for further analysis. Non-recursive structural equation modeling was employed to assess the complex relationship between different latent and observed variables. Model assumptions such as: specified and identified model, multivariate normality, missing, outlier, sample size adequacy, independency, common method bias, and strong and valid instrumental variable were checked.

Specified model: In this study, we specified the model after extensive literature searching and reading as presented in ***Fig 1***.

Identified Model: In this study, identification of non-recursive structural equation modeling with complete correlation of error terms was used. There are three methods~~ways~~ of identification in non-recursive structural equation modeling; which are: the presence of unique instrumental variables, the order condition, and the rank condition. Among these,~~this,~~ only the rank condition is sufficient for identification, and the remaining~~rest~~ two are necessary, but not sufficient [92]. In this study, all endogenous variables in the reciprocal loop had unique instrumental variables. Order condition: the number of excluded variables for that specific endogenous variable minus the total number of endogenous variables minus one should be greater than or equal to 0 [92]. In our model, to satisfy the order condition, each endogenous variable in the recursive loop should have at least 3-1 =2 excluded variables. For depression, anxiety, and somatic symptoms 9, 8, and 11 variables were excluded respectively. Therefore, the model fulfills the~~full fill~~ requirement of the order condition. Rank condition: In this particular study, there are 3 endogenous variables in the non-recursive loop ~~and~~ 17 exogenous variables, and one endogenous variable out of the feedback loop (***Fig 1***). Rank conditions~~Conditions~~ were determined~~done~~ using the system matrix. To formulate the system matrix, a separate equation for each endogenous variable in the feedback loop is mandatory. Therefore, the equations for depression, anxiety, and somatic symptoms disorder are presented as follows:

$$\textbf{\textit{Depression}}(y1) = \text{x2 alcohol use} + \text{x3 chronic illness} + \text{x4 physical activity} + \text{x5 family mental ilness} + \text{x7 school ty} + \text{x11 death of beloved one} + \text{x12 social support} + \text{x16family pressur} + \text{x18 stress} + \text{x20}(\text{y2}) + \text{x21}(\text{y3})\ \text{somaticsymptom}$$

$$\textbf{\textit{Anxiety}}(y2) = \text{x2 alcohol use} + \text{x3 chronic illness} + \text{x4 physical activity} + \text{x5 family mental ilness} + \text{x7 school type} + \text{x13 physical trauma} + \text{x17 acadamic ability} + \text{x18 stress} + x19(y1) + x21(y3)$$

$$\textbf{Somatic symptom}(y3) = x2 \text{ alcohol use} + x3 \text{ chronic illness} + x4 \text{ physical activity} + x5 \text{ family mental ilness} + x7 \text{ school type} + x14 \text{ study time} + x15 \text{ extra school tutoring} + x18 \text{ stress} + x19(y1) + x20(y2)$$

Rank conditions in the system matrix below begin, begins by constructing a system matrix based on the above equations. Endogenous variables are presented in the row and all variables are presented in the column. In each row, zero or one appears in the column that corresponds to that row. One- indicates that the variable represented by the column has a direct effect on the endogenous variable in that row or on the endogenous variable itself, and , 0 indicates the excluded variables for that specific endogenous variable in that row [92].

$$\begin{bmatrix} x & 2 & 3 & 4 & 5 & 6 & 7 & 8 & 9 & 10 & 11 & 12 & 13 & 14 & 15 & 16 & 17 & 18 & y1 & y2 & y3 \\ y1 & 1 & 1 & 1 & 1 & 0 & 1 & 0 & 0 & 0 & 1 & 1 & 0 & 0 & 0 & 1 & 0 & 1 & 1 & 1 & 1 \\ y2 & 1 & 1 & 1 & 1 & 0 & 1 & 0 & 0 & 0 & 0 & 0 & 1 & 0 & 0 & 0 & 1 & 1 & 1 & 1 & 1 \\ y3 & 1 & 1 & 1 & 1 & 0 & 1 & 0 & 0 & 0 & 0 & 0 & 0 & 1 & 1 & 0 & 0 & 1 & 1 & 1 & 1 \end{bmatrix}$$

The following steps were followed for the identification of rank condition

1. Start with the first row of the system matrix. Cross out all entries in that row and cross out any column in the system matrix with a one in that same row.
2. Simplify the reduced matrix further by deleting any row with all zeros entries; delete any row that is an exact duplicate of another or can be reproduced by adding other rows (i.e., it is a linear combination of other rows). The number of remaining rows is the rank.

If the rank of the reduced matrix is larger than or equal to the sum of the endogenous variables in the recursive loop minus one, then the rank condition is satisfied [92].

The rank condition for this particular study became;

For depression (y1), after we cross the entire first row and the entire column thatwhich contains 1 in the row:

The reduced matrix becomes:

$$\begin{bmatrix} 1 & 0 \\ 0 & 1 \end{bmatrix} \text{Rank=2}$$

For anxiety (y2), after we cross the entire second row and the entire column that~~which~~ contains 1 in that row:

The reduced matrix becomes:

$$\begin{bmatrix} 1 & 0 \\ 0 & 1 \end{bmatrix} \quad \text{Rank=2}$$

For Somatic symptom (y3), after we cross the entire third row and the entire column that~~which~~ contains one in that row:

The reduced~~Reduced~~ matrix becomes:

$$\begin{bmatrix} 1 & 0 \\ 0 & 1 \end{bmatrix} \text{Rank=2}$$

The hypothesized model was identified~~,~~ since all endogenous variables have a rank of two; which is equal to the number of endogenous variables in the feedback loop minus one.

Multivariate normality, outliers, and missing: Full information maximum likelihood estimation was used. Multivariate normality was assessed using Mardia’s kurtosis and its critical ratio. The data were not normally distributed, since Mardia’s kurtosis was above 7 and its critical ratio was above 5 [92]. Although item parceling and outlier deletion were attempted,~~tried to handle it,~~ there was no improvement. As a result, bootstrap maximum likelihood estimation with 3000 samples was used.

Mahalanobis distance p values less than 0.001 based on chi-square distribution were used to declare observation as a multivariate outlier [93]. There were 62 observations with Mahalanobis distance p-values less than 0.001. The data were examined to determine~~see~~ whether

they were ~~actually~~ outliers or caused by data entry errors. However, thisit was not due to a data entry error, and as mentioned above, multivariate normality did not improve significantly with their exclusion. Therefore,~~So,~~ those outlier observations were kept~~ in order~~ to maintain the study's power.

From the total observations, there were six observations with missing values. All missing values were from predictors and had no relationship with the outcome variable; thus, list-wise deletion was performed because they constituted less than 5% of the total sample and were considered missing completely at random, so 1373 observations were used for the final analysis [94].

Sample size adequacy and strength of correlation: The critical ratio for Bartlett's test of sphericity in this study was high and ~~it was~~ significant for all constructs. KMO was above 0.7 ~~for the~~ overall, as well as for specific constructs. Hence, the sample is sufficient, the population matrix was not identified,~~identity,~~ and factor analysis was evidenced [92] (**Table 2**).

Table 2*: KMO and Bartlett's test of sphericity for the measurement of depression, anxiety~~,~~ and somatic symptoms among high and preparatory school adolescents in Northwest Ethiopia, 2022.*

Factors	KMO	Bartlett's tests of sphericity	
		Chi-square	P value
Anxiety	0.88	3100	0.0001
Depression	0.87	2391	0.0001
Somatic	0.85	2155	0.0001
Stress	0.86	3354	0.0001
Overall	0.94	13938	0.0001

Formatted: Font: Not Bold
Formatted: Font: Not Bold
Formatted: Font: Not Bold
Formatted: Font: Not Bold
Formatted: Font: Not Bold
Formatted: Font: Not Bold
Formatted: Font: Not Bold

Independency of observations: The intra-~~-~~class correlation coefficients (ICCs)~~(ICC)~~ were used to test the independence of observations. School type and grade level were employed

as clustering variables. The ICC was less than 0.1 in our study (**S1 Table 2**),~~2,~~ and a classical structural equation model was used.

Common Method Bias: Harman's one-factor test was used to check common method bias (CMB). There was no CMB in this study~~investigation,~~ since the overall variance covered by one factor was 25.2%, which is below the suggested cut-off~~cutoff~~ (50%) [95].

Strong and valid instrumental variables: The strength~~Strength~~ and validity of instrumental variables were checked by using the Cragg-Donald Wald F statistic and Sargan Hansen test, respectively. A Sargan Hansen test P-value greater than 0.05 was used to ponder valid instruments, and F-test values above 10 were ~~-~~used as a cut-off ~~of~~ point to declare strong IVs [96]. In the present study, the strength and validity of the instrumental variables between anxiety and somatic symptoms, and between somatic symptoms and depression were invalid and weak. As a result, the non-recursive paths from somatic symptoms to anxiety and depression were excluded; since deleting a non-recursive path is one treatment for a weak and faulty instrument. Finally, an identified model with valid and strong instrumental variables in a non-recursive loop was preserved in **S1 Table 3**.

Ethical consideration

Ethical approval was obtained from the Institutional Review Board of ~~the~~ University of Gondar Institute of Public Health. The ethical approval letter was submitted to the Gondar town administrative educational department and ~~to~~ all selected high schools and preparatory schools. Permission was obtained from school directors. Parental informed consent was waived as the study had no/~~-~~minimal risk [97]. Following permission from the school directors, each student was given a detailed participant information sheet and they completed an assent form to indicate their willingness to participate. Only students who signed an assent form were eligible to take

part. Students with moderate to severe anxiety, depression, or somatic symptoms were referred to the University of Gondar Comprehensive Specialized Hospital Psychiatry Clinic.specialized hospital psychiatry clinic. To ensure the privacy and confidentiality of participants, all information gathered was kept in a locked cabinet in the investigator's home, no personal identifiers were mentioned, and the collected data were was used solely for research purposes. Overall, this study was carried out perin accordance with the Helsinki Declaration.

Results

Socio-demographic and clinical factors of respondents

Out of 1407 randomly selected individuals, 1379 completed the questionnaire; hence, the response rate was 98-%. The mean (± SD) age of the respondents was 17.23 ± 1.25 years with a range of 15 to 19 years. The majority, Majority, 1238(89.78%) of the respondents were from public schools, and regarding residence, two-thirds 915(66.35%) of respondents were from an urban areas. ApproximatelyAround half, (654, 654(47.43%) of the respondents had moderate stress. The majority, Majority, 1237 (89.7%), of the respondents did had not have a history of chronic illness (***Table 3***).

Table 3: Socio--demographic and clinical characteristics of high and preparatory school adolescents in Northwest Ethiopia, 2022 (n=1379).

**Variable**	**Frequency**	**Percentage (%)**
**Sex**		
Male	536	38.87
Female	843	61.13
**Age**		
15	140	10.20
16	291	21.19
17	297	21.63

18	405	29.50
19	240	17.48
**School type**		
Public	1238	89.78
Private	141	10.22
**Grade Level ~~level~~**		
Grade 9	544	39.45
Grade 10	325	23.57
Grade 11	296	21.46
Grade 12	214	15.52
**Residence**		
Urban	915	66.35
Rural	464	33.65
**Physical trauma**		
No	1076	78.03
Yes	303	21.97
**Medically confirmed chronic illness**		
No	1237	89.7
Yes	142	10.3
**Stress**		
Low	653	47.35
Moderate	654	47.43
High	72	5.22
**Family history of mental illness**		
No	1278	92.68
Yes	101	7.32

Behavioral, academic, and relationship-~~-~~related factors

More than one-third, (493, ~~493(~~35.75%) of the respondents had a history of alcohol consumption. ~~drinking.~~ Regarding physical activity, only 224(16.24%) of the respondents were physically active. The majority, ~~Majority,~~ 656 (47.57%) of the respondents, had good self-rated academic ability. ~~.~~When we see perceived social support, two-fifths of 564(40.9%) of the respondents had low perceived social support (***Table 4***).

Table 4: Behavioral, academic, and relation-related factors of high and preparatory school adolescents in Northwest Ethiopia, 2022 (n=1379).

Variables	Frequency	Percentage (%)
**Physical activity**		
Inactive	1155	83.76
Active	224	16.24
**Ever use of alcohol**		
No	886	64.25
Yes	493	35.75
**Extra school tutoring*(n=1378)**		
No	1011	73.3
Yes	367	26.7
**Self-rated academic ability**		
Very poor	39	2.83
Poor	70	5.08
Good	656	47.57
Very good	390	28.28
Excellent	224	16.24
**Father education *(n=1377)**		
Can't read and write	266	19.29
Grade 1- 8	488	35.43
Grade 9-12	308	22.34
Diploma and above	315	22.84
**Mother education* (n=1378)**		
Can't read and write	429	31.12
Grade 1- 8	436	31.63
Grade 9-12	277	20.10
Diploma and above	236	1.13
**Family academic pressure**		
Not at all	524	38
Low	375	27.19
Medium	144	10.44
High	336	24.37
**Death of a loved one in the past 6 months**		
No	1058	76.72
Yes	321	23.28
**Social support**		

Poor	564	40.9
Medium	559	40.54
Good	256	18.56
**Current alcohol use(n=1377)***		
No	368	26.73
Yes	1009	73.27
	Median	IQR
**Study time in hours(n=1379)~~hour(n=1379)~~**	3	±3
**Time spent~~spend~~ for extra school tutoring** in hour(n=367)	**2**	±2

* Indicates variables with missing observations

Prevalence of depression

In this study, the overall prevalence of self-reported depression was 28.21(95% CI= 25.8, 31%)**.** Regarding severity, 34.8% had mild depression, 18.5% had moderate depression, 7.72% had moderately severe depression and 2.04% o~~f respondents~~ had severe depression. More than one-third, 34.8% of participants had a feeling of hurting themselves~~oneself~~ or betting~~better~~ off dead~~,~~ and more than two-thirds of the respondents (68.3%) had little interest or pleasure in doing things (**S1 Table 4).~~4.~~**

Prevalence of Anxiety

The overall prevalence of self-reported anxiety in this study was 25.05% (95% CI: 22.8, 27.5%). Regarding the level of anxiety, 34.08% of respondents had mild anxiety, 16.82% had moderate anxiety, and 8.27% had ~~%~~ severe anxiety. Among the total respondents, 16.61% worried too much about different things nearly every day, and 11.54% were ~~% could~~ not able to stop worrying too much nearly every day. More than half (58.38%) of the respondents had a feeling of anxiety~~anxious~~ or nervousness (**S1 Table 5).~~5.~~**

Prevalence of somatic symptoms

In this study, the overall prevalence of self-reported SSD~~somatic symptom disorder~~ was 25.24 % (95% CI= 23, 27.6%). Regarding its level, among the total respondents, 27.27 % had mild, 15.88% had moderate, 8.19% had high, and 6.38% had very high somatic symptoms. Regarding ~~respectively. When we see~~ the response to specific indicators, approximately,~~around~~ two-thirds (65.42%) of the participants had ~~headaches,~~headache, more than one-half (53.68%) of the respondents had dizziness and 35.97% of the respondent~~s~~ had abdominal pain or gastrointestinal problems (**S1 Table** **6)**.~~6.~~

Structural equation modeling

Confirmatory factor analysis (CFA) was computed to test the measurement model. The model fit measures (CMIN/DF, CFI, TLI, and RMSEA) were used to assess the model's~~model~~ overall goodness of fit. In the hypothesized measurement mod**e**l **S2 Fig 1,** the goodness of fit was below the acceptable level: CMIN/DF=5.10, CFI=0.81 and RMSEA=0.05. To improve the fit of the model~~-~~, factorial item parceling was performed~~were done~~ [98] **S1 Table 7**. After that, the model fits were improved (CMIN/DF=2.60, CFI=0.99, TLI=0.99~~,~~ and RMSEA=0.001(***Fig 3***)**.**

Fig 3: Final measurement model for the constructs of anxiety, depression, somatic symptoms, stress, and social support.

Key: a =anxiety, d= depression, SO=somatic symptoms, st=stress, SS= social support, e =error term, and bidirectional arrow indicates covariance

For the model containing the structural and measurement parts, model comparisons were performed~~done~~ using AIC, CMIN/DF, TLI, CFI, and RMSEA. The model~~Model~~ with only

significant variables with significant disturbance after the parcel; which had small AIC, small CMIN/DF, and RMSEA, and~~-~~ large CFI and TLI was selected as a good fitted model (***Table 5***).

Table 5: Model selection for determinants of depression, anxiety, and somatic symptoms among high and preparatory school adolescents in Northwest Ethiopia, 2022.

Models	AIC	CMIN/DF	CFI	TLI	RMSEA	Remark
A model~~Model~~ with all predictors and covariance after the parcel	2468	6.8	0.8	0.7	0.1	
A model~~Model~~ with only significant variables with significant disturbance after the parcel	951	3.00	0.94	0.92	0.027	**selected**

Factors related to~~with~~ depression, anxiety, and somatic symptoms

Self-rated academic ability, perceived social support, physical trauma, death of~~-~~ loved one, ever use of alcohol, having medically confirmed chronic illness, sex, family pressure, school type, and stress were all significantly related to anxiety, depression, and somatic symptoms, either directly or indirectly. There was a significant bidirectional relationship between anxiety and depression (***Fig 4, Table 6***, ***Table 7*, and *Table 8***).

Fig 4: Standardized structural equation modeling for factors related to~~with~~ anxiety, depression, and somatic symptoms among high and preparatory school adolescents in Gondar town, Northwest, Ethiopia, 2022.

Factors related to~~with~~ depression among high and preparatory school adolescents in Gondar town, 2022.

Perceived social support, having a history of the death of a loved one within the past six months, and anxiety had ~~a~~ significant direct effect on depression. However,~~While,~~ having medically confirmed chronic illness, having a history of alcohol use, perceived family academic pressure, sex, stress, self-rated academic ability, and having a history of physical trauma~~ itself~~

Formatted: Font: Not Bold
Formatted: Font: Not Bold
Formatted: Font: Not Bold
Formatted: Font: Not Bold
Formatted: Font: Not Bold
Formatted: Font: Not Bold
Formatted: Font: Not Bold
Formatted: Font: Not Bold
Formatted: Font: Not Bold

had a statistically significant indirect relationship with depression. School type and anxiety were significantly related to depression both directly and~~as well as~~ indirectly.

Anxiety [adjusted β= 0.74, 95% CI: 0.483, 1.081] and perceived social support [adjusted β= -0.13, 95% CI: -0.229,-0.029] had~~has~~ a positive direct effect on depression. Self-rated academic ability had a significant indirect effect [adjusted β=-0.05, 95% CI: -0.083,-0.031] on depression (***Table 6***).

Table 6: Direct, indirect~~,~~ and total effects of socio-demographic, behavioral, relationship~~-~~ related factors and clinical factors on depression among adolescents in Northwest Ethiopia, 2022: ***Unstandardized estimate.***

Variables	Direct effect	Indirect effect	Total effect
**DV: Depression**	Estimate (95%CI)	Estimate(95% CI)	Estimate (95%CI)
Social support	-0.13[-0.229,-0.029]*	----	-0.13[-0.229,-0.029]*
Death of a loved one	0.03[0.004, 0.087]*	-----	0.03[0.004, 0.087]*
Self-rated academic ability	----	-0.05[-0.083, -0.031]*	-0.05[-0.083, -0.031]*
Physical trauma	----	0.10[0.037, 0.166]*	0.10[0.037, 0.166]*
Ever use of alcohol	0.06[-0.004, 0.112]	0.08[0.029, 0.139]*	0.14[0.073, 0.201]*
History of chronic illness	0.04[-0.053, 0.124]	0.17[0.085, 0.265]*	0.21[0.114, 0.311]*
Sex	-0.03[-0.099, 0.025]	0.09[0.042,0.162]*	0.06[0.003, 0.109]*
School type	-0.09[-0.194, -0.004]*	0.20[0.113, 0.320] *	0.11[0.016 , 0.207]*
Family pressure	0.02[-0.004, 0.040]	0.02[0.002, 0.043]*	0.04[0.016, 0.060]*
Stress	-0.06[-0,454, 0.247]	0.82[0.530, 1.250]*	0.76[0.642, 0.900]*
Anxiety	0.74[0.483, 1.081*]		0.74[0.483, 1.081*]

**Indicates significant variables, DV= dependent variable, SE= standard error, and CI=confidence interval*

Factors related to anxiety among high and preparatory school adolescents

Self-rated academic ability and physical trauma had ~~a~~ significant direct effect on anxiety. However,~~While,~~ ever use of alcohol, and having a medically confirmed chronic illness had ~~a~~ significant indirect effect on anxiety. School type, sex, stress, and depression had statistically significant direct and indirect relationships with anxiety.

Depression [adjusted β= 0.74, 95% CI: 0.508, 1.010] and having a history of physical trauma [adjusted β= 0.06, 95% CI: 0.014, 0.125] had ~~a~~ positive direct effect on anxiety. Self-rated academic ability had a direct negative effect [adjusted β= -0.03, 95% CI: -0.065, -0.006] on anxiety. Perceived social support had a significant negative indirect effect [adjusted β= -0.22, 95% CI: -.304, -0.139] on anxiety. Stress had both direct [adjusted β= 0.54, 95% CI: 0.293, 0.745] and indirect [adjusted β= 0.57, 95% CI: 0.379, 0.814] positive effect on anxiety, bringing a total positive effect of 1.10 [adjusted β= 1.10, 95% CI: 0.955, 1.264] (***Table** 7*).

*Table 7: Direct, indirect~~,~~ and total effect of socio-demographic, behavioral, relationship-related, and clinical factors on anxiety among adolescents in Northwest Ethiopia, 2022**:*** ***Unstandardized estimate.***

**Variables**	Direct effect	Indirect effect	Total effect
**DV: Anxiety**	Estimate (95% CI)	Estimate (95%CI)	Estimate (95% CI)
Self-rated academic ability	-0.03[-0.065, 0.006] *	----	-0.03[-0.065, 0.006]*
Physical trauma	0.06[0.014, 0.125]*	----	0.06[0.014, 0.125] *
Social support	----	-0.22[-0.304,-0.139] *	-0.22[-0.304,-0.139]*
Death of a loved one	----	0.06[0.010, 0.122] *	0.06[0.010, 0.122]*
Ever use of alcohol	-0.03[0.088, 0.035]	0.14[0.071, 0.213] *	0.11[0.041, 0.188] *
History of chronic	-0.02[-0.124, 0.078]	0.27[0.165, 0.384] *	0.25[0.132 , 0.371] *

illness			
Sex	0.08[0.019, 0.135] *	0.04[0.004, 0.092] *	0.12[0.059, 0.179] *
School type	0.12[0.017, 0.226] *	0.16[0.056, 0.269] *	0.28[0.159, 0.415] *
Family academic pressure	----	0.03[0.012, 0.050] *	0.03[0.001, 0.50] *
Stress	0.54[0.293, 0.745] *	0.57[0.379, 0.814] *	1.10[0.955, 1.264]*
Depression	0.74[0.508, 1.010] *	----	0.74[0.508, 1.010] *

**Indicates significant variables, DV= dependent variable, SE= standard error, and CI=confidence interval*

Factors related to~~with~~ somatic symptoms among high and preparatory school adolescents

Self-rated academic ability, perceived social support, physical trauma, and ~~the~~ death of a loved one in the past 6 months had a significant indirect effect on somatic symptoms. However,~~While,~~ having a medically confirmed chronic illness, sex, stress, and depression were all directly and indirectly related to somatic symptoms.

Social support [adjusted β =-0.11, 95% CI: -0.176, -0.067] and self-rated academic ability [adjusted β =-0.02, 95% CI: -0.039, -010] had a significant negative indirect effect on somatic symptoms. Having medically confirmed chronic illness had significant direct [adjusted β= 0.17, 95% CI= 0.045, 0.290] and indirect [adjusted β= 0.20, 95% CI: 0.122, 0.289] effects on somatic symptoms, bringing a total positive effect of 0.37 [adjusted β= 0.37, 95% CI: 0.239, 0.505]. Stress had both direct [adjusted β= 0.53, 95% CI: 0.326, 0.768] and indirect [adjusted β= 0.33, 95% CI: 0.186, 0.472] positive effect on somatic symptoms resulting in a total positive effect of 0.86 [adjusted β= 086, 95% CI: 0.700, 1.025]. Depression had significant direct [adjusted β= 0.38, 95% CI= 0.167, 0.540] and indirect [adjusted β= 0.58, 95% CI: 0. 0.167, 3.629] positive

effect on somatic symptoms resulting in a total positive effect of 0.96 [adjusted β= 0.96, 95% CI: 0.433, 1.456] (***Table** 8*).

*Table 8: Direct, indirect, and total effects of socio-demographic, personal, relationship-related factors, stress, anxiety, and depression on somatic symptom disorders among adolescents in Northwest Ethiopia, 2022**: Unstandardized estimate**.*

**Variables**	**Direct effect**	**Indirect effect**	**Total effect**
**DV: Somatic symptoms**	Estimate (95%)	Estimate (95%)	Estimate (95%)
Self-rated academic ability	----	-0.02[-0.039, -010]*	-0.02[-0.039, -010]*
Physical trauma	----	0.04[0.012, 0.083]*	0.04[0.012, 0.083]*
Social support	----	-0.11[-0.176, -0.067]*	-0.11[-0.176, -0.067]*
Death of a loved one within the past 6 months	----	0.03[0.005, 0.067]*	0.03[0.005, 0.067]*
Ever use of alcohol	0.03[-0.022, 0.105]	0.09[0.044, 0.144]*	0.12[0.062, 0.211]*
History of medically confirmed chronic illness	0.17[0.045, 0.290]*	0.20[0.122, 0.289]*	0.37[0.239 , 0.505]*
Sex	0.11[0.050, 0.166]*	0.03[0.002, 0.053]*	0.14[0.074, 0.198]*
School type	0.03[-0.074, 0.144]	0.13[0.053, 0.216]*	0.16[0.046, 0.292]*
Stress	0.53[0.326, 0.768]*	0.33[0.186, 0.472]*	0.86[0.700, 1.025]*
Depression	0.38[0.167, 0.540]*	0.58[0.167, 3.629]*	0.96[0.433, 3.774]*
Anxiety	0.06[-0.101, 0.212]	0.60[0.243, 3.825]*	0.66[0.270, 3.825]*

**Indicates significant variables, DV= dependent variable, SE= standard error, and CI=confidence interval*

Discussion

In this study, the prevalence and determinants of depression, anxiety~~,~~ and somatic symptoms were examined using non-recursive structural equation modeling. The prevalence rates ~~Prevalence~~ of depression, anxiety, and somatic symptoms were 28.21% [95% CI: 25.8%, 31%], 35.82% [(95% CI: 33.3%, 38.4%), and 25.24% [95% CI: 23.0%, 27.6%] respectively. Sex, alcohol use, stress, self-rated academic ability, perceived social support, school type, and physical trauma were significant determinants of anxiety, depression, and somatic symptoms. The bidirectional relationship between anxiety and depression was significant. Depression and anxiety were significant determinants of somatic symptoms.

Prevalence of depression, anxiety, and somatic symptoms

In the current study, the overall prevalence of self-reported depression was 28.21% (~~%(~~ 95% CI= 25.8%, 31%). This is in line with the study conducted in Jimma 28% [17]~~.~~, but it was higher than the meta-analysis conducted in China (24.3%) [13], and lower than the studies~~study~~ conducted in Aksum (38.2%) [18], Nepal (44.2%) [99], Bangladesh (36%) [100], and India (57.7%) [101]. In this study~~,~~ the prevalence of anxiety was 25.05% (95% CI: 22.8, 27.5%). This~~which~~ is consistent with the study conducted among children and youth in Ethiopia 0.5 to 23% [47]. However, it is lower than the study conducted in Kenya ~~at~~ 37.99% [16], Saudi Arabia ~~at~~ 63.5% to 66% [102, 103], Jordan ~~at~~ 42.1% [45], and Chandigarh ~~at~~ 80.85% [46]. In contrast, ~~to this,~~ it was higher than the worldwide estimates of anxiety among children and adolescents (6.5 to 10%). In our study, the prevalence of self-reported somatic symptom disorders was 25.24% (95% CI= 23, 27.6%). This is consistent with the study conducted among children and adolescents in Tarragona which reported 5 to 30% [62]. However, it was higher than the prevalence in ~~the~~ general population (5–7%) [104] and lower than that in a study

conducted in Qatar (47.8% in female~~s~~ and 52.2% in ~~males)~~male) [105]. This disparity could be explained by differences in screening tools and cutoff points [18, 46, 106]. Furthermore, since mental health is the combination of physical, social, cultural, and religious environments this could be explained by socio-cultural differences in different settings. Poverty, violence, and other stressful social conditions are not unique to any one region of the world, nor are the symptoms and manifestations that result from them. However, factors that are frequently associated with race or ethnicity, such as socioeconomic position or country of origin, can enhance the likelihood of being exposed to these sorts of stressors~~,~~ and thus stressors may lead~~s~~ to depression, anxiety~~,~~ and somatic symptoms [107]. Additionally, internal conflict in northern Ethiopia may raise the frequency ~~of~~ depression, anxiety~~,~~ and somatic symptoms [81].

Factors related to~~with~~ depression, anxiety, and somatic symptoms

Female sex had a statistically significant positive effect on depression [adjusted β= 0.06, 95% CI: 0.003, 0.109]~~-~~, anxiety [adjusted β= 0.12, 95% CI: 0.059, 0.179], and somatic symptoms [adjusted β=0.13, 95% CI: 0.074, ~~–~~0.198]~~-~~; this implies that being female increases the levels ~~of level~~ of depression, anxiety, and somatic symptoms ~~as~~ compared to being male, controlling for other factors. ~~constant.~~ This finding is consistent with other studies [17, 19-21]. This could be explained by the fact that men and women have different brain chemistry and hormonal variations. Menstrual fluctuations in progesterone and estrogen may affect neurotransmitters in women, and low neurotransmitters may lead to depression, anxiety, and somatic symptoms. Moreover, this~~it~~ might be due to the existence of androgen receptors in males, which may provide protection [108]. It could also be that females are more likely to report on their mental illness. Cultures differ in how men and women deal with issues such as~~like~~ depression, which

may influence how open or unwilling men and women are to reveal being depressed or having other mental health disorders [65, 66, 109-111].

Ever use of alcohol had a positive effect on depression [β= 0.14, 95% CI: 0.073, 0.201], anxiety [adjusted β= 0.11, 95% CI: 0.041, 0.188], and somatic symptoms [adjusted β= 0.12, 95% CI: 0.062, 0.211]. This implied that adolescents who had a history of alcohol drinking had higher levels of depression, anxiety, and somatic symptoms than those~~as compared to~~ who did not drink. This finding is congruent with the study conducted in the USA [27] and in China [28, 112]. This could be because~~due to the fact that~~ alcohol affects brain chemicals such as~~like~~ serotonin and dopamine, which are responsible for happiness, and ~~–~~the decrease in these~~thus~~ chemicals leads to feelings of depression~~ down and depressed~~ [113]. Furthermore, alcohol consumption may contribute to sadness, anxiety, and somatic symptoms in adolescents by reducing age-appropriate activities such as physical activity and social relationships [114]. Moreover, it could be due to the direct effect of ethanol on nerve cells [115] which leads to heightened awareness of normal body sensations.~~sensation.~~

Stress had a significant positive effect on depression [adjusted β= 0.76, 95% CI: 0.642, 0.900], anxiety [adjusted β= 1.10, 95% CI: 0.955, 1.264], and somatic symptoms [adjusted β= 086, 95% C: 0.700, 1.025]. This indicates that participants with high levels~~ level~~ of stress had higher levels of depression, anxiety, and somatic symptoms, holding other predictors constant. Our finding is supported by~~with~~ different studies~~literatures~~ [31, 32, 71]. The possible justification for this could be ~~owing to the fact~~ that high-level stress impairs ~~the~~ brain function, which may lead to fluctuations in neurotransmitters such as dopamine, which ~~—~~leads to depression. In addition, immune dysregulation during stressful~~stress full~~ life events leads to anxiety, and anxiety leads to depression and depression may lead~~s~~ to somatic symptoms.

As the self-rated academic ability of adolescents increases, the levels of depression [adjusted β=-0.05, 95% CI: -0.083,-0.031], anxiety [adjusted β= -0.03, 95% CI: -0.065, -0.006], and somatic symptoms [adjusted β =-0.02, 95% CI: -0.039, -010] decrease. This result is compatible with the study conducted in Iran [116], ~~and~~ Slovakia [117] and with other studies [45, 51, 118]. The possible explanation for this might be, ~~that~~ students with low academic ability are terrified of negative responses from teachers, parents, and friends, which may lead to fear about their future career, which will lead them to become anxious, and anxiety will lead to depression and somatic symptoms.

Perceived social support had a significant direct negative effect [adjusted β= -0.13, 95% CI: -0.229,-0.029] on depression. This means that as social support increased,~~increases,~~ the level of depression decreased or students with low social support had higher levels of depression than~~as compared to~~ those who had high social support. This finding is compatible with different reports [25, 26, 34, 38]. The probable reason for this might be that low social support may increase feeling~~s~~ of loneliness, worthlessness, and hopelessness,~~hopelessness;~~ leading to a loss of interest in activities and depression. Moreover, students with low social support are more likely to have low self-esteem and negative cognition than those with higher social support, which may lead to depression [119].

Learning in a private school had a statistically significant positive effect [adjusted β= 0.10, 95% CI: 0.016, 0.207] on depression. ~~This means~~Means that participants in private schools had ~~a~~ higher levels of depression than~~compared to~~ their counter~~-~~parts. In contrast to our finding, the study conducted in India [22] showed that public school students had a higher level of depression than private school students; this disparity may be explained by differences in sample size and method of analysis. Our~~analysis; our~~ study had a larger sample size than the India study,

and the India study did not address the confounding effect. Higher levels of depression in private schools may be due to parents' expectations of high academic achievement in private schools; which leads students to be stressed when they try to meet their parents' expectations, and stress leads to depression.

Our findings showed that~~,~~ holding other predictors constant,~~constants,~~ having a history of physical trauma had a direct positive effect [adjusted β= 0.06, 95% CI: 0.014, 0.125] on anxiety. This indicates that students with physical trauma had higher levels of anxiety than~~as compared with~~ those who did~~had~~ not. Our finding was ~~a~~ supported by the study conducted in the Netherlands~~Netherland~~ [120]. This consistency could be justified by the fact that in reaction to trauma, neurotransmitters such as dopamine, serotonin, and gamma amino butyric acid may be impacted by cortisol~~,~~ and depletion of these~~thus~~ neurotransmitters may lead to anxiety. In addition,~~Besides,~~ individuals with physical trauma may internalize the event and may have subsequent negative thoughts about themselves,~~themselves;~~ which affect their emotional experience and~~, which~~ might lead~~s~~ to anxiety [121].

As the level of depression increases the level of somatic symptoms will also increase [adjusted β= 0.96, 95% CI= 0.167, 3.629]. In the same way, as the level of anxiety increases the level of somatic symptoms also increases [adjusted β=0.66, 95% CI= 0.270, 3.825]. This result is supported by~~with~~ other studies [66, 76, 77] [66, 74, 75]. A possible~~Possible~~ explanation for this consistency could be that in depressed individuals' neurotransmitters such as~~like~~ nor-epinephrine and serotonin are low; a decrease~~low, decrement~~ in neurotransmitters may lead individuals to focus on physical symptoms. Moreover, it could be explained by the co-occurrence of somatic symptoms, anxiety, and depression. The sharing~~Sharing~~ of common sets of gene and environmental factors may also be a~~the~~ possible justification.

Strengths and limitations

A bidirectional~~Bidirectional~~ relationship between anxiety and depression was~~were~~ assessed. In addition, the~~Besides~~ direct and indirect effects of different independent variables on different dependent variables were assessed simultaneously. However, this study is not without limitations.~~limitation.~~ First, only school adolescents in Northwest Ethiopia were included in the study which may affect generalization to all adolescents in Ethiopia. Second, self-reported screening tools were used, which may overestimate the effect size. Third, we only asked about alcohol intake once, not how much, how frequently, or for how long, which could influence the outcome. Last, the~~Lastly,~~ temporal relationship between predictors and outcomes was not evaluated due to the cross-sectional nature of the data.

Conclusion

In this study, a moderate prevalence of depression, anxiety~~,~~ and somatic symptoms was~~were~~ found. Self-rated academic ability, physical trauma, school type, sex, stress, ever use of alcohol, perceived social support, death of a loved one, and having medically confirmed chronic illness were significant determinants of depression, anxiety~~,~~ and somatic symptoms.~~ The bidirectional~~ Bidirectional relationship between anxiety and depression was significant. Depression and anxiety were significant determinants of somatic symptoms. Based on the study findings, the authors suggest the following points: Policy~~-~~makers should strengthen the prevention and management of adolescent~~adolescence~~ mental health disorders. It is better to initiate and implement school-based mental health screening services, particularly for female students and students with low academic ability by educational offices and schools. It is important to emphasize the two-way relationship between anxiety and depression,~~depression;~~

and somatic symptoms should be suspected in individuals with depression and anxiety. Studies including out-–of-–school adolescents are recommended and it is better to assess temporal relationships by using follow-–up studies.

Abbreviations

AIC: Akaike information Criteria, OVB: Omitted variable bias, CFI: Comparative Fix index, CMB: Common Method Bias, OVB: Omitted Variable Bias, PHQ-9A: Patent Health Questionnaire for Adolescent, RMSEA: Rooted Mean Square Error of Approximation, TLI: Tucker Lewis Index,

Acknowledgments

The authors are grateful to the University of Gondar, data collectors, supervisors, and study participants.

Authors' contribution

Conceptualization: Zenebe Abebe Gebreegziabher

Data curation: Zenebe Abebe Gebreegziabher. Ayenew Molla, Rediet Eristu

Formal analysis: Zenebe Abebe Gebreegziabher

Investigation: Zenebe Abebe Gebreegziabher. Ayenew Molla, Rediet Eristu

Methodology: Zenebe Abebe Gebreegziabher. Ayenew Molla, Rediet Eristu

Project administration: Zenebe Abebe Gebreegziabher. Ayenew Molla, Rediet Eristu

Resource: Zenebe Abebe Gebreegziabher. Ayenew Molla, Rediet Eristu

Software: Zenebe Abebe Gebreegziabher, Ayenew Molla, Rediet Eristu

Supervision: Zenebe Abebe Gebreegziabher, Ayenew Molla, Rediet Eristu

Validation: Zenebe Abebe Gebreegziabher, Ayenew Molla, Rediet Eristu

Visualization: Zenebe Abebe Gebreegziabher

Writing:~~—~~ original draft: Zenebe Abebe Gebreegziabher

Writing:~~—~~ review and editing: Zenebe Abebe Gebreegziabher. Ayenew Molla, Rediet Eristu

Supporting Information’~~s~~

S1 Table 1: Summary of key variables in the study

S1 Table~~-~~2: Intraclass correlation coefficients for each outcome variable~~s~~

S1 Table~~-~~3: Instrumental variable validity and strength

S1 Table~~-~~4: Depression among adolescents in Gondar town, 2022

S1 Table~~1~~ 5: Anxiety among adolescents in Gondar town, 2022

S1 Table~~-~~6: Somatic symptoms among adolescents in Gondar town, 2022

S1 Table 7**:** Factorial average item parceling for depression, somatic symptoms, and their determinants among adolescents in Gondar town, 2022

S2 Fig 1: Hypothetical measurement model for depression, somatic symptoms, and their determinants among adolescents in Gondar town, 2022

S3 annex 1: Results of the pilot study

S4 Table: Tools to assess anxiety, depression, somatic symptoms, and their determinants among adolescents in Gondar town, 2022

S5 Data set: Data set of adolescents in Gondar town, 2022

References

1. World Health Organization, *The global burden of disease: 2004 update*. 2008: World Health Organization.

2. Organization, W.H., *Mental health status of adolescents in South-East Asia: Evidence for action.* 2017.

3. Jörns-Presentati, A., et al., *The prevalence of mental health problems in sub-Saharan adolescents: A systematic review*. Plos one, 2021. **16**(5): p. e0251689.

4. Armocida, B., et al., *Burden of non-communicable diseases among adolescents aged 10–24 years in the EU, 1990–2019: a systematic analysis of the Global Burden of Diseases Study 2019.* The Lancet Child & Adolescent Health, 2022.

5. WHO, *Adolescents mental health.* 2019.

6. Ryan, G., O. Qureshi, and N. Salaria, *Mental health and the 2030 Sustainable Development Agenda: Global~~global~~ inaction on mental health is putting the brakes on development.* London, UK: London School of Hygiene and Tropical Medicine, 2018.

7. Kassebaum, N.J., et al., *Global, regional, and national disability-adjusted life-years (DALYs) for 315 diseases and injuries and healthy life expectancy (HALE), 1990–2015: a systematic analysis for the Global Burden of Disease Study 2015.* The Lancet, 2016. **388**(10053): p. 1603-1658.

8. Sayers, J., *The World Health Report~~world health report~~ 2001-Mental health: new understanding, new hope.* Bulletin of the World Health Organization, 2001. **79**: p. 1085-1085.

9. Organization, W.H., *Depression and other common mental disorders: global health estimates*. 2017, World Health Organization.

10. Perez, D.L., et al., *A neural circuit framework for somatosensory amplification in somatoform disorders*. J Neuropsychiatry Clin Neurosci, 2015. **27**(1): p. e40-50.

11. WHO. *depression definition* 2019; Available from: https://www.who.int/news-room/fact-sheets/detail/adolescent-mental-health.

12. WHO., *Adolescents: health risks and solutions. Geneva: World Health Organization.* 2018.

13. Tang, X., et al., *Prevalence of depressive symptoms among adolescents in secondary school in mainland China: A systematic review and meta-analysis*. J Affect Disord, 2019. **245**: p. 498-507.

14. El-Missiry, A., et al., *Screening for depression in a sample of Egyptian secondary school female students*. J Affect Disord, 2012. **136**(1-2): p. e61-e68.

15. Erskine, H.E., et al., *The global coverage of prevalence data for mental disorders in children and adolescents.* Epidemiol Psychiatr Sci, 2017. **26**(4): p. 395-402.

16. Osborn, T.L., et al., *Depression and Anxiety Symptoms, Social Support, and Demographic Factors Among Kenyan High School Students.* Journal of Child and Family Studies, 2020. **29**(5): p. 1432-1443.

17. Girma, S., et al., *Depression and its determinants among adolescents in Jimma town, Southwest Ethiopia.* PLoS One, 2021. **16**(5): p. e0250927.

18. Tirfeneh, E. and M. Srahbzu, *Depression and its association with parental neglect among adolescents at governmental high schools of Aksum town, Tigray, Ethiopia, 2019: a cross-~~-~~sectional study.* Depression research and treatment, 2020. **2020**.

19. Trivedi, D., et al., *Depression among adolescent students in South India: How serious is the issue?* Indian Journal of Psychiatry, 2016. **58**(3): p. 349-350.

20. Raheel, H., *Depression and associated factors among adolescent females in Riyadh, Kingdom of Saudi Arabia, a cross-sectional study.* International journal of preventive medicine, 2015. **6**.

21. Rehm, J. and K.D. Shield, *Global burden of disease and the impact of mental and addictive disorders.* Current Psychiatry Reports,~~psychiatry reports,~~ 2019. **21**(2): p. 1-7.

22. Kumar, R.K., et al., *The prevalence of depression, anxiety, and stress among high school adolescent's children in public and private schools in Rangareddy district Telangana state: A cross-sectional study.* Journal of Education and Health Promotion, 2022. **11**(1): p. 83.

23. BP, C., *Stress, anxiety, and depression among adolescent students of public schools in Kathmandu.* Journal of Institute of Medicine, 2018. **40**(3).

24. Nguyen, D.T., et al., *Depression, anxiety, and suicidal ideation among Vietnamese secondary school students and proposed solutions: a cross-sectional study*. BMC Public Health,~~public health,~~ 2013. **13**(1): p. 1-10.

25. Addy, N.D., et al., *Mental health difficulties, coping mechanisms and support systems among school-going adolescents in Ghana: A mixed-methods study*. PLoS One,~~one,~~ 2021. **16**(4): p. e0250424.

26. Deb, S., E. Strodl, and H. Sun, *Academic stress, parental pressure, anxiety, and mental health among Indian high school students*. International Journal of Psychology and Behavioral Science, 2015. **5**(1): p. 26-34.

27. Galaif, E.R., et al., *Suicidality, depression, and alcohol use among adolescents: a review of empirical findings*. International journal of adolescent medicine and health, 2007. **19**(1): p. 27-36.

28. Huang, R., et al., *Reported alcohol drinking and mental health problems in Hong Kong Chinese adolescents*. Drug and alcohol dependence, 2016. **164**: p. 47-54.

29. Shah, S.M., et al., *Self-esteem and other risk factors for depressive symptoms among adolescents in the United Arab Emirates*. PloS one, 2020. **15**(1): p. e0227483.

30. Gülsen, A. and B. Uygur, *Psychological Features of Smokers*. Respir Care, 2018. **63**(12): p. 1492-1497.

31. Ibrahim, N., et al., *Prevalence and predictors of depression and suicidal ideation among adolescents attending government secondary schools in Malaysia*. Medical Journal of Malaysia, 2017. **72**(4): p. 221-227.

32. Ghobadzadeh, M., et al., *Relationships between adolescent stress, depressive symptoms, and sexual risk behavior in young adulthood: A structural equation modeling analysis.* Journal of Pediatric Health Care, 2019. **33**(4): p. 394-403.

33. Lindholdt, L., et al., *Perceived stress among adolescents as a marker for future mental disorders: A prospective cohort study.* Scandinavian Journal of Public Health, 2021: p. 1403494821993719.

34. Cao, C., et al., *Anxiety, depression, and PTSD symptoms among high school students in china in response to the COVID-19 pandemic and lockdown.* Journal of Affective Disorders, 2022. **296**: p. 126-129.

35. AlAzzam, M., et al., *Predictors of depression and anxiety among senior high school students during COVID-19 pandemic: The context of home quarantine and online education.* The Journal of School Nursing, 2021. **37**(4): p. 241-248.

36. Wittchen, H.-U., C.A. Essau, and J.-C. Krieg, *Anxiety disorders: similarities and differences of comorbidity in treated and untreated groups.* The British Journal of Psychiatry, 1991. **159**(S12): p. 23-33.

37. Essau, C.A., J. Conradt, and F. Petermann, *Frequency, comorbidity, and psychosocial impairment of anxiety disorders in German adolescents.* Journal of anxiety disorders, 2000. **14**(3): p. 263-279.

38. Chang, C.-W., R. Yuan, and J.-K. Chen, *Social support and depression among Chinese adolescents: The mediating roles of self-esteem and self-efficacy.* Children and Youth Services Review, 2018. **88**: p. 128-134.

39. Bandelow, B. and S. Michaelis, *Epidemiology of anxiety disorders in the 21st century.* Dialogues in clinical neuroscience, 2022.

40. Beesdo, K., et al., *Incidence and risk patterns of anxiety and depressive disorders and categorization of generalized anxiety disorder*. Archives of General Psychiatry,general psychiatry, 2010. **67**(1): p. 47-57.

41. Yang, X., et al., *Global, regional and national burden of anxiety disorders from 1990 to 2019: results from the Global Burden of Disease Study 2019.* Epidemiology and Psychiatric Sciences, 2021. **30**: p. e36.

42. Vos, T., et al., *Global burden of 369 diseases and injuries in 204 countries and territories, 1990–2019: a systematic analysis for the Global Burden of Disease Study 2019.* The Lancet, 2020. **396**(10258): p. 1204-1222.

43. Kessler, R.C., et al., *Twelve- month and lifetime prevalence and lifetime morbid risk of anxiety and mood disorders in the United States*. International journal of methods in psychiatric research, 2012. **21**(3): p. 169-184.

44. Biswas, T., et al., *Global variation in the prevalence of suicidal ideation, anxiety, and their correlates among adolescents: a population-—based study of 82 countries.* EClinicalMedicine, 2020. **24**: p. 100395.

45. Malak, M.Z. and A.H. Khalifeh, *Anxiety and depression among school students in Jordan: Prevalence, risk factors, and predictors.* Perspectives in psychiatric care, 2018. **54**(2): p. 242-250.

46. Sandal, R.K., et al., *Prevalence of depression, anxiety and stress among school going adolescent in Chandigarh.* Journal of family medicine and primary care, 2017. **6**(2): p. 405.

47. Bifftu, B. and Y. Guracho, *A Systematic Review and Meta-Analysis of Anxiety among Children and Youth in Ethiopia.* J Depress Anxiety Disorder,~~Disord,~~ 2021. **3**(2): p. 89-100.

48. Lancet, T., *Better understanding of youth mental health*. 2017. p. 1670.

49. Tramonte, L. and D. Willms, *The prevalence of anxiety among middle and secondary school students in Canada.* Canadian Journal of Public Health/Revue Canadienne de Sante'e Publique, 2010: p. S19-S22.

50. Smith, L., et al., *Correlates of symptoms of anxiety and depression and mental wellbeing associated with COVID-19: a cross-sectional study of UK-based respondents.* Psychiatry Res, 2020. **291**: p. 113138.

51. Syokwaa, K.A., P.J. Aloka, and S.N.F. Ndunge, *The relationship between anxiety levels and academic achievement among students in selected secondary schools in Lang'ata District, Kenya.* Journal of Educational and Social Research, 2014. **4**(3): p. 403-403.

52. Khanum, F. and S. Razack, *Anxiety–Herbal treatment: A review.* Res Rev Biomed Biotech, 2010. **1**(2): p. 83-89.

53. Kumari, A. and J. Jain, *Examination stress and anxiety: A study of college students.* Global Journal of Multidisciplinary Studies, 2014. **4**(1): p. 31-40.

54. Bekhuis, E., et al., *Differential associations of specific depressive and anxiety disorders with somatic symptoms.* Journal of Psychosomatic Research,~~psychosomatic research,~~ 2015. **78**(2): p. 116-122.

55. Rice, F., M.B.M. van den Bree, and A. Thapar, *A population-based study of anxiety as a precursor for depression in childhood and adolescence.* BMC Psychiatry, 2004. **4**(1): p. 43.

56. Eriksen, T.E. and M.B. Risør, *What is called symptom?* Med Health Care Philos, 2014. **17**(1): p. 89-102.

57. Edgcomb, J.B., C.H. Tseng, and B. Kerner, *Medically unexplained somatic symptoms and bipolar spectrum disorders: A systematic review and meta-analysis.* J Affect Disord, 2016. **204**: p. 205-13.

58. Tomenson, B., et al., *Total somatic symptom score as a predictor of health outcome in somatic symptom disorders.* Br J Psychiatry, 2013. **203**(5): p. 373-80.

59. Löwe, B., et al., *Depression, anxiety and somatization in primary care: syndrome overlap and functional impairment.* General hospital psychiatry, 2008. **30**(3): p. 191-199.

60. Kroenke, K. and A.D. Mangelsdorff, *Common symptoms in ambulatory care: Incidence, evaluation, therapy, and outcome.* The American Journal of Medicine, 1989. **86**(3): p. 262-266.

61. Kurlansik, S.L. and M.S. Maffei, *Somatic symptom disorder.* American family physician, 2016. **93**(1): p. 49-54.

62. Martin, J.C.S., et al., *Somatic complaints and symptoms of anxiety and depression in a school-based sample of preadolescents and early adolescents. Functional impairment and implications for treatment.* Journal of Cognitive and Behavioral Psychotherapies, 2011. **11**(2): p. 191-208.

63. Carlsson, J., *Prevalence of headache in schoolchildren: relation to family and school factors.* Acta Paediatr, 1996. **85**(6): p. 692-6.

64. Murray, A.M., et al., *Barriers to the diagnosis of somatoform disorders in primary care: protocol for a systematic review of the current status.* Systematic reviews, 2013. **2**(1): p. 1-8.

65. Karaca, S., et al., *Somatic Symptoms in Secondary School Students and Parental Attitudes*. Journal of Psychiatric Nursing/Psikiyatri Hemsireleri Dernegi, 2015. **6**(3).

66. Haraldstad, K. and T.H. Stea, *Associations between pain, self-efficacy, sleep duration, and symptoms of depression in adolescents: a cross-sectional survey*. BMC Public Health,~~public health,~~ 2021. **21**(1): p. 1-7.

67. Larsson, B.S., *Somatic complaints and their relationship to depressive symptoms in Swedish adolescents*. Journal of Child Psychology and Psychiatry, 1991. **32**(5): p. 821-832.

68. Heidari, Z., et al., *Demographic and lifestyles~~Life Styles~~ Determinants of Somatic Complaints' Structures: A Cross-sectional Study on a Large Sample of Iranian Adults Using Factor Mixture Model*. Int J Prev Med, 2017. **8**: p. 8.

69. Ruotsalainen, H., et al., *Systematic review of physical activity and exercise interventions on body mass indices, subsequent physical activity, and psychological symptoms in overweight and obese adolescents*. Journal of Advanced Nursing,~~advanced nursing,~~ 2015. **71**(11): p. 2461-2477.

70. Noh, J.-W., et al., *Relationships between Extra-School Tutoring Time, Somatic Symptoms, and Sleep Duration of Adolescent Students: A Panel Analysis Using Data from the Korean Children and Youth Panel Survey*. International Journal of Environmental Research and Public Health, 2020. **17**(21): p. 8037.

71. Glise, K., G. Ahlborg, and I.H. Jonsdottir, *Prevalence and course of somatic symptoms in patients with stress-related exhaustion: does sex or age matter*. BMC Psychiatry,~~psychiatry,~~ 2014. **14**(1): p. 1-13.

72. Garralda, M.E., *Unexplained physical complaints.* Pediatric Clinics, 2011. **58**(4): p. 803-813.

73. Glise, K., G. Ahlborg, Jr., and I.H. Jonsdottir, *Prevalence and course of somatic symptoms in patients with stress-related exhaustion: does sex or age matter*. BMC Psychiatry,~~psychiatry,~~ 2014. **14**: p~~p~~. 118-118.

74. van Boven, K., et al., *Do unexplained symptoms predict anxiety or depression? Ten-year data from a practice-based research network.* British Journal of General Practice, 2011. **61**(587): p. e316-e325.

75. Kingery, J.N., G.S. Ginsburg, and C.A. Alfano, *Somatic symptoms and anxiety among African American adolescents.* Journal of Black Psychology, 2007. **33**(4): p. 363-378.

76. Janssens, K.A., et al., *Anxiety and depression are risk factors rather than consequences of functional somatic symptoms in a general population of adolescents: the TRAILS study*. Journal of Child Psychology and Psychiatry, 2010. **51**(3): p. 304-312.

77. Knook, L.M., et al., *Psychiatric disorders in children and adolescents presenting with unexplained chronic pain: what is the prevalence and clinical relevancy?* European child & adolescent psychiatry, 2011. **20**(1): p. 39-48.

78. Abdulghani, H.M., et al., *Stress and its effects on medical students: a cross-sectional study at a college of medicine in Saudi Arabia.* Journal of health, population, and nutrition, 2011. **29**(5): p. 516.

79. Organization, W.H., *World health statistics overview 2019: Monitoring~~monitoring~~ health for the SDGs, sustainable development goals*. 2019, World Health Organization.

80. Erskine, H., et al., *The global coverage of prevalence data for mental disorders in children and adolescents.* Epidemiology and psychiatric sciences, 2017. **26**(4): p. 395-402.

81. Dadi, A.F., *The mental health consequences of war in northern Ethiopia: why we should be concerned.* The Lancet Psychiatry, 2022. **9**(3): p. 194-195.

82. report, G.c.a., *total population estimation report.* Gondar City Administrative Office,~~city adminstrative office,~~ 2022.

83. Bentler, P.M. and C.-P. Chou, *Practical issues in structural modeling.* Sociological methods & research, 1987. **16**(1): p. 78-117.

84. Kroenke, K., R.L. Spitzer, and J.B. Williams, *The PHQ‐9: validity of a brief depression severity measure.* Journal of General Internal Medicine,~~general internal medicine,~~ 2001. **16**(9): p. 606-613.

85. Spitzer, R.L., et al., *A brief measure for assessing generalized anxiety disorder: the GAD-7.* Archives of internal medicine, 2006. **166**(10): p. 1092-1097.

86. Gierk, B., et al., *The somatic symptom scale–8 (SSS-8): a brief measure of somatic symptom burden.* JAMA Internal Medicine,~~internal medicine,~~ 2014. **174**(3): p. 399-407.

87. Cohen, S., T. Kamarck, and R. Mermelstein, *A global measure of perceived stress.* Journal of Health~~health~~ and Social Behavior,~~social behavior,~~ 1983: p. 385-396.

88. Bøen, H., O.S. Dalgard, and E. Bjertness, *The importance of social support in the associations between psychological distress and somatic health problems and socio-economic factors among older adults living at home: a cross-~~–~~sectional study.* BMC geriatrics, 2012. **12**(1): p. 1-12.

89. Group, W.A.W., *The alcohol, smoking and substance involvement screening test (ASSIST): development, reliability, and feasibility*. Addiction, 2002. **97**(9): p. 1183-1194.

90. Biddle, S.J., et al., *An assessment of self-reported physical activity instruments in young people for population surveillance: Project ALPHA.* International Journal of Behavioral Nutrition and Physical Activity, 2011. **8**(1): p. 1-9.

91. Shukur, G. and P. Mantalos, *Size and power of the RESET test as applied to systems of equations: a bootstrap approach.* Journal of Modern Applied Statistical Methods,~~modern applied statistical methods,~~ 2004. **3**(2): p. 10.

92. Kline, R.B., *Principles and practice of structural equation modeling*. 2015: Guilford publications.

93. Kumar, S. and S. Kumar, *Structure equation modeling basic assumptions and concepts: A novices guide.* Asian Journal of Management Sciences, 2015. **3**(07): p. 25-28.

94. Roth, P.L. and F.S. Switzer III, *A Monte Carlo analysis of missing data techniques in an HRM setting.* Journal of Management, 1995. **21**(5): p. 1003-1023.

95. Aguirre-Urreta, M.I. and J. Hu, *Detecting common method bias: Performance of ~~the~~ Harman's single-factor test.* ACM SIGMIS Database: The Database~~the DATABASE~~ for Advances in Information Systems, 2019. **50**(2): p. 45-70.

96. Paxton, P., et al., *Nonrecursive models: Endogeneity, reciprocal relationships, and feedback loops*. Vol. 168. 2011: Sage Publications.

97. Technology, E.F.M.o.S.a., *national-research-ethics-review-guidline*. 2014.

98. Steiger, J.H., *Structural model evaluation and modification: An interval estimation approach.* Multivariate behavioral research, 1990. **25**(2): p. 173-180.

99. Bhattarai, D., N. Shrestha, and S. Paudel, *Prevalence and factors associated with depression among higher secondary school adolescents of Pokhara Metropolitan, Nepal: a cross-sectional study*. BMJ Open, 2020. **10**(12): p. e044042.

100. Anjum, A., et al., *Investigating the prevalence of and factors associated with depressive symptoms among urban and semi-urban school adolescents in Bangladesh: a pilot study.* International Health,~~health,~~ 2019.

101. Urmila, K., et al., *Prevalence and risk factors associated with depression among higher secondary school students residing in a boarding school of North Kerala, India.* Int J Contemp Pediatr, 2017. **4**(3): p. 735.

102. Khalid S, A.G., A.A. Hasan S, and M. Ossama A, *Prevalence of depression, anxiety and stress as measured by the depression, anxiety, and stress scale [DASS-42] among secondary school girls in Abha, Saudi Arabia.* 2009.

103. Alharbi, R., et al., *Depression and anxiety among high school student at Qassim Region.* Journal of family medicine and primary care, 2019. **8**(2): p. 504.

104. Widiger, T.A., P.T. Costa, and A.P. Association, *Personality disorders and the five-factor model of personality*. 2013: JSTOR.

105. Bender,~~Bener,~~ A., S. Ghuloum, and F.T. Burgut, *Gender differences in the prevalence of somatoform disorders in patients visiting primary care centers.* Journal of primary care & community health, 2010. **1**(1): p. 37-42.

106. Anjum, A., et al., *Investigating the prevalence of and factors associated with depressive symptoms among urban and semi-urban school adolescents in Bangladesh: a pilot study.* International health, 2022. **14**(4): p. 354-362.

107. Satcher, D., *Mental health: Culture, race, and ethnicity—A supplement to mental health: A report of the surgeon general*. 2001: US Department of Health and Human Services.

108. McEwen, B.S. and T.A. Milner, *Hippocampal formation: shedding light on the influence of sex and stress on the brain.* Brain Res Rev, 2007. **55**(2): p. 343-55.

109. Johnson, D.P. and M.A. Whisman, *Gender differences in rumination: A meta-analysis.* Personality and individual differences, 2013. **55**(4): p. 367-374.

110. Wharton, W., et al., *Neurobiological underpinnings of the estrogen-mood relationship.* Current Psychiatry Reviews,~~psychiatry reviews,~~ 2012. **8**(3): p. 247-256.

111. Narbonne,~~Narbone,~~ M., et al., *Hormonal dysregulation~~disregulation~~ and catamenial epilepsy*. Functional Neurology, 1990. **5**(1): p. 49-53.

112. Huang, R., et al., *Reported alcohol drinking and mental health problems in Hong Kong Chinese adolescents.* Drug and Alcohol Dependence, 2016. **164**: p. 47-54.

113. Deykin, E.Y., J.C. Levy, and V. Wells, *Adolescent depression, alcohol and drug abuse.* American Journal of Public Health, 1987. **77**(2): p. 178-182.

114. Miller, J.W., et al., *Binge drinking and associated health risk behaviors among high school students.* Pediatrics, 2007. **119**(1): p. 76-85.

115. Guerri, C. and M. Pascual, *Mechanisms involved in the neurotoxic, cognitive, and neurobehavioral effects of alcohol consumption during adolescence.* Alcohol, 2010. **44**(1): p. 15-26.

116. Yousefi, F., et al., *The relationship between gender, age, depression and academic achievement.* 2010.

117. Demuthova, S., *The School Environment as a Source of Somatic Problems in Adolescents*. Revista Românească pentru Educaţie Multidimensională, 2019. **11**(2): p. 59-71.

118. El-Anzi, F.O., *Academic achievement and its relationship with anxiety, self-esteem, optimism, and pessimism in Kuwaiti students*. Social Behavior and Personality: Anan international journal, 2005. **33**(1): p. 95-104.

119. Li, Y., et al., *Social support, attachment closeness, and self-esteem affect depression in international students in China*. Frontiers in Psychology, 2021. **12**: p. 618105.

120. Hovens, J.G., et al., *Childhood life events and childhood trauma in adult patients with depressive, anxiety and comorbid disorders vs. controls*. Acta Psychiatrica Scandinavica,psychiatrica scandinavica, 2010. **122**(1): p. 66-74.

121. Association, A.P., *DSM 5 Diagnosticdiagnostic and statistical manual of mental disorders*, in *DSM 5 Diagnostic and statistical manual of mental disorders*. 2013. p. 947 p.-947 p.

Response to Editor and Reviewers

1. **Response to chief academic editor**

Manuscript title: Determinants of adolescent's depression, anxiety, and somatic symptoms in Northwest Ethiopia: A Non-recursive structural equation modeling.

Manuscript ID: PONE-D-23-02244R2

Authors response: Dear Editors and Reviewers, Thank you for giving us the opportunity to submit a revised version of the manuscript "Determinants of adolescent's depression, anxiety, and somatic symptoms in Northwest Ethiopia: A Non-recursive structural equation modeling." for publication in the PLOS ONE journal. We appreciate the time and effort you and the reviewers put into providing feedback on our article, and we appreciate the insightful remarks and useful advice you gave, which considerably improved the manuscript. We have taken into account the reviewers' suggestions. Track modifications are used to highlight these changes in the revised document.

Journal Requirements:

Please review your reference list to ensure that it is complete and correct. If you have cited papers that have been retracted, please include the rationale for doing so in the manuscript text, or remove these references and replace them with relevant current references. Any changes to the reference list should be mentioned in the rebuttal letter that accompanies your revised manuscript. If you need to cite a retracted article, indicate the article's retracted status in the References list and also include a citation and full reference for the retraction notice.

Authors' response: The editor's use of full insight to check for the inclusion of retracted paper in our reference list is really appreciated. We reviewed our references, and there is no reference list of works that have been retracted in our manuscript.

Additional Editor Comments (if provided):

Editor: Please address reviewer's comment as advised.

Authors: Dear Chief Academic Editor, Thank you for assisting us in emphasizing the reviewer's comment. We attempted to answer the reviewer's comments one by one.

Reviewers' comments:

Reviewer's Responses to Questions

Comments to the Author

1. If the authors have adequately addressed your comments raised in a previous round of review and you feel that this manuscript is now acceptable for publication, you may indicate that here to bypass the “Comments to the Author” section, enter your conflict of interest statement in the “Confidential to Editor” section, and submit your "Accept" recommendation.

Reviewer #1: All comments have been addressed

Reviewer #2: All comments have been addressed

2. Is the manuscript technically sound, and do the data support the conclusions?

The manuscript must describe a technically sound piece of scientific research with data that supports the conclusions. Experiments must have been conducted rigorously, with appropriate controls, replication, and sample sizes. The conclusions must be drawn appropriately based on the data presented.

Reviewer #1: Yes

Reviewer #2: Yes

3. Has the statistical analysis been performed appropriately and rigorously?

Reviewer #1: Yes

Reviewer #2: Yes

4. Have the authors made all data underlying the findings in their manuscript fully available?

The PLOS Data policy requires authors to make all data underlying the findings described in their manuscript fully available without restriction, with rare exception (please refer to the Data Availability Statement in the manuscript PDF file). The data should be provided as part of the

manuscript or its supporting information, or deposited to a public repository. For example, in addition to summary statistics, the data points behind means, medians and variance measures should be available. If there are restrictions on publicly sharing data—e.g. participant privacy or use of data from a third party—those must be specified.

Reviewer #1: Yes

Reviewer #2: Yes

5. Is the manuscript presented in an intelligible fashion and written in Standard English?
PLOS ONE does not copyedit accepted manuscripts, so the language in submitted articles must be clear, correct, and unambiguous. Any typographical or grammatical errors should be corrected at revision, so please note any specific errors here.

Reviewer #1: Yes

Reviewer #2: No

6. Review Comments to the Author

Please use the space provided to explain your answers to the questions above. You may also include additional comments for the author, including concerns about dual publication, research ethics, or publication ethics. (Please upload your review as an attachment if it exceeds 20,000 characters)

Reviewer #1: (No Response)

Reviewer #2: In general the paper is greatly strengthened and I appreciate the close attention to the reviewers' comments and the changes made to the manuscript. My main remaining comments are .

2. Response to reviewer 2

Author responses: Dear Dr. Tracy Solomon, first and foremost, we would want to express our gratitude for your tremendous effort, dedication, humility, and time spent commenting on our paper. Following that, we'd like to respond to the comments point by point.

Reviewer #2: 1. As another reviewer/editor has pointed out, the occurrence of anxiety, depression and somatic symptoms in adolescence is not a new hypothesis or finding. The unique contribution of the paper is documenting these issues in an Ethiopian adolescents and the exploration of potential contributing factors in that population. Hence, I would start the paper by making this point before moving on to define anxiety, depression (I would use this order) and somatic disorders, their prevalence in other regions, other parts of Africa and then Ethiopia, perhaps pointing out the dearth of research on Ethiopian adolescents in the conclusion of this section of the introduction.

Authors' response: Dear Dr. Tracy Solomon, we are grateful for pointing out this. The paper's distinctive contribution is not only that it documents the issue in Ethiopian teenagers, but it also addresses the indirect effect of predictors, and we attempted to incorporate what distinguishes adolescents in northwest Ethiopia.Kindly look at line number 108-114 in the revised manuscript without track changes. We prefer the sequence of depression, anxiety, and somatic symptoms (DASS) in terms of severity.

Reviewer #2. Efforts at writing in English which may not be the native language of the authors is always admirable, in my view, and should not prohibit the publishing of important and interesting data as we have here. However, for maximum impact, it would make sense for the authors to have a reviewer read the paper for smooth flow in the English language as a final polish before submitting. In general, the paper is clear but this is scientific writing and precision in language counts. No criticism of the authors, just a suggestion to "professionalize" the writing further before being considered for acceptance. I wish the authors the best of luck in getting the paper accepted for publication.

Authors' response: Dear Dr. Tracy Solomon, thank you for pointing out this issue. All authors evaluated the text several times; senior writers were consulted, and internet tools such as Grammarly (https://app.grammarly.com/ddocs/2167802892) were used in the updated manuscript to make the paper clearer.

Thanks again

On behalf of the authors,

Zenebe Abebe
