# Supplementary material for: Determinants of adolescents’ depression, anxiety, and somatic symptoms in Northwest Ethiopia: A non-recursive structural equation modeling
Source: PLoS One. 2024 Apr 10;19(4):e0281571. doi: 10.1371/journal.pone.0281571 (PMC11006201; doi:10.1371/journal.pone.0281571)
Supplement: Attachment — Submitted filename: Review of PONE-D-23-02244.docx [file pone.0281571.s013.docx]

**Review of PONE-D-23-02244**

The authors are to be congratulated for this much needed work on an important topic and with a population for which there is currently limited information in the literature. The reviewer notes as well that the project was unfunded, hence the project is laudable.

The study was well-executed, the paper is reasonably well-written overall and the study findings ought to be brought to light. However, the papers requires considerable effort in improving the expression and particularly the organization of ideas in order to be considered for publication.

Below I list some general comments for improvement, followed by numerous specific recommendations. Note that my thinking evolved over reading the paper and writing up these comments, so it’s a good idea to read them in their entirety, before making changes. Apologies in advance for comments out of order, I do not have time to edit these suggestions.

General Recommendations

It would be much easier to read and review if you used the usual conventions for manuscript preparation e.g. the use subtitles/sections such as Introduction (which does not require a subtitle) Methods (with subsections Study Design and Context, Participants [including all details regarding sampling], Measures/Instruments (choose one and see note below about how to organize), Results (with subsection “Plan for Analysis” then report the actual analysis and results) and finally Discussion. Please see for example, the APA version 7 for how to prepare the document (although you would want to use numeric referencing as required by PLoSONE).

The following link may be helpful.

<https://www.apa.org/pubs/journals/resources/manuscript-submission-guidelines>

Using the conventional section for a manuscript will help to avoid redundancy or making reference to things not yet described, which occurs fairly frequently in the paper.

Please also note that indenting each new paragraph makes the manuscript much easier to read, especially to review.

Another main consideration here is the paper organization – this applies to all aspects of the paper (though much less so the introduction, which is sensibly written) but even more

to the methods, results and discussion. The reporting of measures, results and discussion are far to piece-meal and list-like. It makes it very hard for the reader to digest or to grasp the big picture of the findings, let alone to make sense of them.

I understand this is primarily an exploratory paper (no apparent hypotheses are being tested) but suggest the authors consider 2-3 primary predictors and then perhaps others that are secondary. Report the results (and also address in the discussion) the primary predictors first looking at impact on all outcomes (depression, anxiety and somatic illness) simultaneously, not in different paragraphs. Then summarize these findings (in the discussion, discuss them). Then concisely report the picture for the secondary predictors, also looking at impact on all outcomes simultaneously, followed by a brief summary.

Readers can consult the tables for the different outcomes if they want the specific findings for each predictor and outcome combination.

In other words, the results and discussion need to be better and more tightly organized. The authors need to consider what are the most important findings to share, to paint the big picture and how it fits into the rest of the literature, then organize the paper to highlight these findings.

Specific Recommendations

**Title:**

Depression, Anxiety, Somatic symptom and their determinants among High School

and Preparatory School Adolescents in Gondar Town, Northwest Ethiopia, 2022.Nonrecursive

Structural Equation Modeling.

**Suggested Revised Title:**

Determinants of adolescent depression, anxiety, somatic symptoms in Northwest Ethiopia.

**Abstract**

In general (throughout the paper), care should be taken to guard the anonymity and confidentiality of the participating sample. See e.g. the suggested revised title. In the abstract, 2^nd^ paragraph “…to 1407 adolescents in northwest Ethiopia”.

Please remove all names of particular schools and keep description to the region where the study was conducted, the two different types of schools and how they differ and keep sample demographics and so on in Participants section.

Comment to remove the names of particular schools applies throughout the paper.

Results section in the abstract:

It would be better to organize and to make more concise the results reported. Perhaps according to outcomes? Determinants of anxiety, then of depression, then interrelated findings for these variables.

**Introduction (Does not need to be labeled “Introduction”)**

At line 68, please place a comma after “…adolescents, is characterized…”

Lines 70-71 Suggest move the sentence on the impact of depression combined with anxiety to the end of the next para on anxiety.

Line 76, I think you mean “threats” and not “treats”

Line 90 “..think or experience feelings about symptoms…”

Line 101-102, the sentence is not complete. Please attach sentences starting at 101 and 104 to the para above.

Line 104-106 What is the nature of the relation? Positive or negative?

Line 113-114 Suggest, “Finally, individuals with… may experience physical harm from unnecessary medical procedures”

Line 135 Not clear here what you mean by “as an input toward sustainable development”.

**Methods (Include this major subtitle)**

Line 138 Suggest “Study Design and Context” as first subsection.

Sample Description – Label as Participants

Please justify the use of this particular population of Ethiopian adolescents. How might this contribute to or constrain generalization of study findings? Is this a convenience sample?

Line 149, suggest remove the section on population as this information is given elsewhere.

Lines 162-169 please correct the use of parentheses, 1 set with commas between different types of free parameters). Currently, very hard to follow.

Line 171, if the sampling was stratified by the 2 different types of schools, say so here or above under context. If stratified first by type of school and then grade, at this point please include “(see below)” otherwise the comment here about a 2-stage sampling procedure seems out of place.

Line 183, Under sampling procedure, I would not name the particular schools involved in the study as this poses a risk to participant confidentiality. Knowing the names of particular schools does not enhance the study reporting.

Line 201, please place these variables in a table, move them to supplementary text. Place a footnote or in the text refer the reader to the table for a summary of the key variables in the study.

Line 216, suggest remove the section on “Operational definitions” and instead place the revelant information in the next section, immediately after you describe the measurement instruments. So after describing the depression measure very briefly, then say the criteria you used for categorization in the study.

Line 245 Suggested “self-reported questionnaire”.

Please be sure that in the sample description you indicate the percentage/proportion/number of sighted and sight challenged individuals and proportion by school type and gender perhaps?

Sight capabilities of participants should not suddenly appear here.

Clarify if the questionnaires were administered in paper and pencil format or on a computer or other device.

249-251, I do not understand the use of “The tool for this study here”. Do you mean “Questionnaires were designed to gather data on…” (insert your list of domains here).

Suggest place all of your measures in a table with columns for 1. Instrument name and citation 2. Brief description and 3. Criteria for categorization you included above.

Line 268, there should be a subheading for results.

Line 271, does the translation apply to all of the questionnaires (were they arranged into one long document?)

Although the Data Quality and Management efforts are laudable, this section is long and superfluous here.

Suggest, remove lines 269 to first part of 271, you already mentioned trained data collectors above. Great that you checked face validity, construct validity and reliability. For the latter to please indicate very briefly how you checked these.

On second thought; perhaps you coud have a “Measures” section, where you include the table suggested above. Before the table, include all information related to the measures such as translation and also efforts to get at validity and reliability. Include a footnote there and describe the pilot study briefly in the footnote. What was done? What did you find? Or place the pilot study in supplementary materials and refer to it in the paper. Note, it is not clear in what sense the pilot study constituted “participatory” research. Needs to be clarified wherever you put the pilot study description.

Lines 279-286 Suggest remove the rest of this paragraph. What does it mean to say “After data entry, data recoding, and missing value management were considered accordingly.” If important, it could go at the start of the Results section, perhaps right before “Plan for Analysis”.

Are the Results given on page 14 for the pilot study? If yes, suggest the entire pilot study goes in Supplementary Material. Simply say in the manuscript that you did a pilot study to further investigate the validity of the questionnaire. Then refer the reader to Supplementary Material.

The pilot study takes up a lot of space in the manuscript but, if I understand correctly, it is meant to convey background information to support the use of the questionnaire in the main study. The manuscript body should focus on the main study.

Remove the sentence end of line 320 to 321. Connect the sentence at line 34 to the previous paragraph.

**Results?**

The results section is generally far too long, with too many explanatory details about how structural equation modelling works. If you aim to show your thoroughness in carefully undertaking this analysis, only mention the specific ways in which you did this in the paper. Assume the audience knows something about SEM.

I will leave suggestions for how to make this section more focused and concise to reviewers with greater expertise than mine in SEM.

Suggest re-ordering reporting of results to follow same order as mentioned in introduction and as the order of measures described in the methods section – depression, anxiety, somatic symptoms, then interrelated of these.

It is difficult the follow the list of findings in the paragraphs for each of these outcomes. Could you organize into direct effects only, unpack these, then indirect effects, upack these and then perhaps deal with variables with both direct and indirect effects on the outcome that is the focus of that paragraph (depression, anxiety or somatic symptoms).

Line 677. “…Bangladesh use a cutoff above five…” please add “whereas in the current study, we used a cutoff on the X measure of Y”. Fill in X and Y accordingly.

Interesting point. Please elaborate briefly on how socio-cultural factors might help to account for discrepancies in findings here.

Line 683, It could also be that females are more likely to report on their depression!

Cultures vary in terms of how males and females ought to deal with things such as depression and this might have an impact on how willing or unwilling males and females are report being depressed or having other mental health challenges.

Line 689, regarding the following sentence:

“Besides, adolescent girls are more likely to have negative life events in relation to their parents and peers, and females are more emotion focused and have a distracting coping style than males”

Please unpack these findings so the reviewer can understand exactly what the authors of the cited paper found. It is not clear to me why adolescent girls are more likely to have negative life events in relation to their parents and peers. What does it mean to be “more emotion focused and to have a distracting coping style than males”?

Line 699 – 700, these claims need supporting references.

Line 701, it is also possible that people who are more depressed are more likely to use alcohol as a coping mechanism.

Line 707. It could also be that being depressed means you have low motivation to study or even that you do not see any point in striving in school given low or no expectations regarding a well-adjusted, productive or successful future.

In reporting results, it would be group according to outcome e.g. depression. Then review of the variables with a direct effect, briefly unpack, those with indirect effects, briefly unpack and then with both direct and indirect impact, then describe – in other words follow the same list and organization suggested for reporting results.

Listing and unpacking the individual findings is difficult to follow – focus on the overall pattern, the big picture and why those findings might have obtained.

Losing a loved one is a traumatic and by definition a sad experience. This is not a surprising finding and does not seem to require much more unpacking. Respondents could still be recovering from this loss.

Line 743 and 744, interesting finding re: private school and higher rates of depression but in the results you report both direct (negative) and indirect (positive) with a small net positive effect. It’s worth referring to this and elaborating briefly here.

Line 777-778, if you are going to suggest that cultural differences might account for inconsistencies in results across studies, good to give an example of exactly how this might play out.

Lines 786-788, “…students with high self-rated academic ability will be more

likely to make decisions, express their feelings without feeling embarrassed” Doing better academically is likely to increase confidence compared to doing poorly and these might lead to more confidence in decision making but it is not clear to me why this would increase the willingness to express feelings without being embarrassed and necessarily to more optimism.

Lines 799-805, again it could be that women are more likely to disclose struggles with anxiety compared to men.

The point above about organizing study findings in the results and discussion applies to all of the outcomes. Another way to organize might be to treat each predictor in turn and then consider the impact on all three outcomes. There were some common findings for predictors with different outcomes e.g. alcohol consumption and private school both had a positive relationship with depression and also with anxiety. The explanations for the impact on different outcomes might be the same, so would only need to be mentioned once.

Study Strengths and Limitations

Generalizability is also constrained by the specific, targeted sample. The authors should recognize and address this point. Is the sample nonetheless diverse and representative of Ethiopian adolescents and if yes, how do you know this and what then does this mean for study generalizability but also for policy and practice in Education, in Ethiopian society?

In several places in the discussion, the authors raise the point of cultural differences and how these might account for discrepancies in study findings across studies conducted in different cultures. But they never really expand on the nature of those differences. Yet this would be so interesting here and contribute to the literature on factors that contribute to mental health outcomes and their context specificity or universality, which has knock on effects for how to mitigate these struggles or how to approach treatment.

Indeed, I suggest a paragraph/section at least in the discussion on the role of context in the present study and connection to other similar studies in different cultures.

It would be better if the recommendations were not stated in bullet form.

Line 987 and on

To avoid the possibility of stigma surrounding coping with and addressing mental health issues, preventative and coping strategies at every level might be better if offered to everyone, not targeting those with certain characteristics highlighted by the study findings. Targeted treatment might invoke stigma which could actually negatively impact mental health.

Line 991, see suggestion above

Line 999, I do not think you established that drinking alcohol caused mental health issues. If this is a coping mechanism or addiction is in play, it might be better to seek treatment for the mental health issues as alcohol consumption is likely to come up as part of this. Parental sudden restriction but exacerbate symptoms without expert treatment.

Line 1001, again alcohol consumption might be a coping mechanism or indeed a symptom of struggles rather than causally related. Not as well that you only asked about “ever consumption” not about how much or how frequently or for how many years consumed (another study limitation).

Line 1006, interesting suggestion regarding out-of-school adolescents. It would be good to provide prevalence data – these might be indicators of struggles with mental health in themselves.
