# Supplementary material for: Determinants of adolescents’ depression, anxiety, and somatic symptoms in Northwest Ethiopia: A non-recursive structural equation modeling
Source: PLoS One. 2024 Apr 10;19(4):e0281571. doi: 10.1371/journal.pone.0281571 (PMC11006201; doi:10.1371/journal.pone.0281571)
Supplement: Attachment — Submitted filename: Response to Reviewers.docx [file pone.0281571.s014.docx]

**Rebuttal letter that responds to each point raised by the academic editor and reviewers**

| Comment given by | Parts of Comment | Comments | Response by Author |
| --- | --- | --- | --- |
| By editors | General | Please ensure that your manuscript meets PLOS ONE's style requirements, including those for file name  In your Data Availability statement, you have not specified where the minimal data set underlying the results described in your manuscript can be found. PLOS defines a study's minimal data set as the underlying data used to reach the conclusions drawn in the manuscript and any additional data required to replicate the reported study findings in their entirety. All PLOS journals require that the minimal data set be made fully available. For more information about our data policy, please see <http://journals.plos.org/plosone/s/data-availability>. Upon re-submitting your revised manuscript, please upload your study’s minimal underlying data set as either Supporting Information files or to a stable, public repository and include the relevant URLs, DOIs, or accession numbers within your revised cover letter.  Please review your reference list to ensure that it is complete and correct. If you have cited papers that have been retracted, please include the rationale for doing so in the manuscript text, or remove these references and replace them with relevant current references. Any changes to the reference list should be mentioned in the rebuttal letter that accompanies your revised manuscript. If you need to cite a retracted article, indicate the article’s retracted status in the References list and also include a citation and full reference for the retraction notice | Accepted and corrected  Accepted and included in supplementary file  No change made |
| Reviewer #1 | General comment | - Was there no exclusion criteria used - Do you consider the person with PHQ-9A score of less than 9 have no depression? A person with a PHQ-9A score of 5-9 has mild depression. Do you think missing these segments of the population has effect on your result? - Assed’: Good to write in full assessed on line 955 | Note changed : Night preparatory and high school students and students who were transferred in from other areas after January 8, 2022 were excluded and it were already addressed in line number 155,156 and 157   - No change made, since PHQ-9A score above 9 had equal sensitivity and specificity DSMI-V criteria. Score above 5 is less sensitive and PHQ-9 score ≥10 had optimal sensitivity (88%) and a specificity ( 88%) - Accepted and corrected. |
| Reviewer #2: | General recommendation | It would be much easier to read and review if you used the usual conventions for manuscript preparation e.g. the use subtitles/sections such as Introduction (which does not require a subtitle) Methods (with subsections Study Design and Context, Participants [including all details regarding sampling], Measures/Instruments (choose one and see note below about how to organize), Results (with subsection “Plan for Analysis” then report the actual analysis and results) and finally Discussion. Please see for example, the APA version 7 for how to prepare the document (although you would want to use numeric referencing as required by PLoSONE).  Using the conventional section for a manuscript will help to avoid redundancy or making reference to things not yet described, which occurs fairly frequently in the paper.  Please also note that indenting each new paragraph makes the manuscript much easier to read, especially to review.  Another main consideration here is the paper organization – this applies to all aspects of the paper (though much less so the introduction, which is sensibly written) but even more to the methods, results and discussion. The reporting of measures, results and discussion are far to piece-meal and list-like. It makes it very hard for the reader to digest or to grasp the big picture of the findings, let alone to make sense of them.  I understand this is primarily an exploratory paper (no apparent hypotheses are being tested) but suggest the authors consider 2-3 primary predictors and then perhaps others that are secondary. Report the results (and also address in the discussion) the primary predictors first looking at impact on all outcomes (depression, anxiety and somatic illness) simultaneously, not in different paragraphs. Then summarize these findings (in the discussion, discuss them). Then concisely report the picture for the secondary predictors, also looking at impact on all outcomes simultaneously, followed by a brief summary. Readers can consult the tables for the different outcomes if they want the specific findings for each predictor and outcome combination.  In other words, the results and discussion need to be better and more tightly organized. The authors need to consider what are the most important findings to share, to paint the big picture and how it fits into the rest of the literature, then organize the paper to highlight these findings. | Accepted and corrected.  Accepted and corrected  Accepted and corrected  Accepted and corrected  No change made on the result part: we preferred the second alternative, as stated in the specific comment; unpack the direct effect, then the indirect effect, and finally combined direct and indirect effects. Because it may become more confusing when you write their direct, indirect, and total effect on the three outcomes concurrently  On the discussion part: accepted and corrected  Accepted and corrected |
|  | Title | Depression, Anxiety, Somatic symptom and their determinants among High School  and Preparatory School Adolescents in Gondar Town, Northwest Ethiopia, 2022.Nonrecursive  Structural Equation Modeling.  **Suggested Revised Title:**  Determinants of adolescent depression, anxiety, somatic symptoms in Northwest Ethiopia. | Accepted and corrected |
|  | Abstract | In general (throughout the paper), care should be taken to guard the anonymity and confidentiality of the participating sample. See e.g. the suggested revised title. In the abstract, 2^nd^paragraph “…to 1407 adolescents in northwest Ethiopia”.  Please remove all names of particular schools and keep description to the region where the study was conducted, the two different types of schools and how they differ and keep sample demographics and so on in Participants section.  Comment to remove the names of particular schools applies throughout the paper.  Results section in the abstract:  It would be better to organize and to make more concise the results reported. Perhaps according to outcomes? Determinants of anxiety, then of depression, then interrelated findings for these variables. | Accepted and corrected  Accepted and corrected  Accepted and corrected |
|  | **Introduction** | **Introduction (Does not need to be labeled “Introduction”)** | Accepted and corrected |
|  |  | At line 68, please place a comma after “…adolescents, is characterized…” | Accepted and corrected |
|  |  | Lines 70-71 Suggest move the sentence on the impact of depression combined with anxiety to the end of the next para on anxiety. | Accepted and corrected |
|  |  | Line 76, I think you mean “threats” and not “treats” | Accepted and corrected |
|  |  | Line 90 “..Think or experience feelings about symptoms…” | Accepted and corrected |
|  |  | Line 101-102, the sentence is not complete. Please attach sentences starting at 101 and 104 to the para above. | Accepted and corrected |
|  |  | Line 104-106 What is the nature of the relation? Positive or negative? | Accepted and corrected |
|  |  | Line 113-114 Suggest, “Finally, individuals with… may experience physical harm from unnecessary medical procedures” | Accepted and corrected |
|  |  | Line 135 Not clear here what you mean by “as an input toward sustainable development”. | No changed made: in the sustainable development goal their slogan says “no health without mental health” and one of the indicator to achieve their target is research, so this study might have some contribution in achieving that goal. |
|  | Methods | Line 138 Suggest “Study Design and Context” as first subsection. | Accepted and corrected |
|  |  | Please justify the use of this particular population of Ethiopian adolescents. How might this contribute to or constrain generalization of study findings? Is this a convenience sample? | Accepted and corrected |
|  |  | Line 149, suggest remove the section on population as this information is given elsewhere. | Accepted and corrected |
|  |  | Lines 162-169 please correct the use of parentheses, 1 set with commas between different types of free parameters). Currently, very hard to follow. | Accepted and corrected |
|  |  | Line 171, if the sampling was stratified by the 2 different types of schools, say so here or above under context.If stratified first by type of school and then grade, at this point please include “(see below)” otherwise the comment here about a 2-stage sampling procedure seems out of place | Accepted and corrected |
|  |  | Line 183, Under sampling procedure, I would not name the particular schools involved in the study as this poses a risk to participant confidentiality. Knowing the names of particular schools does not enhance the study reporting. | Accepted and corrected |
|  |  | Line 201, please place these variables in a table, move them to supplementary text. Place a footnote or in the text refer the reader to the table for a summary of the key variables in the study. | Accepted and corrected |
|  |  | Line 216, suggest remove the section on “Operational definitions” and instead place the relevant information in the next section, immediately after you describe the measurement instruments. So after describing the depression **measure** very briefly, then say the criteria you used for categorization in the study | Accepted and corrected |
|  |  | Line 245 Suggested “self-reported questionnaire”. | Accepted and corrected |
|  |  | Please be sure that in the sample description you indicate the percentage/proportion/number of sighted and sight challenged individuals and proportion by school type and gender perhaps? Sight capabilities of participants should not suddenly appear here. | Not changed: there are a small number of individuals with sight problems and we mention those individuals to show the fairness of the study but they are to small to describe in proportion and to show their variation with different variables. |
|  |  | Clarify if the questionnaires were administered in paper and pencil format or on a computer or other device. | Accepted and corrected |
|  |  | 249-251, I do not understand the use of “The tool for this study here”. Do you mean “Questionnaires were designed to gather data on…” (insert your list of domains here). | Accepted and corrected |
|  |  | Suggest place all of your measures in a table with columns for 1. Instrument name and citation 2.Brief description and 3. Criteria for categorization you included above. | Accepted and corrected |
|  |  | Line 268, there should be a subheading for results. | Accepted and corrected |
|  |  | Line 271, does the translation apply to all of the questionnaires (were they arranged into one long document?) | Unchanged: yes it was for all questionnaire |
|  |  | Suggest, remove lines 269 to first part of 271, you already mentioned trained data collectors above. Great that you checked face validity, construct validity and reliability. For the latter to please indicate very briefly how you  checked these. | Accepted and corrected |
|  |  | On second thought; perhaps you coud have a “Measures” section, where you include the table suggested above. Before the table, include all information related to the measures such as translation and also efforts to get at validity and reliability. Include a footnote there and describe the pilot study briefly in the footnote. What was done? What did you find? Or place the pilot study in supplementary materials and refer to it in the paper. Note, it is not clear in what sense the pilot study constituted “participatory” research. Needs to be clarified wherever you put the pilot study description. | Accepted and corrected |
|  |  | Are the Results given on page 14 for the pilot study? If yes, suggest the entire pilot study goes in Supplementary Material. Simply say in the manuscript that you did a pilot study to further investigate the validity of the questionnaire. Then refer the reader to Supplementary Material.  The pilot study takes up a lot of space in the manuscript but, if I understand correctly, it is meant to convey background information to support the use of the questionnaire in the main study. The manuscript body should focus on the main study. | Accepted and corrected |
|  |  | On second thought; perhaps you coud have a “Measures” section, where you include the table suggested above. Before the table, include all information related to the measures such as translation and also efforts to get at validity and reliability. Include a footnote there and describe the pilot study briefly in the footnote. What was done? What did you find? Or place the pilot study in supplementary materials and refer to it in the paper. Note, it is not clear in what sense the pilot study constituted “participatory” research. Needs to be clarified wherever you put the pilot study description |  |
|  |  | Lines 279-286 Suggest remove the rest of this paragraph. What does it mean to say “After data entry, data recoding, and missing value management were considered accordingly.” If important, it could go at the start of the Results section, perhaps right before “Plan for Analysis”. | Accepted and corrected |
|  |  | Are the Results given on page 14 for the pilot study? If yes, suggest the entire pilot study goes in Supplementary Material. Simply say in the manuscript that you did a pilot study to further investigate the validity of the questionnaire. Then refer the reader to Supplementary Material.  The pilot study takes up a lot of space in the manuscript but, if I understand correctly, it is meant to convey background information to support the use of the questionnaire in the main study. The manuscript body should focus on the main study. | Accepted and corrected |
|  |  | Remove the sentence end of line 320 to 321. Connect the sentence at line 34 to the previous paragraph. | Accepted and corrected |
|  | Result | The results section is generally far too long, with too many explanatory details about how structural equation modeling works. If you aim to show your thoroughness in carefully undertaking this analysis, only mention the specific ways in which you did this in the paper. Assume the audience knows something about SEM. | Accepted and corrected |
|  |  | Suggest re-ordering reporting of results to follow same order as mentioned in introduction and as the order of measures described in the methods section – depression, anxiety, somatic symptoms, then interrelated of these. | Accepted and corrected |
|  |  | It is difficult the follow the list of findings in the paragraphs for each of these outcomes. Could you organize into direct effects only, unpack these, then indirect effects, upack these and then perhaps deal with variables with both direct and indirect effects on the outcome that is the focus of that paragraph (depression, anxiety or somatic symptoms). | Accepted and corrected |
|  |  | Line 677. “…Bangladesh use a cutoff above five…” please add “whereas in the current study, we used a cutoff on the X measure of Y”. Fill in X and Y accordingly. | Accepted and corrected |
|  |  | Interesting point. Please elaborate briefly on how socio-cultural factors might help to account for discrepancies in findings here. | Accepted and corrected |
|  |  | Line 683, It could also be that females are more likely to report on their depression!  Cultures vary in terms of how males and females ought to deal with things such as depression and this might have an impact on how willing or unwilling males and females are report being depressed or having other mental health challenges. | Accepted and corrected |
|  |  | Line 689, regarding the following sentence:  “Besides, adolescent girls are more likely to have negative life events in relation to their parents and peers, and females are more emotion focused and have a distracting coping style than males” | Accepted and removed |
|  |  | Line 701, it is also possible that people who are more depressed are more likely to use alcohol as a coping mechanism. | Accepted, but not modified; it might be, but you can't prove it in this study because it is cross-sectional. |
|  |  | Line 707. It could also be that being depressed means you have low motivation to study or even that you do not see any point in striving in school given low or no expectations regarding a well-adjusted, productive or successful future. | Accepted, but not altered,. since you can’t assess all direction or relationship by a single study |
|  |  | In reporting results, it would be group according to outcome e.g. depression. Then review of the variables with a direct effect, briefly unpack, those with indirect effects, briefly unpack and then with both direct and indirect impact, then describe – in other words follow the same list and organization suggested for reporting results.  Losing a loved one is a traumatic and by definition a sad experience. This is not a surprising finding and does not seem to require much more unpacking. Respondents could still be recovering from this loss. | Accepted and corrected  Accepted and corrected. |
|  |  | Listing and unpacking the individual findings is difficult to follow – focus on the overall pattern, the big picture and why those findings might have obtained. | Accepted and corrected |
|  |  | Line 777-778, if you are going to suggest that cultural differences might account for inconsistencies in results across studies, good to give an example of exactly how this might play out. | Accepted and justified |
|  | Strength and limitation | Generalizability is also constrained by the specific, targeted sample. The authors should recognize and address this point. Is the sample nonetheless diverse and representative of Ethiopian adolescents and if yes, how do you know this and what then does this mean for study generalizability but also for policy and practice in Education, in Ethiopian society? | Accepted and corrected |
|  |  | In several places in the discussion, the authors raise the point of cultural differences and how these might account for discrepancies in study findings across studies conducted in different cultures. But they never really expand on the nature of those differences. Yet this would be so interesting here and contribute to the literature on factors that contribute to mental health outcomes and their context specificity or universality, which has knock on effects for how to mitigate these struggles or how to approach treatment. | Accepted and modified on the discussion part for the magnitude of depression |
|  |  | It would be better if the recommendations were not stated in bullet form. | Accepted and corrected |
|  |  | To avoid the possibility of stigma surrounding coping with and addressing mental health issues, preventative and coping strategies at every level might be better if offered to everyone, not targeting those with certain characteristics highlighted by the study findings. Targeted treatment might invoke stigma which could actually negatively impact mental health. | Accepted and corrected |
|  |  | Line 999, I do not think you established that drinking alcohol caused mental health issues. If this is a coping mechanism or addiction is in play, it might be better to seek treatment for the mental health issues as alcohol consumption is likely to come up as part of this. Parental sudden restriction but exacerbate symptoms without expert treatment. | Not changed: Yes, you did not demonstrate a link between alcohol consumption and mental illness, but you advise parents to discourage adolescent alcohol consumption rather than abruptly discontinue it. And, according to my reading, alcohol consumption in adolescent age groups is not suggested in any level. |
