# Supplementary material for: Determinants of adolescents’ depression, anxiety, and somatic symptoms in Northwest Ethiopia: A non-recursive structural equation modeling
Source: PLoS One. 2024 Apr 10;19(4):e0281571. doi: 10.1371/journal.pone.0281571 (PMC11006201; doi:10.1371/journal.pone.0281571)
Supplement: Attachment — Submitted filename: Manuscript_edi.docx [file pone.0281571.s015.docx]

**Determinants of adolescent depression, anxiety, and somatic symptoms in Northwest Ethiopia;** **A Non-recursive structural equation modeling.**

Zenebe Abebe Gebreegziabher^1*^, Rediet Eristu^2^, Ayenew Molla^2^

^1^ Department of Epidemiology and Biostatistics, School of Public Health, Asrat Woldeyes Health Science Campus, Debre Birhan University, Debre Birhan, Ethiopia

^2^ Department of Epidemiology and Biostatistics, Institute of Public Health, College of Medicine and Health Sciences, University of Gondar, Gondar, Ethiopia

* Corresponding author

Email: [zenebeabe7054@gmail.com](mailto:zenebeabe7054@gmail.com) (ZA)

# Abstract

**Introduction:** In developing countries including Ethiopia, mental health is not only a public concern; but also a developmental issue. Depression and anxiety are the commonest mental health disorders and often somatic symptoms co-exist with them. Adolescents with common mental health problems are associated with increased risk of suicide, future unemployment, and poor quality of life. Little is known about the mental health of adolescents in the Ethiopia. Thus, this study aimed to assess the determinants of depression, anxiety, and somatic symptoms among adolescent in Northwest Ethiopia, 2022.

**Methods:** Institution based cross-sectional study was conducted from June 8 to 24, 2022. Two-stage stratified random sampling was used to select 1407 adolescent in Northwest Ethiopia. Data were collected through structured and standard self-administered questionnaires Non-recursive structural equation modeling was employed to assess the direct, indirect, and total effects of the predictors. Degree of relationship was interpreted based on adjusted regression coefficients with corresponding 95% confidence interval.

**Results:** Magnitude of anxiety, depression, and somatic symptoms were 25.05% (95%CI: 22.8, 27.5), 28.21 (95% CI: 25.8, 31%), and 25.24(95% CI: 23, 27.6%), respectively. Ever use of alcohol had a positive effect on depression [β= 0.14, 95% CI: 0.073, 0.201], anxiety [adjusted β= 0.11, 95% CI: 0.041, 0.188] and somatic symptom [adjusted β= 0.12, 95% CI: 0.062, 0.211]. Stress had a significant positive effect on depression [adjusted β= 0.76, 95% CI: 0.642, 0.900], anxiety [adjusted β= 1.10, 95% CI: 0.955, 1.264], and somatic symptom [adjusted β= 086, 95% C: 0.700, 1.025]. High level of anxiety was significantly related to higher level of depression [adjusted β= 0.74, 95% CI: 0.483, 1.081]. Depression had also a direct positive effect on anxiety [adjusted β= 0.74, 95% CI: 0.508, 1.010].

**Conclusions and recommendations:** Magnitude of anxiety, depression, and somatic symptoms were moderate. Self-rated academic ability, physical trauma, school type, sex, stress, ever use of alcohol, perceived social support, death of beloved one, and having medically confirmed chronic illness were independent predictors of anxiety, depression and somatic symptoms. The bidirectional relationship between anxiety and depression was significant. Emphasis should be given to the prevention and management of mental health in the adolescent, particularly targeting adolescents with the aforementioned factors.

**Keywords:** anxiety, depression, somatic symptoms, adolescents, structural equation modeling, Ethiopia

# Introduction

Mental health disorder accounts for 16% of global burden of disease and injuries and more than 80% of people with mental health disorders live in low and middle income countries, with increase in incidence and mortality [2].Adolescent are especially vulnerable to developing mental illness[3]. In this population mental health disorders is increased by 32.18% from 1999 to 2019 [4]. World health organization estimates that 10-20% of adolescents are affected by mental health problem in the world[5]. In low- and middle-income countries, adolescents mental health is not only a major public health challenge, but also a development concern[6].

Human suffering and financial costs associated with mental health disorders are substantial and growing globally [7]. Each year more than 12 billion working days are lost due to mental illness. Between 2011-2030 , it is estimated that mental illness will cost the global economy 16 trillion united states dollar in lost economic output more than diabetes, cancer, and respiratory diseases [6]. Depression and anxiety are among the common mental health disorders [8],and often somatic symptom co-exist with them [9, 10].

Depression, which is the second common mental health disorder in adolescents, is characterized by loss of interest or pleasure, feelings of guilt or low self-worth, feelings of tiredness, disturbed sleep or appetite, and poor concentration and sadness [11].In 2018, over one million adolescents died from preventable causes, with depression playing a significant role [13]. In Asia, its magnitude ranges from 24.3% to 57.7% [14] and in Sub-Saharan Africa, it ranges from 15.5% [15] to 45.90% [16, 17]. In Ethiopia, 28% [18] of adolescents in Jimma and 36.2% [19] of adolescents in Aksum were depressed.

Anxiety is characterized by emotional feelings of excessive fear, nervousness, avoiding threats in the environment perceived by them, and physical symptoms such as fast respiration, increased blood pressure, and tightness of the chest [20, 21]. Globally, 301.39 million peoples are affected by anxiety [22]. It is the leading cause of mental disorder in the world, which accounts for about 28.68 million disability-adjusted life years in the global burden of disease [23]. Anxiety is the commonest mental health disorder in adolescents [24], with high magnitude in developed as well as in developing countries[25]. In adolescents, it ranges from 42.1% [26] to 80.85% in Asia [27], and in sub-Saharan Africa 29.8% of adolescents had anxiety [3]. A study conducted in Kenya showed that 37.99% of high school adolescents had anxiety[17]. A systematic review conducted among children and youth in Ethiopia revealed that general anxiety disorder ranges from 0.5-23% [28].In combination with depression , it contributes for 45% of the overall burden of disease [12].

Somatic symptom is characterized by a comprehensive list of symptoms such as pain, breathlessness, numbness, palpitation, tiredness, headache, dizziness, and gastroenterological problems. Somatic symptom disorder is a disorder in which individuals excessively or disproportionally think or experience feeling about the symptoms, which result in significant disturbance in daily life [29].Often depression and anxiety co-exists with somatic symptom [30-32], which is one of the remarkably widespread issues in primary health care and subspecialty settings [10]. Somatic symptoms results in more than half of the patient visit in primary health care [33]. Its magnitude is estimated to be 5 to 7% [34] in general population and 5 to 30 % [35, 36] in adolescent. Studies showed that age [17], female sex[18, 37-39], being in public school [40], high family academic pressure [41, 42], poor self-rated academic ability[43, 44], alcohol use [45, 46], cigarette smoking [47, 48] ,stress, [49-51], somatic symptom[18, 37, 38, 49, 52, 53], and anxiety [54, 55] had a positive relation with depression. While other factors like social support [43, 44, 52, 56] and family education had negative association [53].Literatures reported that female sex[27, 57], age [17, 26], smoking [48, 58], alcohol use , low self-rated academic ability [26, 59], family academic pressure [41],tress [60, 61], somatic symptom disorder [62] and depression [63] had positive relationship with anxiety. Different scholars reported that female sex[64-66] , being in private school [64], physically inactivity [67, 68], extra school tutoring [69] , stress [70-72], anxiety [65, 73, 74] and depression [65, 75, 76] had significant positive relation with somatic symptom.

Somatic symptoms delay the diagnosis of depression and anxiety, because most people seek medical attention for bodily symptoms. Finally, Individuals with somatic symptoms may experience harm from unnecessary medical procedures [84].

Despite the fact that mental health is a key part of the sustainable development goals which affect every other goals [6], the magnitude of mental health disorders is still high [27]. To decrease the burden of mental illness, targeting adolescents is important; since more than half of mental health problems starts at this age [6], and 50% of adolescent mental health problems will proceed to adulthood [85]. Adolescents in low and middle-income countries are disproportionally affected by mental health disorders; however, there are limited data on the prevalence and determinants of mental illness in Sub-Saharan Africa [86, 87].

In Ethiopia, though mental health services were included in the national health policy, there is a paucity of evidence on the magnitude and determinants of anxiety, somatic symptoms, and their relation with depression. Even studies conducted outside of Ethiopia had methodological flaws, as they used logistic or linear regression to assess multiple mental health outcomes, which cannot address the bidirectional relationship between outcome variables and the indirect effects of predictors. In such cases, non-recursive structural equation modeling, which is a multivariate statistical framework used to measure the complex relationships between different observed and latent variables simultaneously is preferable.

Therefore, this study intended to fill this gap by assessing the magnitude, relationship, and determinants of depression, anxiety, and somatic symptoms among high school and preparatory school adolescents in Gondar town by using non-recursive structural equation modeling. The findings from this study will help the psychologist, psychiatrists, policy makers, students, community and the government to tackle the increasing burden of mental health problems. Moreover, it will be used as an input toward sustainable development and as a baseline data for further research.

# Methods and Materials

## Study design, and context

Institution based cross-sectional study was conducted from June 8 -June 24, 2022 among school adolescents in Gondar town, Amhara Region state, northwest, Ethiopia. It is located 728 kilometers from Ethiopia's capital city of Addis Ababa, and 180 kilometers from Bahir Dar, the capital city of the Amhara regional state. There are 395, 000 residents [88], 48 healthcare facilities including one comprehensive specialized hospital, eight health centers, one private general hospital, fifteen specialist clinics, fifteen medium clinics, and eight primary clinics). Adolescent contains more than 25% of Ethiopian population. Adolescence is the transition stage from infancy to adulthood. It includes the major changes in the areas of physical, psychological, social, and cognitive development. This shift is related with role confusion, high levels of stress, and emotional instability [89]. According to the town administrative educational department report, the town has seventeen high and twelve public and five private preparatory schools, with a total number of 24,308 students. Among them, 2408 students were from private schools (1457 girls and 951 boys) and 21900 students were from public schools (12052 girls and 9848 boys).

## Participants

All high and preparatory school adolescents in Gondar town who were registered for the second semester of 2021-2022 academic years were included in the study. Night preparatory and high school students and students who were transferred in from other areas after January 8, 2022 were excluded.

Sample size calculation for structural equation modeling depends on the model complexity. The general rule for sample size calculation in structural equation modeling ranges from 5 to 20 times per free parameters[90]. In this study, 5:1 ratio were used to get the minimum adequate sample size. Based on the hypothesized model *Fig 1*, a total of 134 free parameter were available (***Fig 1****).*

Considering 134 free parameters, the sample size was

134*5=670: Since the sampling procedure is multistage sampling with 2 stages, a design effect of 2 was considered.

Therefore, the sample size becomes 670*2 =1340

After adjusting 5% for non-response rate, the final sample size became

1340+1340*5%=**1407**

*Fig 1: Hypothetical structural equation modeling on the magnitude and determinants of depression, anxiety and somatic symptoms among high school and preparatory school adolescents in Gondar town 2022 (both measurement and structural model):where Circles indicate latent variables or errors terms or disturbances, rectangles indicate observed variables, single arrows indicate factor loadings or regression coefficients, and double arrows indicate the covariance between latent variables.A1-A7=items of anxiety, D1-D9=items for depression, S1-S8= items for somatic symptom, ST1-ST8=items for stress, and =error term.*

In this study, two-stage stratified random sampling technique was used to select participants. A study conducted in Telangana state, India, showed that adolescents in public schools had a higher proportion of depression than those in private school [40].As a result, stratification by school type was done and study subjects were proportionally allocated. Then, two schools from private and four schools from public were selected randomly. After that, heterogeneity by grade level was considered and, stratification was done by grade level (***Fig 2***).

*Fig 2: Diagrammatical representation of the sampling procedure that was used to assess the magnitude and determinants of depression, anxiety, and somatic symptoms among high school and preparatory school adolescents in Gondar town, 2022. Where N= total number of students*

## Variables of the study

### Outcome variables (endogenous constructs):

Depression, anxiety, and somatic symptoms

### Independent variables: summary of key variables used in the study: S1 Table 1

## Data collection procedures and tools

Primary data with pen and pencil format were gathered using structured questionnaire through self-reported questionnaire for those who could see and by face-to-face interviews for 3 individuals who could not see. Four trained data collectors who have a first degree in public health officer were assigned for data collection.

Questionnaire was designed to gather data on socio-demographic characteristics, behavioral factors, academic related factors, and relationship related factors, clinical factors, depression, anxiety, somatic symptom, and stress domains.

Table 1: instruments used for the determinates of depression anxiety, somatic symptom, their description and criteria for categorization

| **Variables** | **Instrument** | **Description** | **Criteria for categorization** | **Reference** |
| --- | --- | --- | --- | --- |
| Depression | PHQ-9A | it is a depression screening tool with 9-items, each item were measured by Likert scale ranging from 0 “not at all” to 3 “nearly every day” and its total score range from 0 to 27 | Students were classified as having depression, if their total score on the Patients Health Questionnaire (PHQ-9A) was 10 or higher | [100] |
| Anxiety | GAS-7 | a brief measure of anxiety that contains seven items which are rated in four point likert scales that range from 0 “not at all” to 3 nearly every day”. Its summed score ranges from 0- 21 | Students were classified as anxious if their total score on the general anxiety scale (GAS-7) was 10 or higher | [92] |
| Somatic symptom | SSS-8 | It had comparable reliability and validity with PHQ-15. Each item was measured by Likert scale ranging from 0 “not at all” to 4”very much’, a total scoring range from minimal 0 to maximum 32 | Students were classified as having 4 to 7 low, 8 to 11 medium, 12 to 15 high, or 16 to 32 very high somatic symptom severity levels | [95] |
| Stress | PSS-10 | Perceived stress scale 10, it contains 10 items. Each item scored on an ordinal scale of 0–4, where 0 represents “never” and 3 represents “very often”. Individual scores range from 0 to 40. | Students who have a total perceived stress Scale 10, scores of 0-13, 14-26, and 27-40 were categorized as having low, moderate, and high perceived stress level respectively | [96] |
| Social support | Oslo social support scale three | It is 3 items with a Likert scale that ranges from 1 to 4 for the first item (number of people you can count if you face a great personal problem) and 1 to 5 ranges for the rest items, its summed score range from 3 to 14. | Students were categorized as having  poor social support 3–8 score , moderate social support 9–11 score, and strong social support with 12–14  score | [97]. |
| Ever use of alcohol | Tool for ever use of alcohol  Tool for current use of alcohol | Do you have a history of alcohol (like beer, Tella, Katikala, Wine) in take in your life time?  Do you drink alcohol within the past 3 months | Respondents who answered yes were considered as positive for ever use of alcohol  Students who answered yes were considered as positive for current alcohol user | [98] |
| Physical activity | Physical activity screening tool for adolescents | In the past one week, how many days did you exercise for at least 60 minutes until we felt sweaty or shortness of breath | Respondents who answered 1 2 days and above were categorized as physically active; and those who answered one day and no was categorized as physically inactive | [97]. |

## Data quality assurance

To maintain the quality of the data, , the questionnaire that developed in English language was translated in to Amharic and re- translated in to English by another person to check consistency. Four public health experts and two psychiatrists checked the face validity of the tool. You did a participatory pilot study to further investigate the reliability and validity of the Questionnaire. Moreover, a pilot s study was conducted among 201 students, 177 from public school and 24 from private school to check the validity and reliability of the tool. Reliability was checked by using Cronbach alpha and the tool was reliable, since the Cronbach alpha values were above 0.7 for both overall and particular factors except for social support. For social support, since it had small number items (<5), average inter-item covariance (AIIC) value was used, thus was 0.4, which in the acceptable range (0.2 to 0.4). The convergent and discriminate validity of the tool was checked. The convergent validity was maintained, since all standardized factor loadings were significant (p value <0.05) and composite reliability was above 0.6. The discriminate validity of the tool was also evidenced, since all inter-factor correlations were less than the square root of AVE for each factor **S3 annex 1**. Pilot study was also used to check the way that each questionnaire were understood by different participants and modifications were done accordingly based on the feedback; since the pilot study was external and participatory.

Additionally in the pilot study Ramsey regression equation specification error test (RESET) was used to check omitted variable bias RESET values above 0.05 indicates the absence of omitted variable bias [104]. In our case, the minimum Ramsey RESET p value was 0.2 **S3 annex 1**. Hence; there was no significant omitted variable bias.

## Data processing, model building and analysis

Collected data were coded and entered in to Epi-data software version 4.6, and exported to STATA version 16 and AMOS version 21 for further analysis. Non-recursive structural equation modeling was employed to assess the complex relationship between different latent and observed variables. Model assumptions such as: specified and identified model, multivariate normality, missing, outlier, sample size adequacy, independency, common method bias and strong and valid instrumental variable were checked.

**Specified model**: Model specification is the first step in structural equation modeling. It is a statement of the theoretical model in terms of equations or a diagram [105]. In this study, we specified the model after extensive literature searching and reading as presented in ***Fig 1***.

**Identified Model**: There are three levels of identification in the structural equation model: under-identified, just identified, and over-identified. To precede with the given model, the model should be over identified or just identified [105]. For non-recursive models, instrumental variables(IV) are mandatory to estimate the parameters [105]. Instrumental variables are methodological devices used to resolve problems of endogeneity; which occurs when an explanatory variable correlates with the error term of the dependent variable. All non-recursive models suffer from endogeneity problems and need IVs. In an equation system as IV→X→Y, IV is any variable that has a strong and significant direct effect on X, but is not correlated with the disturbance term of Y [105].

In this study identification of non-recursive structural equation modeling with complete correlation of error terms was used. There are three ways of identification in non-recursive structural equation modeling; which are: presence of unique instrumental variables, order condition, and rank condition. Among this, only the rank condition is sufficient for identification, and the rest two are necessary, but not sufficient [105]. **A unique instrumental variable:** In this study, all endogenous variables in the reciprocal loop had unique instrumental variables.
**Order condition:** It is a necessary but not sufficient condition for model identification. The number of excluded variables for that specific endogenous variable minus the total number of endogenous variables minus one should be greater than or equal to 0 [105]. In our model, to satisfy order condition, each endogenous variable in the recursive loop should have at least 3-1 =2 excluded variables. Where, 3 is the number of endogenous variables in non-recursive loop. In this study, all endogenous variables in the non-recursive have more than two excluded variables. For depression, anxiety, and somatic symptom disorder, 9, 8, and 11variables were excluded. Therefore, model can full fill requirement of order condition.

**Rank condition**: In this particular study, there are 3 endogenous variables in the non-recursive loop and 17 exogenous variables and one endogenous variable out of the feedback loop (***Fig 1***). Rank Conditions were done using the system matrix. To formulate the system matrix, a separate equation for each endogenous variable in the feedback loop is mandatory. Therefore, the equations for depression, anxiety, and somatic symptom disorder are presented as follows

$\begin{matrix} Depression\left(y1\right)\text{=x}2\text{ alcohol use}+\text{x}3\text{ chronic illness}+\text{x}4\text{ physical activity}+\text{x}5\text{ family mental ilness}+ \\ \text{ x}7\text{ school ty}+\text{x}11\text{ death of beloved one}+\text{x}12\text{ social support}+\text{x}16\text{family pressur} \\ \text{ }+\text{x}18\text{ stress}+\text{x}20\left(\text{y}2\right)+\text{x}21\left(\text{y}3\right)\text{ somaticsymptom} \end{matrix}$ $\begin{matrix} Anxiety\left(y2\right)\text{=x}2\text{ alcohol use}+\text{x}3\text{ chronic illness}+\text{x}4\text{ physical activity}+\text{x}5\text{ family mental ilness}+\text{x}7\text{ school type} \\ \text{ }+\text{x}13\text{ physical trauma}+\text{x}17\text{ acadamic ability}+\text{x}18\text{ stress}+x19\left(y1\right)\text{ }+x21\left(y3\right) \end{matrix}$ $\begin{matrix} \mathbf{Somatic} \mathbf{symptom}\left(\mathbf{y}3\right)\text{=x}2\text{ alcohol use}+\text{x}3\text{ chronic illness}+\text{x}4\text{ physical activity}+\text{x}5\text{ family mental ilness}+\text{x}7 \\ \text{ school type}+\text{x}14\text{ study time}+\text{x}15\text{ extra school tutoring}+\text{x}18\text{ stress}+\text{x}19\left(\text{y}1\right)+\text{x}20\left(\text{y}2\right) \end{matrix}$ Rank conditions in the system matrix below, begins by constructing a system matrix based on the above equations. Endogenous variables are presented in the row and all variables are presented in the column. In each row, zero or one appears in the column that corresponds to that row. One indicates that the variable represented by the column has a direct effect on endogenous variable in that row or on the endogenous variable itself , 0 indicates the excluded variables for that specific endogenous variable in that row [105].

$$\left[\begin{matrix} x & & 2 & 3 & 4 & 5 & 6 & 7 & 8 & 9 & 10 & 11 & 12 & 13 & 14 & 15 & 16 & 17 & 18 & y1 & y2 & y3 \\ y1 & & 1 & 1 & 1 & 1 & 0 & 1 & 0 & 0 & 0 & 1 & 1 & 0 & 0 & 0 & 1 & 0 & 1 & 1 & 1 & 1 \\ y2 & & 1 & 1 & 1 & 1 & 0 & 1 & 0 & 0 & 0 & 0 & 0 & 1 & 0 & 0 & 0 & 1 & 1 & 1 & 1 & 1 \\ y3 & & 1 & 1 & 1 & 1 & 0 & 1 & 0 & 0 & 0 & 0 & 0 & 0 & 1 & 1 & 0 & 0 & 1 & 1 & 1 & 1 \end{matrix}\right]$$

The following steps were followed for identification of rank condition

1. Start with the first row of the system matrix. Cross out all entries in that row and cross out any column in the system matrix with a one in that same row.
2. Simplify the reduced matrix further by deleting any row with all zeros entries; delete any row that is an exact duplicate of another or can be reproduced by adding other rows (i.e., it is a linear combination of other rows). The number of remaining rows is the rank.

If the rank of the reduced matrix is larger than or equal to the sum of the endogenous variables in the recursive loop minus one, then the rank condition is satisfied [105].

The rank condition for this particular study became

For depression (y1), after you cross the entire first row and the entire column which contains 1 in the row:

The reduced matrix becomes:

$\left[\begin{matrix} 1 & 0 \\ 0 & 1 \end{matrix}\right]$Rank=2

For anxiety (y2), after you cross the entire second row and the entire column which contains 1 in that row:

The reduced matrix becomes

$\left[\begin{matrix} 1 & 0 \\ 0 & 1 \end{matrix}\right]$Rank=2

For Somatic symptom (y3), after we cross the entire third row and the entire column which contains one in that row:

Reduced matrix becomes

$\left[\begin{matrix} 1 & 0 \\ 0 & 1 \end{matrix}\right]$Rank=2

The hypothesized model was identified since all endogenous variables have a rank of two, which is equal to the number of endogenous variables in the feedback loop minus one.

**Multivariate normality, outliers, and missing:** Full information maximum likelihood estimation, which assumes multivariate normality for the joint population distribution of the endogenous variables, given the exogenous variables, was used. Multivariate normality was assessed using Mardia’s kurtosis and its critical ratio. Absolute values of 7 for Mardia’s kurtosis and its critical ratio above 5 were used to declare non-normality [105]. The data were not normally distributed. Although item parceling and outlier deletion were tried to handle it, there was no improvement. As a result, bootstrap maximum likelihood estimation with 3000 samples was used.

Mahalanobis distance, which measure the distance relative centroid, was used to check multivariate outliers [105]. Mahalanobis distance p values less than 0.001 based on chi-square distribution were used to declare observation as multivariate outlier [106]. There were 62 observations with Mahalanobis distance p-value less than 0.001. The data were examined to see whether they were actually outliers or caused by data entry errors. However, it was not due to data entry error, and as mentioned above, multivariate normality did not improve significantly with their exclusion. So, those outlier observations were kept in order to maintain the study's power.

From the total observation, there were six observations with missing values. All missing values were from predictors and had no relationship with the outcome variable; thus, list-wise deletion was performed because they constituted less than 5% of the total sample and were considered missing completely at random, so 1373 observations were used for the final analysis[107].

**Sample size adequacy and strength of correlation:** The critical ratios for Bartlett's test of sphericity in this study were high and it was significant for all constructs. KMO was above 0.7 for the overall, as well as for specific constructs. Hence, the sample is sufficient, the population matrix is not identity, and factor analysis was evidenced [105] (**Table 3**).

Table 3*: KMO and Bartlett’s test of sphericity to check sampling adequacy and strength of correlation for the measurement of anxiety , depression, a somatic symptoms among high and preparatory school adolescents in Gondar town, 2022.*

| Factors | KMO | Bartlett’s tests of sphericity | |
| --- | --- | --- | --- |
|  |  | Chi-square | P value |
| Anxiety | 0.88 | 3100 | 0.0001 |
| Depression | 0.87 | 2391 | 0.0001 |
| Somatic | 0.85 | 2155 | 0.0001 |
| Stress | 0.86 | 3354 | 0.0001 |
| Overall | 0.94 | 13938 | 0.0001 |

**Independency of observations**: The intra class correlation coefficients (ICC) were used to test the independence of observations. School type and grade level were employed as clustering variables. ICC was less than 0.1 in our study **S1 Table 2**, and the usual structural equation model was preferred.

**Common Method Bias****:** Harman’s one-factor test was used to check common method bias (CMB).**[108]**. There was no CMB in this investigation since the overall variance recovered by one factor was 25.2%, which is below the suggested cutoff 50%.

**Strong and valid instrumental variables**: Strength and validity of instrumental variables were checked by using Cragg-Donald Wald F statistic and Sargan Hansen test, respectively. Sargan Hansen test P-value greater than 0.05 was used to ponder valid instruments. F test values above 10 were used as a cut of point to declare strong IVs [109]. In the present study, the strength and validity of the instrumental variables between anxiety and somatic symptoms, and between somatic symptoms and depression were invalid and weak. Deleting a non-recursive path is one treatment for a weak and faulty instrument. As a result, the non-recursive paths from somatic symptoms to anxiety and depression were excluded. Finally, identified model with valid and strong instrumental variables in non-recursive loop was preserved **S1 Table 3**.

## Ethical consideration

Ethical approval was obtained from the Institutional Review Board of University of Gondar Institute of public Health. The ethical approval letter was submitted to Gondar town administrative educational department and to all selected high schools and preparatory schools. Permission was obtained from school directors. Parental informed consent was waived as the research had no/ minimal risk [112]. Following permission from the school directors, each student was given a detailed participant information sheet and they completed an assent form to indicate their willingness to participate. Only students who signed an assent form were eligible to take part. Students with moderate to severe anxiety, depression, or somatic symptoms were referred to the University of Gondar Comprehensive specialized hospital psychiatry clinic. To ensure privacy and confidentiality of participants, all information gathered was kept in a locked cabinet in the investigator's home, no personal identifiers were mentioned, and collected data was used solely for research purposes. Overall, this study was carried out in accordance with the Helsinki Declaration.

# Results

## Socio-demographic characteristics and clinical factors of respondents

Out of 1407 randomly selected individuals, 1379 completed the questionnaire; hence, the response rate was 98 %. The mean age of the respondents was 17.23 years with a standard deviation of ±1.25 years. Majority, 1238(89.78%) of the respondents were from public schools and regarding residence two-thirds 915(66.35%) of the respondents were from an urban areas. Around half, 654(47.43%) of the respondents reported moderate stress, while 72 (5.22%) reported high perceived stress. Majority, 1237(89.7%) of the respondents had not a history of chronic illness, and less than one-tenth 101(7.32%) of the respondents had a family history of mental illness **(*Table 4*).**

***Table 4: Socio- demographic and clinical characteristics of high and preparatory school adolescents in Gondar town, 2022 (n=1379)***

| **Variable** | **Frequency** | **Percentage (%)** |
| --- | --- | --- |
| **Sex** | | |
| Male | 536 | 38.87 |
| Female | 843 | 61.13 |
| **School type** | | |
| Public | 1238 | 89.78 |
| Private | 141 | 10.22 |
| **Grade level** | | |
| Grade 9 | 544 | 39.45 |
| Grade 10 | 325 | 23.57 |
| Grade 11 | 296 | 21.46 |
| Grade 12 | 214 | 15.52 |
| **Residence** | | |
| Urban | 915 | 66.35 |
| Rural | 464 | 33.65 |
| **Physical trauma** | | |
| No | 1076 | 78.03 |
| Yes | 303 | 21.97 |
| **Medically confirmed**  **chronic illness** | | |
| No | 1237 | 89.7 |
| Yes | 142 | 10.3 |
| **Stress** | | |
| Low | 653 | 47.35 |
| Moderate | 654 | 47.43 |
| High | 72 | 5.22 |
| **Family history of**  **mental illness** | | |
| No | 1278 | 92.68 |
| Yes | 101 | 7.32 |
| Continuous variable Mean SD | | |
| **Age in year** 17.22 | | ±1.25 |

## Behavioral, academic, and relation related factors

More than one-third, 493(35.75%) of the respondents had a history of alcohol drinking. Regarding physical activity, only 224(16.24%) of the respondents were physically active. Majority, 656(47.57% of the respondents had good self-rated academic ability .When we see perceived social support, two-fifths, 564(40.9%) of the respondents had low perceived social support (***Table 5***).

*Table 5: Behavioral, academic, and relation related factors of high preparatory school adolescents in Gondar town, 2022 (n=1379).*

| **Variables** | **Frequency** | **Percentage (%)** |
| --- | --- | --- |
| **Physical activity** | | |
| Inactive | 1155 | 83.76 |
| Active | 224 | 16.24 |
| **Ever use of alcohol** | | |
| No | 886 | 64.25 |
| Yes | 493 | 35.75 |
| **Extra school tutoring*(n=1378)** | | |
| No | 1011 | 73.3 |
| Yes | 367 | 26.7 |
| **Self-rated academic ability** | | |
| Very poor | 39 | 2.83 |
| Poor | 70 | 5.08 |
| Good | 656 | 47.57 |
| Very good | 390 | 28.28 |
| Excellent | 224 | 16.24 |
| **Father education *(n=1377)** | | |
| Can’t read and write | 266 | 19.29 |
| Grade 1- 8 | 488 | 35.43 |
| Grade 9-12 | 308 | 22.34 |
| Diploma and above | 315 | 22.84 |
| **Mother education* (n=1378)** | | |
| Can’t read and write | 429 | 31.12 |
| Grade 1- 8 | 436 | 31.63 |
| Grade 9-12 | 277 | 20.10 |
| Diploma and above | 236 | 1.13 |
| **Family academic pressure** | | |
| Not at all | 524 | 38 |
| Low | 375 | 27.19 |
| Medium | 144 | 10.44 |
| High | 336 | 24.37 |
| **Death of loved one in the past 6 months** | | |
| No | 1058 | 76.72 |
| Yes | 321 | 23.28 |
| **Social support** |  |  |
| Poor | 564 | 40.9 |
| Medium | 559 | 40.54 |
| Good | 256 | 18.56 |
| **Current alcohol use(n=1377)*** | | |
| No | 368 | 26.73 |
| Yes | 1009 | 73.27 |
| Median  **Study time in hour (n=1379)** 3 | | IQR  ±3 |
| **Time spend for 2**  **extra school tutoring**  in hour(n=367) | | IQR=±2 |

Where,* indicated variables with missing observations

## Magnitude of depression

In this study, the overall magnitude of self-reported depression was 28.21(95% CI= 25.8, 31%)**.** Regarding the level of severity, 34.8% had mild, 18.5% had moderate, 7.72% had moderately severe and 2.04% of respondents had severe depression.More than one-third (34.8%) of participants had a feeling hurting oneself or better off dead. More than two-thirds of the respondents (68.3%) had little interest or pleasure in doing things and more than half of the participants (55.69%) had a feeling of hopelessness**S1 Table 4**.

## Magnitude of Anxiety

The overall magnitude of self-reported anxiety in this study was 25.05% (95%CI: 22.8, 27.5%). Regarding the severity level of anxiety, 34.08% of respondents had mild, 16.82% moderate, and 8.27 % had severe anxiety.Among the total respondents, 16.61% worried too much about different things; nearly every day and 11.54 %could not able to stop worrying too much nearly every day. More than half (58.38%) of the respondents had a feeling of anxious or nervousness **S1 Table 5**.

## Magnitude of somatic symptoms

In this study, the overall magnitude of self-reported somatic symptom disorder was 25.24 % (95% CI= 23, 27.6%). Regarding the level of severity among the total respondents, 27.27 % had mild, 15.88% moderate, 8.19% high, and 6.38% very high somatic symptom respectively. When you see the response to specific indicators, around two-thirds (65.42%) of the participants had headache. More than one half (53.68%) of the respondents had dizziness and 35.97% of the respondent had abdominal pain or gastrointestinal problem **S1 Table 6**.

## Structural equation modeling

Confirmatory factor analysis (CFA) was computed to test the measurement model. The model fit measures (CMIN/DF, CFI, TLI, RMSEA) were used to assess model overall goodness of fit. In the hypothesized measurement model S2 Fig 1, the goodness of fit was below the acceptable level: CMIN/DF=5.10, TLI=, CFI=0.81 and RMSEA=0.05. To improve the fit of the model , factorial item parceling were done [113]S1 Table 7. After that, the model fits were improved. In the final measurement model *Fig3*, all goodness of fit values were in the acceptable level (CMIN/DF=2.60, CFI=1.00, TLI=1.00 and RMSEA=0.00). Construct reliability was assessed by using Cronbach’s alpha and it were above the required level except for social support. For social support, since it had low number item (<5), average inter-item covariance (AIIC) values were used, the value in this study was 0.31, which in the acceptable range (0.20 to 0.40)[102]. Thus, the constructs reliability of the tool was kept up (*Table 6*).

*Fig 3:**Final measurement model for the constructs of anxiety, depression, somatic symptoms, stress, and social support.*

**Key:** a =anxiety, d= depression, SO=somatic symptoms, st=stress, SS= social support, e =error term, and bidirectional arrow indicates covariance

*Table 6: Reliability and model fit for the final measurement model for the constructs of anxiety, depression, somatic symptoms, stress, and social support.*

| Conditions | Cronbach alpha | CMIN/DF | CFI | TLI | RMSEA |
| --- | --- | --- | --- | --- | --- |
| Anxiety | 0.84 | 2.6 | 1.0 | 1.0 | 0.0 |
| Depression | 0.79 |  |  |  |  |
| Somatic symptom | 0.77 |  |  |  |  |
| Stress | 0.80 |  |  |  |  |
| Social support | 0.46 |  |  |  |  |
| Over all | **0.90** |  |  |  |  |

For the model containing the structural and measurement parts, model comparisons were done using AIC, CMIN/DF, TLI, CFI, and RMSEA. Model with only significant variables with significant disturbance after parcel; which had small AIC, small CMIN/DF, and RMSEA, and large CFI and TLI was selected as a good fitted model (***Table 7***).

*Table 7: Model selection for a model containing both measurement and structural parts for anxiety, depression, and somatic symptoms among high and preparatory school adolescents in Gondar town, 2022.*

| Models | AIC | CMIN/DF | CFI | TLI | RMSEA | Remark |
| --- | --- | --- | --- | --- | --- | --- |
| Model with all predictors and covariance after parcel | 2468 | 6.8 | 0.8 | 0.7 | 0.1 |  |
| Model with only significant variables with significant disturbance after parcel | 951 | 3.00 | 0.94 | 0.92 | 0.027 | **selected** |

## Factors related with anxiety, depression and somatic symptom

Self-rated academic ability, perceived social support, physical trauma, death of a loved one, ever use of alcohol, having medically confirmed chronic illness, sex, family pressure, school type, and one endogenous variable stress were all significantly related to anxiety, depression, and somatic symptoms, either directly or indirectly. There was a significant bidirectional relationship between anxiety and depression (***Fig 4*, *Table 8*, *Table 9*, *Table 10***).

***Fig 4: Standardized SEM for factors related with anxiety, depression, and somatic symptom among high and preparatory school adolescents in Gondar town, Northwest, Ethiopia, 2022.***

### Factors related with depression among high and preparatory school adolescents in Gondar town, 2022.

Perceived social support, having a history of death of a loved one within the past six months and anxiety had significant direct effects on depression. While, having medically confirmed chronic illness, having a history of alcohol use, perceived family academic pressure, sex, stress, self-rated academic ability, having history of physical trauma, and depression itself had a statistically significant indirect relationship with depression. School type and anxiety were significantly related to depression both directly as well as indirectly

Anxiety [adjusted β= 0.74, 95% CI: 0.483, 1.081 and Perceived social support [adjusted β= -0.13, 95% CI: -0.229,-0.029] has a positive direct effect on depression. However, experiencing a history of death of beloved one within the past six months had a direct positive effect [adjusted β= 0.03, 95% CI: 0.014, 0.256] on depression. Having a medically confirmed chronic illness had a significant indirect effect on depression [adjusted β = 0.17, 95% CI: 0.085, 0.265]. Self-rated academic ability had a significant indirect effects [adjusted β=-0.05, 95% CI: -0.083,-0.031] on depression. Having a history of physical trauma had a significant indirect effect [adjusted β= 0.10, 95% CI: 0.037, 0.166] on depression. (***Table 9***).

*Table 9: direct, indirect and total effects of socio-demographic, behavioral, relationship related factors, stress and anxiety on depression among adolescents in Gondar town, Northwest Ethiopia, 2022:* ***Unstandardized estimate.***

| Variables | Direct effect | Indirect effect | Total effect |
| --- | --- | --- | --- |
| **DV: Depression** | Estimate (95%CI) | Estimate(95% CI) | Estimate (95%CI) |
| Social support | -0.13[-0.229,-0.029 ]* | ---- | -0.13[-0.229,-0.029 ]* |
| Death of loved one | 0.03[0.004, 0.087 ]* | ----- | 0.03[0.004, 0.087 ]* |
| Self-rated academic ability | ---- | -0.05[-0.083, -0.031]* | -0.05[-0.083, -0.031]* |
| Physical trauma | ---- | 0.10[0.037, 0.166 ]* | 0.10[0.037, 0.166 ]* |
| Ever use of alcohol | 0.06[-0.004, 0.112] | 0.08[0.029, 0.139 ]* | 0.14[0.073, 0.201]* |
| History of chronic illness | 0.04[-0.053, 0.124] | 0.17[0.085, 0.265 ]* | 0.21[0.114, 0.311]* |
| Sex | -0.03[-0.099, 0.025] | 0.09[ 0.042,0.162 ]* | 0.06[0.003, 0.109]* |
| School type | -0.09[-0.194, -0.004]* | 0.20[0.113, 0.320] * | 0.11[0.016 , 0.207]* |
| Family pressure | 0.02[-0.004, 0.040 ] | 0.02[0.002, 0.043 ]* | 0.04[0.016, 0.060]* |
| Stress | -0.06[-0,454, 0.247] | 0.82[0.530, 1.250 ]* | 0.76[0.642, 0.900 ]* |
| Anxiety | 0.74[0.483, 1.081*] |  | 0.74[0.483, 1.081*] |

*Indicates CI did not include a null value (β=0), DV= dependent variable, SE= standard error, CI=confidence interval

### Factors related to anxiety among high and preparatory school adolescents

Self-rated academic ability and physical trauma had direct effects on anxiety. Ever use of alcohol, and having medically confirmed chronic illness had significant indirect effects on anxiety. School type, sex, stress, and depression had statistically significant direct and indirect relationships with anxiety.

Depression had positive direct effect [adjusted β= 0.74, 95% CI: 0.508, 1.010] on anxiety. Self-rated academic ability had a direct negative effect [adjusted β= -0.03, 95% CI: -0.065, -0.006] on anxiety. Having a history of physical trauma had positive effect [adjusted β= 0.06, 95% CI: 0.014, 0.125] on anxiety. Perceived social support had a significant negative indirect effect [adjusted β= -0.22, 95% CI: -.304, -0.139] on anxiety, while having a history of death of a loved one in the past 6 months had a significant positive indirect effect [adjusted β= 0.06, 95% CI: 0.010, 0.122]. Female sex had a significant direct [adjusted β = 0.08, 95% CI: 0.019, 0.135] and indirect [adjusted β = 0.04, 95% CI: 0.004, 0.092] positive effect on anxiety with a total positive effect of 0.12 [adjusted β = 0.12, 95% CI: 0.059, 0.179]. Being in private school had direct [adjusted β= 0.12, 95% CI: 0.017, 0.226] and indirect [adjusted β= 0.16, 95% CI: 0.056, 0.269] positive effect on anxiety, leading to a total positive effect of 0.28 [adjusted β= 0.28, 95% CI: 0.159, 0.415]. Stress had both direct [adjusted β= 0.54, 95% CI: 0.293, 0.745] and indirect [adjusted β= 0.57, 95% CI: 0.379, 0.814] positive effect on anxiety, bringing a total positive effect of 1.10[adjusted β= 1.10, 95% CI: 0.955, 1.264] (*Table 8*).

*Table 8: Direct, indirect and total effects of socio-demographic, behavioral, relationship related factors, stress and depression on anxiety among adolescents in Gondar town, Northwest Ethiopia, 2022****: Unstandardized estimate.***

| **Variables** | Direct effect | Indirect effect | Total effect |
| --- | --- | --- | --- |
| **DV: Anxiety** | Estimate (95% CI) | Estimate (95%CI) | Estimate (95% CI) |
| Self-rated academic ability | -0.03[-0.065, 0.006 ] * | ---- | -0.03[-0.065, 0.006 ]* |
| Physical trauma | 0.06[0.014, 0.125]* | ---- | 0.06[0.014, 0.125] * |
| Social support | ---- | -0.22[-0.304,-0.139 ] * | -0.22[-0.304,-0.139 ]* |
| Death of loved one | ---- | 0.06[0.010, 0.122] * | 0.06[0.010, 0.122 ]* |
| Ever use of alcohol | -0.03[0.088, 0.035] | 0.14[0.071, 0.213 ] * | 0.11[0.041, 0.188] * |
| History of chronic illness | -0.02[-0.124, 0.078] | 0.27[0.165, 0.384] * | 0.25[0.132 , 0.371] * |
| Sex | 0.08[0.019, 0.135 ] * | 0.04[0.004, 0.092] * | 0.12[0.059, 0.179 ] * |
| School type | 0.12[0.017, 0.226 ] * | 0.16[0.056, 0.269] * | 0.28[0.159, 0.415] * |
| Family academic pressure | ---- | 0.03[0.012, 0.050 ] * | 0.03[0.001, 0.50 ] * |
| Stress | 0.54[0.293, 0.745] * | 0.57[0.379, 0.814 ] * | 1.10[0.955, 1.264 ]* |
| Depression | 0.74[0.508, 1.010] * | ---- | 0.74[0.508, 1.010] * |

### *Indicates CI did not include a null value (β=0), DV= dependent variable, SE= standard error, CI=confidence interval

### Factors related to somatic symptoms among high and preparatory school adolescents

Self-rated academic ability, perceived social support, physical trauma, and death of a loved one in the past 6 months had a significant indirect effects on somatic symptoms. While, having a medically confirmed chronic illness, sex, stress, and depression were all directly and indirectly related to somatic symptoms..

Social support [adjusted = -0.11,% CI: -0.176, -0.067] and self-rated academic ability [adjusted =-0.02, 95%CI: -0.039, -010] had a significant negative indirect effect on somatic symptoms, whereas a history of death of a loved one within the previous six months [adjusted = 0.03% CI: 0.005, 0.067] and having history of physical trauma [adjusted β= 0.04, 95% CI:0.012, 0.083] had significant positive indirect effect on somatic symptom. Having medically confirmed chronic illness had significant direct [adjusted β= 0.17, 95% CI= 0.045, 0.290] and indirect [adjusted β= 0.20, 95% CI: 0.122, 0.289] effect on somatic symptoms, bringing a total positive effects of 0.37 [adjusted β= 0.37, 95% CI: 0.239, 0.505]. Being female had statistically significant direct [adjusted β= 0.10, 95% CI: 0.050, 0.166] and indirect [adjusted β= 0.03, 95% CI: 0.002, 0.053] positive effect on somatic symptoms, resulting in a total positive effects of 0.13 [adjusted β= 0.13, 95% CI: 0.074, 0.198]. Stress had both direct [adjusted β= 0.53, 95% CI: 0.326, 0.768] and indirect [adjusted β= 0.33, 95% CI: 0.186, 0.472] positive effect on somatic symptoms resulting in a total positive effect of 0.86 [adjusted β= 086, 95% CI: 0.700, 1.025]. Depression had significant direct [adjusted β= 0.38, 95% CI= 0.167, 0.540] and indirect [adjusted β= 0.58, 95% CI: 0. 0.167, 3.629] positive effect on somatic symptoms resulting in a total positive effect of 0.96 [adjusted β= 0.96, 95% CI: 0.433, 1.456]..(***Table 10***).

*Table 10: direct, indirect and total effects of socio-demographic, personal, relationship related factors, stress, anxiety, and depression on somatic symptom disorders among adolescents in Gondar town, Northwest Ethiopia, 2022****: Unstandardized estimate****.*

| **Variables** | **Direct effect** | **Indirect effect** | **Total effect** |
| --- | --- | --- | --- |
| **DV: Somatic symptom** | Estimate (95%) | Estimate (95%) | Estimate (95%) |
| Self-rated academic ability | ---- | -0.02[-0.039, -010]* | -0.02[-0.039, -010]* |
| Physical trauma | ---- | 0.04[0.012, 0.083 ]* | 0.04[0.012, 0.083]* |
| Social support | ---- | -0.11[-0.176, -0.067]* | -0.11[-0.176, -0.067]* |
| Death of loved one with in the  Past 6 months | ---- | 0.03[0.005, 0.067 ]* | 0.03[0.005, 0.067 ]* |
| Ever use of alcohol | 0.03[-0.022, 0.105] | 0.09[0.044, 0.144 ]* | 0.12[0.062, 0.211]* |
| History of medically confirmed chronic illness | 0.17[0.045, 0.290]* | 0.20[0.122, 0.289]* | 0.37[0.239 , 0.505 ]* |
| Sex | 0.11[ 0.050, 0.166]* | 0.03[0.002, 0.053]* | 0.14[0.074, 0.198 ]* |
| School type | 0.03[-0.074, 0.144 ] | 0.13[0.053, 0.216]* | 0.16[0.046, 0.292]* |
| Stress | 0.53[0.326, 0.768]* | 0.33[0.186, 0.472 ]* | 0.86[0.700, 1.025]* |
| Depression | 0.38[0.167, 0.540]* | 0.58[0.167, 3.629 ]* | 0.96[0.433, 3.774 ]* |
| Anxiety | 0.06[-0.101, 0.212] | 0.60[0.243, 3.825]* | 0.66[0.270, 3.825]* |

*Indicates CI did not include a null value (β=0), DV= dependent variable, SE= standard error, CI=confidence interval

# Discussion

In this study, the magnitude and determinants of anxiety, depression, and somatic symptoms were examined using non-recursive structural equation modeling. Magnitude of depression, anxiety, and somatic symptom were 28.21 [95% CI: 25.8, 31%], 35.82 [(95% CI: 33.3, 38.4%) and 25.24[95% CI: 23, 27.6%] respectively. Self-rated academic ability, physical trauma, school type, sex, stress, ever use of alcohol, perceived social support, death of a loved one, and having medically confirmed chronic illness were independent predictors of anxiety, depression and somatic symptoms. The bidirectional relationship between anxiety and depression was significant. Depression and anxiety were significant predictors of somatic symptom.

**Magnitude of depression, anxiety and somatic symptoms**

In the current study, the overall prevalence of self-reported depression was 28.21(95% CI= 25.8%, 31%). This is in line with the study conducted in Jimma 28% [18] , but it was higher than meta-analysis conducted in china 24.3% [14], and lower than the study conducted in Aksum 38.2% [19], Nepal 44.2% [114], Bangladesh 36%[115] , and India 57.7%[116]. The reason for this discrepancy may be due to differences in the screening tools used. Furthermore, since mental health is the combination of physical physical , social, cultural and religious environment this could be also explained by socio-cultural differences in different settings.Poverty, violence, and other stressful social conditions are not unique to any one region of the world, nor are the symptoms and manifestations that result from them. However, factors that are frequently associated with race or ethnicity, such as socioeconomic position or country of origin, can enhance the likelihood of being exposed to these sorts of stressors and thus stressors may leads to depression[117] .

In this study, the prevalence of anxiety was 25.05% (95%CI: 22.8, 27.5%). It is consistent with the study conducted among children’s and youth in Ethiopia 0.5 to23% [28]. However, this finding is lower than the study conducted in Kenya 37.99% [17], Saudi Arabia 63.5% to 66% [118, 119], in Jordan 42.1% [26] , and in Chandigarh 80.85% [27]. In contrast to this, it was higher than the worldwide estimates of anxiety among children and adolescents (6.5 to 10 %) [120], and among the study conducted in 82 countries, which reported 13-14% in Africa, 9-10% in America, 8-9% in Europe, and 7-8% in Asia [25]. Such variation in the prevalence of anxiety across different continents could originate from variations in the anxiety screening tools used. A study conducted in Saudi Arabia used depression anxiety stress scale (DASS-42, a study conducted in Jordan used symptom checklists anxiety scale, a study conducted in Chandigarh used depression anxiety stress scale-21. Besides, it could also be justified by the different in the cutoff point used: a study conducted in Saudi Arabia used GAS- above five. Thus, variation may also be explained by deference in population characteristics, a study conducted in 82 countries community based study which may decrease the magnitude. Furthermore, cultural differences in different parts of the world may lead to such variations.

In the current analysis, the prevalence of self-reported somatic symptom disorder was 25.24 % (95% CI= 23, 27.6%). This is consistent with the study conducted among children and adolescents in Tarragona which reported 5 to 30 % [35]. The finding in the present study was higher than the magnitude in the general population 5 to 7% [121] and lower than study conducted in Qatar, which reported 47.8% in female and 52.2% in male [122]. The possible explanation for this could be due to variation in the screening tools. The study conducted in Qatar used General Health Questionnaire-12. The study among the general population of children and adolescents used a child somatization inventory. Furthermore, it could also be a variation in population, the study conducted in Qatar was conducted among patients and the study among the general population of children and adolescents including non-school adolescents. Moreover, socio-cultural variation could also be the possible justification.

**Factors related with depression, anxiety and somatic symptom**

Female sex had a statistically significant positive effects on depression [adjusted β= 0.06, 95% CI:0.003, 0.109] , anxiety [adjusted β= 0.12, 95% CI: 0.059, 0.179], and somatic symptom [adjusted β=0.13, 95% CI: 0.074, 0.198] ; this implies that being females increase the level of level of depression, anxiety and somatic symptom as compared to males, controlling other factors constant. This finding is consistent with other studies [18, 37-39]. This could be explained by the fact that men and women have different brain chemistry and hormonal variations. Menstrual fluctuations in progesterone and estrogen may affect neurotransmitters in women, and low neurotransmitters may lead to depression anxiety and somatic symptom. It could also be that females are more likely to report on their mental illness. Cultures vary in terms of how males and females ought to deal with things such as depression and this might have an impact on how willing or unwilling males and females are report being depressed or having other mental health challenges [64, 65, 123, 124][125].

Alcohol use had a positive effect on depression [β= 0.14, 95% CI: 0.073, 0.201], anxiety [adjusted β= 0.11, 95% CI: 0.041, 0.188] and somatic symptom [adjusted β= 0.12, 95% CI: 0.062, 0.211]. This implied that an adolescent who had a history of alcohol drinking had a higher level of depression, anxiety and somatic symptom as compared to who did not drink. This finding is congruent with the study conducted in USA [45] and in China [46][129].This could be due to the fact that alcohol affects brain chemicals like serotonin and dopamine, which are responsible for happiness, and the decrease in thus chemicals leads to feeling down and depressed [130]. Furthermore, alcohol drinking may lead to depression, anxiety and somatic symptom by decreasing the age-appropriate activities of adolescents such as physical activity and social relationships. Besides, alcohol increases conflict with others [132] and conflict leads to stress that may lead to anxiety., depression and somatic symptom. Moreover it could be due to the direct effect of ethanol on nerve cell [131] which leads to heightened awareness of normal body sensation.

Students with lower self-rated academic ability had a higher levels of depression [adjusted β= -0.05, 95% CI= -0.083,-0.031] as compared with those with high self-rated academic ability. Similarly as self-rated academic ability of the student increases, the level of anxiety [adjusted β= -0.03, 95% CI: -0.065, -0.006] and somatic symptom [adjusted β= -0.02, 95%% CI: -0.039, -010] decrease. This result is compatible with the study conducted in Iran [134], in Slovakia [135] and with other studies[26, 59, 136]. The possible explanation for this might be, students with low academic ability are terrified of negative responses from teachers, parents, and friends, which may lead to fear about their future career, they will lose confidence in managing their own problems, which will lead them to become anxious, and anxiety will lead to depression. Moreover, it could be explained by encouragement and positive response from parents, teachers, and peers.]. This finding was supported by different studies.

Stress had a significant positive effect on depression [adjusted β= 0.76, 95% CI: 0.642, 0.900], anxiety [adjusted β= 1.10, 95% CI: 0.955, 1.264] , and somatic symptom [adjusted β= 086, 95% C: 0.700, 1.025]. This indicates that participants with high-level stress had higher levels of depression, anxiety and somatic symptom, holding other predictors constant. Our finding is supported with different literatures[49, 50][70]. The possible justification for this could be owing to the fact that high-level stress impairs the brain function, which may lead to fluctuations in neurotransmitters such as dopamine, which leads to depression. In addition, immune dysregulation during stress-full life events leads to anxiety and anxiety leads to depression and depression may leads to somatic symptoms.

Perceived social support had a significant direct negative effect [adjusted β= -0.13, 95% CI: -0.229,-0.029] on depression. This means that as social support increases, the level of depression decreased or students with low social support had higher levels of depression as compared to those who had high social support. This finding is compatible with different reports [43, 44, 52, 56]. The probable reason for this might be low social support which may increase feelings of loneliness, worthlessness, and hopelessness; leading to loss of interest in activities and depression. Moreover, students with low social support are more likely to have low self-esteem and negative cognition than those with higher social support, which may lead to depression [128].

Holding other predictors constant, medically confirmed chronic illness increased the level of depression [adjusted β= 0.21, 95% CI: 0.114, 0.311], anxiety [adjusted β= 0.25, 95% CI: 0.132, 0.371] and somatic symptom [adjusted β= 0.37, 95% CI: 0.239, 0.505]. This could be justified by the fact that chronic illness decreases the ability to do things, and people with chronic illness may fear the consequences of their illness. Which can lead to anxiety and anxiety can lead to depression and depression might lead to somatic symptom. Furthermore, it could be justified by the physical effect of the condition, the treatment effect for those who started treatment, which may directly lead to depression, and depression leads to anxiety. Direct effect of chronic illness on cognitive function may also be the possible reason.

Holding other factors constant, perceived family academic pressure had statistically significant positive effects [adjusted β= 0.04, 95% CI: 0.016, 0.060] on depression. Implies that participants with higher family academic pressure were related to higher level of depression. This finding is supported by a study conducted in Nepal [41] and in Vietnamese [42]. This could be the higher expectation of parents with higher academic pressure leading the student to stress which in turn leads to anxiety and anxiety leads to depression. Furthermore, students who fail to meet expectations may face harsh criticism, which may lead to self-doubt about their abilities, which may lead to a sense of worthlessness and hopelessness.

Learning in a private school had statistically significant positive effects [adjusted β= 0.10, 95% CI: 0.016, 0.207] on depression. It can be inferred that participants in private schools had a higher levels of depression compared to their counter parts. In contrast to our finding, the study conducted in India [40] showed that public school students had a higher level of depression than private school students; this disparity may be explained by differences in sample size and method of analysis; our study had a larger sample size than the India study, and the India study did not address the confounding effect. Higher levels of depression in private schools may be due to parents' expectations of high academic achievement in private schools; which lead students to be stressed when they try to meet their parents' expectations, and stress leads to depression.

Our findings showed that, holding other predictors constants, having a history of physical trauma had a direct positive effect [adjusted β= 0.06, 95% CI: 0.014, 0.125] on anxiety. This indicates that students with physical trauma had higher levels of anxiety as compared with those who had not. Our finding was a supported by the study conducted in Netherland [140]. This consistency could be justified by the fact that in reaction to trauma, neurotransmitters such as dopamine, serotonin, and gamma amino butyric acid may be impacted by cortisol, and depletion of thus neurotransmitters may lead to anxiety. Besides, individuals with physical trauma may internalize the event and may have subsequent negative thoughts about themselves; which affect their emotional experience, which might leads to anxiety [141].

A study conducted in India [142] reported that school type had not a significant relationship with anxiety. Contrarily, in our study school type had a significant relationship with level of anxiety [adjusted β= 0.28, 95% CI: 0.159, 0.415. It implied that adolescents in private schools experienced higher levels of anxiety than their peers did. It could be due to high control and supervision by parents and teachers in private schools. Furthermore, it might be due to the high academic expectation of students in private schools to satisfy their parents, which may lead to become anxious by fearing future fear of low academic achievement.

.

# Strength and limitations

Bidirectional relationship between anxiety and depression were assessed. Besides indirect and direct effect of different independent variables on different dependent variable were assessed simultaneously. However, this study is not without limitation. First only school adolescents in North West Ethiopia were included in the study which may affect the generalizability to all adolescents in Ethiopia. Second, self-reported screening tools were used, which may overestimate the effect size. Third, we just inquired about alcohol consumption once, not how much, how often, or for how many years it was consumed which may affect the result. Lastly, temporal relationship between predictors and outcome cannot be evaluated due to cross-sectional nature of the data.

Conclusions

In this study, moderate magnitudes of anxiety, depression, and somatic symptoms were found. Stress, school type, and sex had significant direct and indirect relationship with anxiety. Depression, self-rated academic ability and physical trauma had direct effects, while ever use of alcohol, medically confirmed chronic illness, perceived social support, perceived family academic pressure, and death of a loved one within the past 6 months had a significant indirect relationship with anxiety.

School type and anxiety were significantly related with depression both directly and indirectly. Perceived social support and having a history of death of a loved one within the past six months had statistically significant direct effect on depression. Whereas, having medically confirmed chronic illness, ever use of alcohol, perceived family academic pressure, sex, stress, self-rated academic ability, and having a history of physical trauma had a significant indirect relationship with depression.

Depression, stress, medically confirmed chronic illness, and being female were significantly related with somatic symptoms both directly and indirectly. Simultaneously, alcohol use in the past 6 months, school type, anxiety, self-rated academic ability, perceived social support, physical trauma, and death of a loved one had a significant indirect effect on somatic symptoms.

# Recommendations

Based on the study findings, authors suggest the following points: policy makers should strengthened the prevention and management of adolescence mental health disorders, it is better to initiate and implement school-based mental health screening services particularly for female students and students with low academic ability by educational office and schools. It is important to emphasize the two-way relationship between anxiety and depression and anxiety should be suspected in adolescents experiencing somatic symptoms. It is better to discourage adolescents alcohol drinking. Students should seek medical help if symptom of anxiety, and/or depression and/or somatic symptom happen and it is preferable to cultivate close relationships with parents and friends. Studies including out of school adolescent are recommended and it is better to assess temporal relationship by using follow up studies.

Abbreviations

AIC: Akaike information Criteria, OVB: Omitted variable bias, CFI: Comparative Fix index, CMB: Common Method Bias, OVB:Omitted Variable Bias, PHQ-9A: Patent Health Questionnaire for Adolescent, RMSEA: Rooted Mean Square Error of Approximation, TLI: Tucker Lewis Index,

# Acknowledgments

# The authors are grateful to University of Gondar, data collectors, supervisors, and study participants.

# Authors’ contribution

Conceptualization: Zenebe Abebe Gebreegziabher

Data curation: Zenebe Abebe Gebreegziabher. Ayenew Molla, Rediet Eristu

\Formal analysis:Zenebe Abebe Gebreegziabher

Investigation: Zenebe Abebe Gebreegziabher. Ayenew Molla, Rediet Eristu

Methodology: Zenebe Abebe Gebreegziabher. Ayenew Molla, Rediet Eristu

Project administration:Zenebe Abebe Gebreegziabher. Ayenew Molla, Rediet Eristu

Resource:Zenebe Abebe Gebreegziabher. Ayenew Molla, Rediet Eristu

Software: Zenebe Abebe Gebreegziabher, Ayenew Molla, Rediet Eristu

Supervision:Zenebe Abebe Gebreegziabher, Ayenew Molla, Rediet Eristu

Validation: Zenebe Abebe Gebreegziabher, Ayenew Molla, Rediet Eristu

Visualization:Zenebe Abebe Gebreegziabher

Writing – original draft: Zenebe Abebe Gebreegziabher

Writing – review and editing: Zenebe Abebe Gebreegziabher. Ayenew Molla, Rediet Eristu

# Supporting Information’s

S1 Table1: summary of key variables in the study

S1 Table2: intraclass correlation coefficients for each outcome variables

S1 Table3: Instrumental variable validity and strength

S1 Table4: depression among adolescents in Gondar town, 2022

S1 Table1 5: anxiety among adolescents in Gondar town, 2022

S1 Table6: somatic symptom among adolescent in Gondar town, 2022

S1 Table 7: Factorial average item parceling for depression, somatic symptom and their determinants among adolescents in Gondar town, 2022

S2 Fig 1: hypothetical measurement modelfor depression, somatic symptom and their determinants among adolescents in Gondar town, 2022

S3 annex 1: result of pilot study

S4 Table: tools to assess anxiety, depression, somatic symptom and their determinants among adolescents in Gondar town, 2022

S5 Data set: Data set of adolescents in Gondar town, 2022

# References

1.	American Psychiatric Association, D. and A.P. Association, *Diagnostic and statistical manual of mental disorders: DSM-5*. Vol. 5. 2013: American psychiatric association Washington, DC.

2.	World Health Organization, *The global burden of disease: 2004 update*. 2008: World Health Organization.

3.	Jörns-Presentati, A., et al., *The prevalence of mental health problems in sub-Saharan adolescents: A systematic review.* Plos one, 2021. **16**(5): p. e0251689.

4.	Armocida, B., et al., *Burden of non-communicable diseases among adolescents aged 10–24 years in the EU, 1990–2019: a systematic analysis of the Global Burden of Diseases Study 2019.* The Lancet Child & Adolescent Health, 2022.

5.	WHO, *Adolescents mental health.* 2019.

6.	Ryan, G., O. Qureshi, and N. Salaria, *Mental health and the 2030 Sustainable Development Agenda: global inaction on mental health is putting the brakes on development.* London, UK: London School of Hygiene and Tropical Medicine, 2018.

7.	Kassebaum, N.J., et al., *Global, regional, and national disability-adjusted life-years (DALYs) for 315 diseases and injuries and healthy life expectancy (HALE), 1990–2015: a systematic analysis for the Global Burden of Disease Study 2015.* The Lancet, 2016. **388**(10053): p. 1603-1658.

8.	Sayers, J., *The world health report 2001-Mental health: new understanding, new hope.* Bulletin of the World Health Organization, 2001. **79**: p. 1085-1085.

9.	Organization, W.H., *Depression and other common mental disorders: global health estimates*. 2017, World Health Organization.

10.	Perez, D.L., et al., *A neural circuit framework for somatosensory amplification in somatoform disorders.* J Neuropsychiatry Clin Neurosci, 2015. **27**(1): p. e40-50.

11.	WHO. *depression definition* 2019; Available from: <https://www.who.int/news-room/fact-sheets/detail/adolescent-mental-health>.

12.	Lancet, T., *Better understanding of youth mental health*. 2017. p. 1670.

13.	WHO., *Adolescents: health risks and solutions. Geneva: World Health Organization.* 2018.

14.	Tang, X., et al., *Prevalence of depressive symptoms among adolescents in secondary school in mainland China: A systematic review and meta-analysis.* J Affect Disord, 2019. **245**: p. 498-507.

15.	El-Missiry, A., et al., *Screening for depression in a sample of Egyptian secondary school female students.* J Affect Disord, 2012. **136**(1-2): p. e61-e68.

16.	Erskine, H.E., et al., *The global coverage of prevalence data for mental disorders in children and adolescents.* Epidemiol Psychiatr Sci, 2017. **26**(4): p. 395-402.

17.	Osborn, T.L., et al., *Depression and Anxiety Symptoms, Social Support, and Demographic Factors Among Kenyan High School Students.* Journal of Child and Family Studies, 2020. **29**(5): p. 1432-1443.

18.	Girma, S., et al., *Depression and its determinants among adolescents in Jimma town, Southwest Ethiopia.* Plos one, 2021. **16**(5): p. e0250927.

19.	Tirfeneh, E. and M. Srahbzu, *Depression and its association with parental neglect among adolescents at governmental high schools of Aksum town, Tigray, Ethiopia, 2019: a cross sectional study.* Depression research and treatment, 2020. **2020**.

20.	Bandelow, B. and S. Michaelis, *Epidemiology of anxiety disorders in the 21st century.* Dialogues in clinical neuroscience, 2022.

21.	Beesdo, K., et al., *Incidence and risk patterns of anxiety and depressive disorders and categorization of generalized anxiety disorder.* Archives of general psychiatry, 2010. **67**(1): p. 47-57.

22.	Yang, X., et al., *Global, regional and national burden of anxiety disorders from 1990 to 2019: results from the Global Burden of Disease Study 2019.* Epidemiology and Psychiatric Sciences, 2021. **30**: p. e36.

23.	Vos, T., et al., *Global burden of 369 diseases and injuries in 204 countries and territories, 1990–2019: a systematic analysis for the Global Burden of Disease Study 2019.* The Lancet, 2020. **396**(10258): p. 1204-1222.

24.	Kessler, R.C., et al., *Twelve‐month and lifetime prevalence and lifetime morbid risk of anxiety and mood disorders in the United States.* International journal of methods in psychiatric research, 2012. **21**(3): p. 169-184.

25.	Biswas, T., et al., *Global variation in the prevalence of suicidal ideation, anxiety and their correlates among adolescents: a population based study of 82 countries.* EClinicalMedicine, 2020. **24**: p. 100395.

26.	Malak, M.Z. and A.H. Khalifeh, *Anxiety and depression among school students in Jordan: Prevalence, risk factors, and predictors.* Perspectives in psychiatric care, 2018. **54**(2): p. 242-250.

27.	Sandal, R.K., et al., *Prevalence of Depression, Anxiety and Stress among school going adolescent in Chandigarh.* Journal of family medicine and primary care, 2017. **6**(2): p. 405-410.

28.	Bifftu, B. and Y. Guracho, *A Systematic Review and Meta-Analysis of Anxiety among Children and Youth in Ethiopia.* J Depress Anxiety Disord, 2021. **3**(2): p. 89-100.

29.	Eriksen, T.E. and M.B. Risør, *What is called symptom?* Med Health Care Philos, 2014. **17**(1): p. 89-102.

30.	Edgcomb, J.B., C.H. Tseng, and B. Kerner, *Medically unexplained somatic symptoms and bipolar spectrum disorders: A systematic review and meta-analysis.* J Affect Disord, 2016. **204**: p. 205-13.

31.	Tomenson, B., et al., *Total somatic symptom score as a predictor of health outcome in somatic symptom disorders.* Br J Psychiatry, 2013. **203**(5): p. 373-80.

32.	Löwe, B., et al., *Depression, anxiety and somatization in primary care: syndrome overlap and functional impairment.* General hospital psychiatry, 2008. **30**(3): p. 191-199.

33.	Kroenke, K. and A.D. Mangelsdorff, *Common symptoms in ambulatory care: Incidence, evaluation, therapy, and outcome.* The American Journal of Medicine, 1989. **86**(3): p. 262-266.

34.	Kurlansik, S.L. and M.S. Maffei, *Somatic symptom disorder.* American family physician, 2016. **93**(1): p. 49-54.

35.	Martin, J.C.S., et al., *Somatic complaints and symptoms of anxiety and depression in a school-based sample of preadolescents and early adolescents. Functional impairment and implications for treatment.* Journal of Cognitive and Behavioral Psychotherapies, 2011. **11**(2): p. 191-208.

36.	Carlsson, J., *Prevalence of headache in schoolchildren: relation to family and school factors.* Acta Paediatr, 1996. **85**(6): p. 692-6.

37.	Trivedi, D., et al., *Depression among adolescent students in South India: How serious is the issue?* Indian Journal of Psychiatry, 2016. **58**(3): p. 349-350.

38.	Raheel, H., *Depression and associated factors among adolescent females in Riyadh, Kingdom of Saudi Arabia, a cross-sectional study.* International journal of preventive medicine, 2015. **6**.

39.	Rehm, J. and K.D. Shield, *Global burden of disease and the impact of mental and addictive disorders.* Current psychiatry reports, 2019. **21**(2): p. 1-7.

40.	Kumar, R.K., et al., *The prevalence of depression, anxiety, and stress among high school adolescent's children in public and private schools in Rangareddy district Telangana state: A cross-sectional study.* Journal of Education and Health Promotion, 2022. **11**(1): p. 83.

41.	BP, C., *Stress, anxiety, and depression among adolescent students of public schools in Kathmandu.* Journal of Institute of Medicine, 2018. **40**(3).

42.	Nguyen, D.T., et al., *Depression, anxiety, and suicidal ideation among Vietnamese secondary school students and proposed solutions: a cross-sectional study.* BMC public health, 2013. **13**(1): p. 1-10.

43.	Addy, N.D., et al., *Mental health difficulties, coping mechanisms and support systems among school-going adolescents in Ghana: A mixed-methods study.* PLoS one, 2021. **16**(4): p. e0250424.

44.	Deb, S., E. Strodl, and H. Sun, *Academic stress, parental pressure, anxiety and mental health among Indian high school students.* International Journal of Psychology and Behavioral Science, 2015. **5**(1): p. 26-34.

45.	Galaif, E.R., et al., *Suicidality, depression, and alcohol use among adolescents: a review of empirical findings.* International journal of adolescent medicine and health, 2007. **19**(1): p. 27-36.

46.	Huang, R., et al., *Reported alcohol drinking and mental health problems in Hong Kong Chinese adolescents.* Drug and alcohol dependence, 2016. **164**: p. 47-54.

47.	Shah, S.M., et al., *Self-esteem and other risk factors for depressive symptoms among adolescents in United Arab Emirates.* PloS one, 2020. **15**(1): p. e0227483.

48.	Gülsen, A. and B. Uygur, *Psychological Features of Smokers.* Respir Care, 2018. **63**(12): p. 1492-1497.

49.	Ibrahim, N., et al., *Prevalence and predictors of depression and suicidal ideation among adolescents attending government secondary schools in Malaysia.* Medical Journal of Malaysia, 2017. **72**(4): p. 221-227.

50.	Ghobadzadeh, M., et al., *Relationships between adolescent stress, depressive symptoms, and sexual risk behavior in young adulthood: A structural equation modeling analysis.* Journal of Pediatric Health Care, 2019. **33**(4): p. 394-403.

51.	Lindholdt, L., et al., *Perceived stress among adolescents as a marker for future mental disorders: A prospective cohort study.* Scandinavian Journal of Public Health, 2021: p. 1403494821993719.

52.	Cao, C., et al., *Anxiety, depression, and PTSD symptoms among high school students in china in response to the COVID-19 pandemic and lockdown.* Journal of Affective Disorders, 2022. **296**: p. 126-129.

53.	AlAzzam, M., et al., *Predictors of depression and anxiety among senior high school students during COVID-19 pandemic: The context of home quarantine and online education.* The Journal of School Nursing, 2021. **37**(4): p. 241-248.

54.	Wittchen, H.-U., C.A. Essau, and J.-C. Krieg, *Anxiety disorders: similarities and differences of comorbidity in treated and untreated groups.* The British Journal of Psychiatry, 1991. **159**(S12): p. 23-33.

55.	Essau, C.A., J. Conradt, and F. Petermann, *Frequency, comorbidity, and psychosocial impairment of anxiety disorders in German adolescents.* Journal of anxiety disorders, 2000. **14**(3): p. 263-279.

56.	Chang, C.-W., R. Yuan, and J.-K. Chen, *Social support and depression among Chinese adolescents: The mediating roles of self-esteem and self-efficacy.* Children and Youth Services Review, 2018. **88**: p. 128-134.

57.	Tramonte, L. and D. Willms, *The prevalence of anxiety among middle and secondary school students in Canada.* Canadian Journal of Public Health/Revue Canadienne de Sante'e Publique, 2010: p. S19-S22.

58.	Smith, L., et al., *Correlates of symptoms of anxiety and depression and mental wellbeing associated with COVID-19: a cross-sectional study of UK-based respondents.* Psychiatry Res, 2020. **291**: p. 113138.

59.	Syokwaa, K.A., P.J. Aloka, and S.N.F. Ndunge, *The relationship between anxiety levels and academic achievement among students in selected secondary schools in Lang’ata District, Kenya.* Journal of Educational and Social Research, 2014. **4**(3): p. 403-403.

60.	Khanum, F. and S. Razack, *Anxiety–Herbal treatment: A review.* Res Rev Biomed Biotech, 2010. **1**(2): p. 83-89.

61.	Kumari, A. and J. Jain, *Examination stress and anxiety: A study of college students.* Global Journal of Multidisciplinary Studies, 2014. **4**(1): p. 31-40.

62.	Bekhuis, E., et al., *Differential associations of specific depressive and anxiety disorders with somatic symptoms.* Journal of psychosomatic research, 2015. **78**(2): p. 116-122.

63.	Rice, F., M.B.M. van den Bree, and A. Thapar, *A population-based study of anxiety as a precursor for depression in childhood and adolescence.* BMC Psychiatry, 2004. **4**(1): p. 43.

64.	Karaca, S., et al., *Somatic Symptoms in Secondary School Students and Parental Attitudes.* Journal of Psychiatric Nursing/Psikiyatri Hemsireleri Dernegi, 2015. **6**(3).

65.	Haraldstad, K. and T.H. Stea, *Associations between pain, self-efficacy, sleep duration, and symptoms of depression in adolescents: a cross-sectional survey.* BMC public health, 2021. **21**(1): p. 1-7.

66.	Larsson, B.S., *Somatic complaints and their relationship to depressive symptoms in Swedish adolescents.* Journal of Child Psychology and Psychiatry, 1991. **32**(5): p. 821-832.

67.	Heidari, Z., et al., *Demographic and Life Styles Determinants of Somatic Complaints' Structures: A Cross-sectional Study on a Large Sample of Iranian Adults Using Factor Mixture Model.* Int J Prev Med, 2017. **8**: p. 8.

68.	Ruotsalainen, H., et al., *Systematic review of physical activity and exercise interventions on body mass indices, subsequent physical activity and psychological symptoms in overweight and obese adolescents.* Journal of advanced nursing, 2015. **71**(11): p. 2461-2477.

69.	Noh, J.-W., et al., *Relationships between Extra-School Tutoring Time, Somatic Symptoms, and Sleep Duration of Adolescent Students: A Panel Analysis Using Data from the Korean Children and Youth Panel Survey.* International Journal of Environmental Research and Public Health, 2020. **17**(21): p. 8037.

70.	Glise, K., G. Ahlborg, and I.H. Jonsdottir, *Prevalence and course of somatic symptoms in patients with stress-related exhaustion: does sex or age matter.* BMC psychiatry, 2014. **14**(1): p. 1-13.

71.	Garralda, M.E., *Unexplained physical complaints.* Pediatric Clinics, 2011. **58**(4): p. 803-813.

72.	Glise, K., G. Ahlborg, Jr., and I.H. Jonsdottir, *Prevalence and course of somatic symptoms in patients with stress-related exhaustion: does sex or age matter.* BMC psychiatry, 2014. **14**: p. 118-118.

73.	van Boven, K., et al., *Do unexplained symptoms predict anxiety or depression? Ten-year data from a practice-based research network.* British Journal of General Practice, 2011. **61**(587): p. e316-e325.

74.	Kingery, J.N., G.S. Ginsburg, and C.A. Alfano, *Somatic symptoms and anxiety among African American adolescents.* Journal of Black Psychology, 2007. **33**(4): p. 363-378.

75.	Janssens, K.A., et al., *Anxiety and depression are risk factors rather than consequences of functional somatic symptoms in a general population of adolescents: the TRAILS study.* Journal of Child Psychology and Psychiatry, 2010. **51**(3): p. 304-312.

76.	Knook, L.M., et al., *Psychiatric disorders in children and adolescents presenting with unexplained chronic pain: what is the prevalence and clinical relevancy?* European child & adolescent psychiatry, 2011. **20**(1): p. 39-48.

77.	Rojas, G., et al., *[Association between drug consumption and depression among high school students].* Rev Med Chil, 2012. **140**(2): p. 184-91.

78.	Yen, C.F., et al., *Tattooing among high school students in southern Taiwan: the prevalence, correlates and associations with risk-taking behaviors and depression.* Kaohsiung J Med Sci, 2012. **28**(7): p. 383-9.

79.	Mackenzie, S., et al., *Depression and suicide ideation among students accessing campus health care.* Am J Orthopsychiatry, 2011. **81**(1): p. 101-7.

80.	Dunlavy, A.C., E.O. Aquah, and M.L. Wilson, *Suicidal ideation among school-attending adolescents in Dar es Salaam, Tanzania.* Tanzania Journal of Health Research, 2015. **17**(1).

81.	World Health Organization, 2021.

82.	Clayborne, Z.M., M. Varin, and I. Colman, *Systematic review and meta-analysis: adolescent depression and long-term psychosocial outcomes.* Journal of the American Academy of Child & Adolescent Psychiatry, 2019. **58**(1): p. 72-79.

83.	Saxena, S. and Y. Setoya, *World Health Organization's Comprehensive Mental Health Action Plan 2013–2020.* 2014.

84.	Murray, A.M., et al., *Barriers to the diagnosis of somatoform disorders in primary care: protocol for a systematic review of the current status.* Systematic reviews, 2013. **2**(1): p. 1-8.

85.	Abdulghani, H.M., et al., *Stress and its effects on medical students: a cross-sectional study at a college of medicine in Saudi Arabia.* Journal of health, population, and nutrition, 2011. **29**(5): p. 516.

86.	Organization, W.H., *World health statistics overview 2019: monitoring health for the SDGs, sustainable development goals*. 2019, World Health Organization.

87.	Erskine, H., et al., *The global coverage of prevalence data for mental disorders in children and adolescents.* Epidemiology and psychiatric sciences, 2017. **26**(4): p. 395-402.

88.	report, G.c.a., *total population estimation report.* Gondar city adminstrative office, 2022.

89.	Organization, W.H., *Mental health status of adolescents in South-East Asia: Evidence for action.* 2017.

90.	Bentler, P.M. and C.-P. Chou, *Practical issues in structural modeling.* Sociological methods & research, 1987. **16**(1): p. 78-117.

91.	Kroenke, K., R.L. Spitzer, and J.B. Williams, *The PHQ-9: validity of a brief depression severity measure.* J Gen Intern Med, 2001. **16**(9): p. 606-13.

92.	Spitzer, R.L., et al., *A brief measure for assessing generalized anxiety disorder: the GAD-7.* Archives of internal medicine, 2006. **166**(10): p. 1092-1097.

93.	Spitzer, R.L., et al., *A Brief Measure for Assessing Generalized Anxiety Disorder: The GAD-7.* Archives of Internal Medicine, 2006. **166**(10): p. 1092-1097.

94.	Toussaint, A., et al., *Detecting DSM-5 somatic symptom disorder: criterion validity of the Patient Health Questionnaire-15 (PHQ-15) and the Somatic Symptom Scale-8 (SSS-8) in combination with the Somatic Symptom Disorder – B Criteria Scale (SSD-12).* Psychological Medicine, 2019. **50**(2): p. 324-333.

95.	Gierk, B., et al., *The somatic symptom scale–8 (SSS-8): a brief measure of somatic symptom burden.* JAMA internal medicine, 2014. **174**(3): p. 399-407.

96.	Cohen, S., T. Kamarck, and R. Mermelstein, *A global measure of perceived stress.* Journal of health and social behavior, 1983: p. 385-396.

97.	Bøen, H., O.S. Dalgard, and E. Bjertness, *The importance of social support in the associations between psychological distress and somatic health problems and socio-economic factors among older adults living at home: a cross sectional study.* BMC geriatrics, 2012. **12**(1): p. 1-12.

98.	Prochaska, J.J., J.F. Sallis, and B. Long, *A physical activity screening measure for use with adolescents in primary care.* Archives of pediatrics & adolescent medicine, 2001. **155**(5): p. 554-559.

99.	Group, W.A.W., *The alcohol, smoking and substance involvement screening test (ASSIST): development, reliability and feasibility.* Addiction, 2002. **97**(9): p. 1183-1194.

100.	Kroenke, K., R.L. Spitzer, and J.B. Williams, *The PHQ‐9: validity of a brief depression severity measure.* Journal of general internal medicine, 2001. **16**(9): p. 606-613.

101.	Gierk, B., et al., *Assessing somatic symptom burden: A psychometric comparison of the Patient Health Questionnaire—15 (PHQ-15) and the Somatic Symptom Scale—8 (SSS-8).* Journal of Psychosomatic Research, 2015. **78**(4): p. 352-355.

102.	Clark, L.A. and D. Watson, *Constructing validity: Basic issues in objective scale development.* 2016.

103.	Fornell, C. and D.F. Larcker, *Structural equation models with unobservable variables and measurement error: Algebra and statistics*. 1981, Sage Publications Sage CA: Los Angeles, CA.

104.	Shukur, G. and P. Mantalos, *Size and power of the RESET test as applied to systems of equations: a bootstrap approach.* Journal of modern applied statistical methods, 2004. **3**(2): p. 10.

105.	Kline, R.B., *Principles and practice of structural equation modeling*. 2015: Guilford publications.

106.	Kumar, S. and S. Kumar, *Structure equation modeling basic assumptions and concepts: A novices guide.* Asian Journal of Management Sciences, 2015. **3**(07): p. 25-28.

107.	Roth, P.L. and F.S. Switzer III, *A Monte Carlo analysis of missing data techniques in a HRM setting.* Journal of Management, 1995. **21**(5): p. 1003-1023.

108.	Aguirre-Urreta, M.I. and J. Hu, *Detecting common method bias: Performance of the Harman's single-factor test.* ACM SIGMIS Database: the DATABASE for Advances in Information Systems, 2019. **50**(2): p. 45-70.

109.	Paxton, P., et al., *Nonrecursive models: Endogeneity, reciprocal relationships, and feedback loops*. Vol. 168. 2011: Sage Publications.

110.	Smith, T.D. and B.F. McMillan, *A Primer of Model Fit Indices in Structural Equation Modeling.* 2001.

111.	Hoyle, R.H., *The structural equation modeling approach: Basic concepts and fundamental issues.* 1995.

112.	Technology, E.F.M.o.S.a., *national-research-ethics-review-guidline.* 2014.

113.	Steiger, J.H., *Structural model evaluation and modification: An interval estimation approach.* Multivariate behavioral research, 1990. **25**(2): p. 173-180.

114.	Bhattarai, D., N. Shrestha, and S. Paudel, *Prevalence and factors associated with depression among higher secondary school adolescents of Pokhara Metropolitan, Nepal: a cross-sectional study.* BMJ Open, 2020. **10**(12): p. e044042.

115.	Anjum, A., et al., *Investigating the prevalence of and factors associated with depressive symptoms among urban and semi-urban school adolescents in Bangladesh: a pilot study.* International health, 2019.

116.	Urmila, K., et al., *Prevalence and risk factors associated with depression among higher secondary school students residing in a boarding school of North Kerala, India.* Int J Contemp Pediatr, 2017. **4**(3): p. 735.

117.	Satcher, D., *Mental health: Culture, race, and ethnicity—A supplement to mental health: A report of the surgeon general*. 2001: US Department of Health and Human Services.

118.	Khalid S, A.G., A.A. Hasan S, and M. Ossama A, *Prevalence of depression, anxiety and stress as measured by the depression, anxiety, and stress scale [DASS-42] among secondary school girls in Abha, Saudi Arabia.* 2009.

119.	Alharbi, R., et al., *Depression and anxiety among high school student at Qassim Region.* Journal of family medicine and primary care, 2019. **8**(2): p. 504.

120.	Polanczyk, G.V., et al., *Annual research review: A meta‐analysis of the worldwide prevalence of mental disorders in children and adolescents.* Journal of child psychology and psychiatry, 2015. **56**(3): p. 345-365.

121.	Widiger, T.A., P.T. Costa, and A.P. Association, *Personality disorders and the five-factor model of personality*. 2013: JSTOR.

122.	Bener, A., S. Ghuloum, and F.T. Burgut, *Gender differences in prevalence of somatoform disorders in patients visiting primary care centers.* Journal of primary care & community health, 2010. **1**(1): p. 37-42.

123.	Johnson, D.P. and M.A. Whisman, *Gender differences in rumination: A meta-analysis.* Personality and individual differences, 2013. **55**(4): p. 367-374.

124.	Wharton, W., et al., *Neurobiological underpinnings of the estrogen-mood relationship.* Current psychiatry reviews, 2012. **8**(3): p. 247-256.

125.	Narbone, M., et al., *Hormonal disregulation and catamenial epilepsy.* Functional Neurology, 1990. **5**(1): p. 49-53.

126.	Moksnes, U.K. and G.A. Espnes, *Self‐esteem and emotional health in adolescents–gender and age as potential moderators.* Scandinavian Journal of Psychology, 2012. **53**(6): p. 483-489.

127.	Altemus, M., N. Sarvaiya, and C.N. Epperson, *Sex differences in anxiety and depression clinical perspectives.* Frontiers in neuroendocrinology, 2014. **35**(3): p. 320-330.

128.	Li, Y., et al., *Social support, attachment closeness, and self-esteem affect depression in international students in China.* Frontiers in Psychology, 2021. **12**: p. 618105.

129.	Huang, R., et al., *Reported alcohol drinking and mental health problems in Hong Kong Chinese adolescents.* Drug and Alcohol Dependence, 2016. **164**: p. 47-54.

130.	Deykin, E.Y., J.C. Levy, and V. Wells, *Adolescent depression, alcohol and drug abuse.* American Journal of Public Health, 1987. **77**(2): p. 178-182.

131.	Guerri, C. and M. Pascual, *Mechanisms involved in the neurotoxic, cognitive, and neurobehavioral effects of alcohol consumption during adolescence.* Alcohol, 2010. **44**(1): p. 15-26.

132.	Miller, J.W., et al., *Binge drinking and associated health risk behaviors among high school students.* Pediatrics, 2007. **119**(1): p. 76-85.

133.	Hassan, I. and R. Ali, *The association between somatic symptoms, anxiety disorders and substance use. A literature review.* Psychiatric quarterly, 2011. **82**(4): p. 315-328.

134.	Yousefi, F., et al., *The relationship between gender, age, depression and academic achievement.* 2010.

135.	Demuthova, S., *The School Environment as a Source of Somatic Problems in Adolescents.* Revista Românească pentru Educaţie Multidimensională, 2019. **11**(2): p. 59-71.

136.	El-Anzi, F.O., *Academic achievement and its relationship with anxiety, self-esteem, optimism, and pessimism in Kuwaiti students.* Social Behavior and Personality: an international journal, 2005. **33**(1): p. 95-104.

137.	Care, B., *Children and grief. A reference list compiled by dr. philos. Atle Dyregrov Center for Crisis Psychology, Bergen, Norway.* School Psychology International, 1994. **22**: p. 417-433.

138.	Kendler, K.S., et al., *The structure of genetic and environmental risk factors for common psychiatric and substance use disorders in men and women.* Archives of general psychiatry, 2003. **60**(9): p. 929-937.

139.	Da Costa, L.C. and B. Mash, *A description of the psychosocial factors associated with depression and anxiety in South African adolescents attending urban private practices in Johannesburg.* South African Family Practice, 2008. **50**(5): p. 51-51.

140.	Hovens, J.G., et al., *Childhood life events and childhood trauma in adult patients with depressive, anxiety and comorbid disorders vs. controls.* Acta psychiatrica scandinavica, 2010. **122**(1): p. 66-74.

141.	Association, A.P., *DSM 5 diagnostic and statistical manual of mental disorders*, in *DSM 5 Diagnostic and statistical manual of mental disorders*. 2013. p. 947 p.-947 p.

142.	Kumar, R.K., et al., *The prevalence of depression, anxiety, and stress among high school adolescent's children in public and private schools in Rangareddy district Telangana state: A cross-sectional study.* Journal of Education and Health Promotion, 2022. **11**.

143.	Cottrell, D.J., *Fifteen-minute consultation: Medically unexplained symptoms.* Archives of Disease in Childhood-Education and Practice, 2016. **101**(3): p. 114-118.

144.	Petkus, A.J., et al., *Trauma history is associated with psychological distress and somatic symptoms in homebound older adults.* The American Journal of Geriatric Psychiatry, 2009. **17**(9): p. 810-818.
