# Supplementary material for: Determinants of adolescents’ depression, anxiety, and somatic symptoms in Northwest Ethiopia: A non-recursive structural equation modeling
Source: PLoS One. 2024 Apr 10;19(4):e0281571. doi: 10.1371/journal.pone.0281571 (PMC11006201; doi:10.1371/journal.pone.0281571)
Supplement: Attachment — Submitted filename: Response to Editors and Reviewers edited.docx [file pone.0281571.s016.docx]

**Rebuttal letter**

1. **Response to chief academic editor**

**Manuscript title:** Depression, Anxiety, somatic symptom and their determinants among high school students in North West Ethiopia: Non-recursive structural equation modeling

**Manuscript ID:** PONE-D-23-02244

**Authors’ response**: Dear Editors and Reviewers, Thank you for your constructive feedback regarding our manuscript. The authors are appreciative of the editors' and reviewers' helpful criticism, insight, time, and positive assessments of our work. Based on the chief editor's and reviewers' comments, we made numerous new and clarifying statements throughout the entire document. The revised text was marked and attached as a separate file. These changes have improved the manuscript considerably and we hope that it can be published without delay. All authors have read the revised version of the manuscript and do not have any conflict of interest. We appreciate the editors and reviewers for their time and critical comments to improve our manuscript and we addressed the comments point by point as follows. We hope the revised manuscript satisfies the chief editor as well respected reviewers. If there are any unresolved questions, please do not hesitate to contact us again.

**Editor:** Journal requirements:

When submitting your revision, we need you to address these additional requirements.

1. Please ensure that your manuscript meets PLOS ONE's style requirements, including those for file naming. The PLOS ONE style templates can be found at

<https://journals.plos.org/plosone/s/file?id=wjVg/PLOSOne_formatting_sample_main_body.pdf> and

<https://journals.plos.org/plosone/s/file?id=ba62/PLOSOne_formatting_sample_title_authors_affiliations.pdf>

Author response: We reviewed and corrected the formatting in accordance with the PLOS ONE criteria.

**Editor:** 2. In your Data Availability statement, you have not specified where the minimal data set underlying the results described in your manuscript can be found. PLOS defines a study's minimal data set as the underlying data used to reach the conclusions drawn in the manuscript and any additional data required to replicate the reported study findings in their entirety. All PLOS journals require that the minimal data set be made fully available. For more information about our data policy, please see <http://journals.plos.org/plosone/s/data-availability>.
Upon re-submitting your revised manuscript, please upload your study’s minimal underlying data set as either Supporting Information files or to a stable, public repository and include the relevant URLs, DOIs, or accession numbers within your revised cover letter. For a list of acceptable repositories, please see <http://journals.plos.org/plosone/s/data-availability#loc-recommended-repositories>. Any potentially identifying patient information must be fully anonymized.

Important: If there are ethical or legal restrictions to sharing your data publicly, please explain these restrictions in detail. Please see our guidelines for more information on what we consider unacceptable restrictions to publicly sharing data: <http://journals.plos.org/plosone/s/data-availability#loc-unacceptable-data-access-restrictions>. Note that it is not acceptable for the authors to be the sole named individuals responsible for ensuring data access.
We will update your Data Availability statement to reflect the information you provide in your cover letter.

Author response: Dear PLOSONE journal editors, thank you for your suggestion to include the data availability statement in the manuscript. We accepted your comment and included it in the supplementary file on the revised submission.

**Editor:** If applicable, we recommend that you deposit your laboratory protocols in [protocols.io](http://protocols.io/) to enhance the reproducibility of your results. Protocols.io assigns your protocol its own identifier (DOI) so that it can be cited independently in the future. For instructions see: <https://journals.plos.org/plosone/s/submission-guidelines#loc-laboratory-protocols>. Additionally, PLOS ONE offers an option for publishing peer-reviewed Lab Protocol articles, which describe protocols hosted on [protocols.io](http://protocols.io/). Read more information on sharing protocols at <https://plos.org/protocols?utm_medium=editorial-email&utm_source=authorletters&utm_campaign=protocols>.

**Authors’ response:** not applicable.

**Editor:** 3. Please review your reference list to ensure that it is complete and correct. If you have cited papers that have been retracted, please include the rationale for doing so in the manuscript text, or remove these references and replace them with relevant current references. Any changes to the reference list should be mentioned in the rebuttal letter that accompanies your revised manuscript. If you need to cite a retracted article, indicate the article’s retracted status in the References list and also include a citation and full reference for the retraction notice.

**Authors’ response:** We appreciate the editors' for their use full insight to check the presence of retracted papers in our reference list, we revised our references and there is no reference list from retracted paper in our manuscript.

Additional Editor Comments:

The study is well-designed. However, the expression style has to be modified for better clarity.
Please adjust the reviewers' comments. (See attachment from reviewer 2)

**Authors’ response**: Thank you for your advice on how to make our work better. We have incorporated these into the revised manuscript along with the second reviewers' ideas, and the language used throughout the manuscript, notably in the result and discussion sections has been amended accordingly.

**Title:**
**Editor:** Please change the title into “…Ethiopia**: A** Non-recursive structural…”

**Authors’ response:** Accepted and corrected (line number 2-3).

**Abstract:**
**Editor:** Please see the recommended change as attached.

**Authors’ response**: Thank you for the suggestion, we accepted it and modified accordingly (line number 12, 25, 33).

**Methodology and Discussion:**
**Editor:** I agree with the paper organization issue pointed out by reviewer 2. The current version is still satisfactory. Please see detailed comments as attached.

**Authors’ response:** We appreciate your feedback on how to make our paper better, and we have addressed all of your concerns in the amended version (example: line number 45-47, 151,217 and like).

**Discussion:**
**Editor:** Please condense the 3 paragraphs in “Magnitude of depression, anxiety and somatic symptoms” into a single paragraph.

**Authors’ response:** Accepted and modified (line number 441-452).

**Editor:** It is unnecessary for the author to repeat the same logic three times despite different outcomes (report finding, compare findings with multiple previous studies, explained the difference might be a result of screening tools). It is also unnecessary for the author to elaborate on details of previous studies. The reviewer 2 pointed out the result is too long. So is discussion. Please see the rest of comments as attached

**Authors’ response**: Accepted and modified (line number 454-462).

**General:**

**Editor:** Specific terms need to be better defined:
The author used the term “magnitude” and “prevalence” alternatively to describe the outcomes including depression, anxiety, and somatic symptoms. Please clarify the definition of these two terms and use the correct one. The author may find it beneficial to consult a statistician for better description.

**Authors’ response:** We appreciate the wise advice you provided. We consulted statistician regarding the terms magnitude and prevalence; in our instance, prevalence was the more acceptable term, thus we replaced magnitude with prevalence throughout the entire document (Example: line number 25, 33, 331, 337, and 343).

**Editor:** Participants:
Please define the participant’s age in both the text and tables. (Grades are not satisfactory in this case) If the author includes any participants aged under 12, the participant description should be revised into “children and adolescent” through the whole manuscript.

**Authors’ response:** We appreciate your wonderful view, and we've accepted and incorporated it in the revised version (line number 316 and table 3 page 18).

**Editor:** Logic of clinical application:
It is suggested that the author invite a clinical practitioner working with the investigated population to go through the study. The author highlights the correlation between depression-anxiety-somatic symptoms as the main outcome; alcohol use, sex and more are correlated with unfavorable mental health situation. However, these findings did not provide novelty to the field. The value of this study is the unique study population. The author could have better elaborate how the results could be explained by environmental factors specific in Northwest Ethiopia and how that could affect policy making/clinical practice.

**Authors’ response:** We acknowledged your advice, and in the amended paper, we attempted to reflect the particular destiny of adolescents in Northwest Ethiopia (line number 106-107 and 461-462).

**Editor:** Writing quality:
Please correct all the typos, lower case/upper case, repetitive content, inconsistent font, content generated during the reversing process which supposed to be deleted from the formal manuscript. These are the red flags for the editor that suggested lacking effort in proofreading

**Authors’ response:** Thank you for your intelligent idea; based on it, we proofread the paper several times and attempted to eradicate all mistakes, upper and lower case differences, and inconsistencies.

**Editor:** Language:
The syntax is confusing. Please consider having a senior author or peer to help with better expression.

**Authors’ response:** Thank you for your comments; we consulted senior authors and attempted to adjust the syntax based on their suggestions.

Reviewer's Responses to Questions

**Comments to the Author**

1. Is the manuscript technically sound, and do the data support the conclusions?
The manuscript must describe a technically sound piece of scientific research with data that supports the conclusions. Experiments must have been conducted rigorously, with appropriate controls, replication, and sample sizes. The conclusions must be drawn appropriately based on the data presented.

Reviewer #1: Yes

Reviewer #2: Yes

2. Has the statistical analysis been performed appropriately and rigorously?

Reviewer #1: Yes

Reviewer #2: Yes

3. Have the authors made all data underlying the findings in their manuscript fully available?
The [PLOS Data policy](http://www.plosone.org/static/policies.action#sharing) requires authors to make all data underlying the findings described in their manuscript fully available without restriction, with rare exception (please refer to the Data Availability Statement in the manuscript PDF file). The data should be provided as part of the manuscript or its supporting information, or deposited to a public repository. For example, in addition to summary statistics, the data points behind means, medians and variance measures should be available. If there are restrictions on publicly sharing data—e.g. participant privacy or use of data from a third party—those must be specified.

Reviewer #1: Yes

Reviewer #2: Yes

4. Is the manuscript presented in an intelligible fashion and written in standard English?
PLOS ONE does not copyedit accepted manuscripts, so the language in submitted articles must be clear, correct, and unambiguous. Any typographical or grammatical errors should be corrected at revision, so please note any specific errors here.

Reviewer #1: Yes

Reviewer #2: Yes

5. Review Comments to the Author\
Please use the space provided to explain your answers to the questions above. You may also include additional comments for the author, including concerns about dual publication, research ethics, or publication ethics. (Please upload your review as an attachment if it exceeds 20,000 characters)

2. **Response to Reviewer #1:**

Authors’ response: First of all, we would like to say thank you for your great effort, commitment, humility, and time to comment our manuscript. Next, we would like to respond to the comments point-by-point. All the points raised are corrected accordingly in the main manuscript and put in track change.

General Feedback!

Reviewer 1: The write up, grammar and integrity is good

Eligibilitycriteria
**Reviewer #1**: Was there no exclusion criteria used?

**Author response:** We appreciate the reviewer's suggestion, night preparatory and high schools students, as well as those who moved in from other areas after January 8, 2022, were excluded; this was already covered in the initial submission's lines 155, 156, and 157. We've also included it from line 137 to 139 in the updated version..
Operational Definition

**Reviewer #1**: Do you consider the person with PHQ-9A score of less than 9 have no depression? A person with a PHQ-9A score of 5-9 has mild depression. Do you think missing these segments of the population has effect on your result?

Author response: Thank you for the input. We used the cut-off point above 9, as originally specified in line 217 to 218 of the initial submission. We used the cut-off point above 9, as described in lines 217 to 21 of the first submission. We were also concerned about missing people with mild depression, but we made an effort to read many papers on the threshold for diagnosing depression in the general population. Even though the cut-off point used by clinicians and researchers varies depending on the clinical group, a PHQ-9 score of 10 or higher is more consistently and strongly recommended for community depression diagnosis (Gilbody, Richards et al. 2007), since it is consistent with DSM V criteria for depression diagnosis..

**Strength and Limitation of the study
Reviewer #1**: ‘Assed’: Good to write in full assessed on line 955

Author response: Thank you, we have accepted and modified it (Line number 543).

3. **Response to Reviewer #2**:

Authors’ response: First of all, we would like to say thank you for your great effort, commitment, humility, and time to comment on our manuscript. Next, we would like to respond to the comments point-by-point. All the points raised are corrected accordingly in the main manuscript and put in track change.

**Reviewer #2:** To the best of my knowledge the study was well executed, the data sound and the analyses appropriate (though I leave the final judgement on analysis to reviewers more expert than me in SEM). The manuscript is on an important topic, it is reasonably well-written and the study findings ought to be brought to light.

However, considerable re-organization and making the paper far more concise will be required to meet the standards for publication, in any journal.

**Author response**: Thank you for your insightful suggestion. We modified and corrected the document in the revised manuscript according to your general and specific comments putted in the attached document as follow:

**Reviewer #2:** General comments:

It would be much easier to read and review if you used the usual conventions for manuscript preparation e.g. the use subtitles/sections such as Introduction (which does not require a subtitle) Methods (with subsections Study Design and Context, Participants [including all details regarding sampling], Measures/Instruments (choose one and see note below about how to organize), Results (with subsection “Plan for Analysis” then report the actual analysis and results) and finally Discussion. Please see for example, the APA version 7 for how to prepare the document (although you would want to use numeric referencing as required by PLoSONE).

The following link may be helpful.

<https://www.apa.org/pubs/journals/resources/manuscript-submission-guidelines>

Using the conventional section for a manuscript will help to avoid redundancy or making reference to things not yet described, which occurs fairly frequently in the paper.

**Authors Response**: Thank you for your thoughtful suggestions. We amended our article in response to your comments and in the revised paper; we followed the standard manuscript writing conventions: introduction, methods and materials (research design and context, participants, measurement), results, discussion and conclusion (IMRDC).

**Reviewer #2:**  Please also note that indenting each new paragraph makes the manuscript much easier to read, especially to review.

**Author Response:** Thank you. We accepted your comments and modified them in the revised manuscript.

**Reviewer #2:**  Another main consideration here is the paper organization – this applies to all aspects of the paper (though much less so the introduction, which is sensibly written) but even more to the methods, results and discussion. The reporting of measures, results and discussion are far to piece-meal and list-like. It makes it very hard for the reader to digest or to grasp the big picture of the findings, let alone to make sense of them.

**Author Response:** We corrected the above comments in the updated article and tried to organize the methodology, results, and discussion section in accordance with the precise remarks comments provided for us.

**Reviewer #2:**  I understand this is primarily an exploratory paper (no apparent hypotheses are being tested) but suggest the authors consider 2-3 primary predictors and then perhaps others that are secondary. Report the results (and also address in the discussion) the primary predictors first looking at impact on all outcomes (depression, anxiety and somatic illness) simultaneously, not in different paragraphs. Then summarize these findings (in the discussion, discuss them). Then concisely report the picture for the secondary predictors, also looking at impact on all outcomes simultaneously, followed by a brief summary. Readers can consult the tables for the different outcomes if they want the specific findings for each predictor and outcome combination.

**Author Response:** We accept the provided suggestions and corrected them in the revised manuscript (example: line number 464-465).

**Reviewer #2:**  In other words, the results and discussion need to be better and more tightly organized. The authors need to consider what are the most important findings to share, to paint the big picture and how it fits into the rest of the literature, and then organize the paper to highlight these findings.

**Authors’ response:** your comment is accepted and we tried to refine the most important findings and tried to compare with the existed body of knowledge and literature (example: page 23 from the result part and page 29 from discussion part**).**

**Reviewer #2:**  **Specific recommendations:**

**Title:**

Depression, Anxiety, Somatic symptom and their determinants among High School

and Preparatory School Adolescents in Gondar Town, Northwest Ethiopia: Non-recursive

Structural Equation Modeling.

**Suggested Revised Title:**

Determinants of adolescent depression, anxiety, somatic symptoms in Northwest Ethiopia. **Authors’ response**: Thank you, we have corrected the title as per the recommendation (line number 1-3).

**Abstract**

**Reviewer #2:**  In general (throughout the paper), care should be taken to guard the anonymity and confidentiality of the participating sample. See e.g. the suggested revised title. In the abstract, 2^nd^paragraph “…to 1407 adolescents in northwest Ethiopia”.

**Authors’ response**: We took your excellent advice. We modified it in the revised manuscript (example: line 20).

**Reviewer #2:**  Please remove all names of particular schools and keep description to the region where the study was conducted, the two different types of schools and how they differ and keep sample demographics and so on in Participants section. Comment to remove the names of particular schools applies throughout the paper.

**Authors’ response:** thank you for your insightful suggestion**,** based on your advice, the names of the schools have been deleted from the entire version of the amended manuscript in order to maintain the confidentiality of the participants.

Results section in the abstract:

**Reviewer #2:**  It would be better to organize and to make more concise the results reported. Perhaps according to outcomes? Determinants of anxiety, then of depression, then interrelated findings for these variables.

**Authors’ response:** We appreciate the reviewer's suggestions to improve the quality of our article. The comment is accepted and modified (line number 25 to 30).

**Reviewer #2:**  **Introduction (Does not need to be labeled “Introduction”)**

**Authors’ response**: We appreciate your suggestion. The introduction is listed as one of the publication's components in the submission rules for the PLOSONE journal. We corrected it according to the journal’s manuscript preparation guideline (line 41).

**Reviewer #2:**  At line 68, please place a comma after “…adolescents, is characterized…”

**Authors’ response:** Thank you for the comment, we accepted the comment and corrected it in the revised manuscript (line number 58).

**Reviewer #2:**  Lines 70-71 Suggest move the sentence on the impact of depression combined with anxiety to the end of the next para on anxiety.

**Authors’ response:** Thank you for your comment; we have accepted and modified it (line number 80-81).

**Reviewer #2:**  Line 76, I think you mean “threats” and not “treats”

**Authors’ response**: Yes, it to mean threats, and we corrected it in the revised manuscript (line 71).

**Reviewer #2:**  Line 90 “..think or experience feelings about symptoms…”

**Authors’ response**: Thank you for the suggestion and we have corrected it in the revised manuscript (line number 88).

**Reviewer #2:**  Line 101-102, the sentence is not complete. Please attach sentences starting at 101 and 104 to the para above.

**Authors’ response:** We acknowledged the suggestion and we corrected it in the revised manuscript (line number 64, 81 and 95).

**Reviewer #2:**  Line 104-106 What is the nature of the relation? Positive or negative?

**Authors’ response**: Thank you for the comment. We have accepted and modified it (line 98).

**Reviewer #2:**  Line 113-114 Suggest, “Finally, individuals with… may experience physical harm from unnecessary medical procedures”

**Authors’ response:** thank you for your suggestion, we agreed with your suggestion and we corrected it in the revised manuscript (line number 94 -95).

**Reviewer #2:**  Line 135 Not clear here what you mean by “as an input toward sustainable development”.

**Authors’ response:** Thank you for your question, an input towards the sustainable development goal is to say; the slogan of the SDG is "no health without mental health," and in the SDG focusing on adolescents, mental health is the main target, because more than half of mental health disorders begin in adolescence and progress to adulthood. SDG agenda includes research as one of the initiatives to address the mental health problem. In light of this, our study will help to accomplish the Sustainable Development Goal. That is why you say it is a contribution to sustainable development.

**Methods (Include this major subtitle)**

**Reviewer #2:**  Line 138 Suggest “Study Design and Context” as first subsection.

**Authors’ response:** Thank you for the suggestion, we accepted your suggestion and it is modified in the revised manuscript (line number 123).

**Reviewer #2:**  Sample Description – Label as Participants

**Authors’ response:** Thank you for the suggestion, we accepted the suggestion and it is corrected (line number 135).

**Reviewer #2:**  Please justify the use of this particular population of Ethiopian adolescents. How might this contribute to or constrain generalization of study findings? Is this a convenience sample?

**Authors’ response:** Thank you for the comments and questions, based on your suggestion we included an idea related with the use of particular population of Ethiopian adolescents in the revised manuscript from line number 129 to 130 and the sampling is stratified random sampling rather than convenient sampling.

**Reviewer #2:**  Line 149, suggest remove the section on population as this information is given elsewhere.

**Authors’ response:** we accepted the suggestion and population section was omitted in the revised manuscript.

**Reviewer #2:**  Lines 162-169 please correct the use of parentheses, 1 set with commas between different types of free parameters). Currently, very hard to follow.

**Authors’ response:** thank you for the comment; we simplified it in the revised manuscript (line number 140-143).

**Reviewer #2:**  Line 171, if the sampling was stratified by the 2 different types of schools, say so here or above under context. If stratified first by type of school and then grade, at this point please include “(see below)” otherwise the comment here about a 2-stage sampling procedure seems out of place.

**Authors’ response:** Thank you for the suggestion, we accepted your idea and in the revised manuscript we have corrected it (line 162).

**Reviewer #2:**  Line 183, under sampling procedure, I would not name the particular schools involved in the study as this poses a risk to participant confidentiality. Knowing the names of particular schools does not enhance the study reporting.

**Authors’ response:** We acknowledge the comment of the reviewer, in the rewritten version as a whole; names of specific schools are omitted.

**Reviewer #2:**  Line 201, please place these variables in a table, and move them to supplementary text. Place a footnote or in the text refer the reader to the table for a summary of the key variables in the study.

### Authors’ response: We accepted the suggestion and modified it according to the feedback(line number 170).

**Reviewer #2:**  Line 216, suggest remove the section on “Operational definitions” and instead place the relevant information in the next section, immediately after you describe the measurement instruments. So after describing the depression measure very briefly, then say the criteria you used for categorization in the study.

**Authors’ response:** Thank you for the insightful suggestion, the section operational definition was omitted in the revised manuscript and information’s in the operational definition is incorporated in measurement section (page 10).

**Reviewer #2:**  Line 245 Suggested “self-reported questionnaire”.

**Authors’ response:** Your suggestion is acknowledged and we have corrected it (line number 173).

**Reviewer #2:**  Please be sure that in the sample description you indicate the percentage/proportion/number of sighted and sight challenged individuals and proportion by school type and gender perhaps? Sight capabilities of participants should not suddenly appear here.

**Authors’ response:** We thank the reviewer for the comment; we accepted and incorporated it (line number 173-174).

**Reviewer #2:**  Clarify if the questionnaires were administered in paper and pencil format or on a computer or other device.

**Authors’ response:** thank you for the comment, we accepted your comment; it was administered in pencil and paper format and it is included in the revised manuscript (line number 172).

**Reviewer #2:**  249-251, I do not understand the use of “The tool for this study here”. Do you mean “Questionnaires were designed to gather data on…” (insert your list of domains here).

**Authors’ response:** Thank you for the suggestion. yes it is to mean, questionnaire were designed to gather data on socio-demographic characteristics, behavioural factors......and we have corrected it in the revised manuscript (line number 176-178).

**Reviewer #2:**  Suggest place all of your measures in a table with columns for 1. Instrument name and citation 2.Brief description and 3. Criteria for categorization you included above.

**Authors’ response:** We accepted your suggestion and in the revised manuscript all of the measures are placed in the table (table 1, page 9-10).

**Reviewer #2:**  Line 268, there should be a subheading for results.

**Authors’ response:** Thank you for your feedback, we accepted the comment and corrected it in line number 181 of the revised manuscript.

**Reviewer #2:**  Line 271, does the translation apply to all of the questionnaires (were they arranged into one long document?)

**Authors’ response:** thank you for the question, yes translation was applied for the entire questionnaire after we arrange it in subheading.

**Reviewer #2:**  Although the Data Quality and Management efforts are laudable, this section is long and superfluous here.

**Authors’ response:** In the revised manuscript, after we put very essential parts in the main document, the remaining data quality assurance parts were moved to supplementary file (line 194).

**Reviewer #2:**  Suggest, remove lines 269 to first part of 271, you already mentioned trained data collectors above.

**Authors’ response:** We accepted the wise advice you provided, in the revised manuscript we have corrected it (line 182).

**Reviewer #2:**  Great that you checked face validity, construct validity and reliability. For the latter to please indicate very briefly how you checked these.

**Authors’ response**: Thank you for your suggestions to improve our manuscript, the construct validity was checked by using Average variance extracted and reliability was checked by using Cronbach’s alpha (line number 186-187 and 191-192).

**Reviewer #2:**  On second thought; perhaps you could have a “Measures” section, where you include the table suggested above. Before the table, include all information related to the measures such as translation and also efforts to get at validity and reliability. Include a footnote there and describe the pilot study briefly in the footnote. What was done? What did you find? Or place the pilot study in supplementary materials and refer to it in the paper.

**Authors’ response**: Thank you for the suggestion; based up on your idea the detailed part of the pilot study was placed in the supplementary material (S3 annex 1) (line 194).

**Reviewer #2:**  Note, it is not clear in what sense the pilot study constituted “participatory” research. Needs to be clarified wherever you put the pilot study description.

**Authors’ response**: Thank you, according to our search, there are two categories of pilot studies: participatory and non-participatory. So a participatory pilot study is a form pilot study in which participants are aware that they are taking part in a preliminary study and are invited to provide feedback to improve the questionnaire.

**Reviewer #2:**  Lines 279-286 Suggest remove the rest of this paragraph. What does it mean to say “After data entry, data recoding, and missing value management were considered accordingly.” If important, it could go at the start of the Results section, perhaps right before “Plan for Analysis”.

**Authors’ response:** Thank you, in the revised manuscript that unnecessary information’s from line number 279 to 286 are omitted**.**

**Reviewer #2:**  Are the Results given on page 14 for the pilot study? If yes, suggest the entire pilot study goes in Supplementary Material. Simply say in the manuscript that you did a pilot study to further investigate the validity of the questionnaire. Then refer the reader to Supplementary Material.

**Authors’ response:** We express gratitude for the reviewer's suggestions to improve our manuscript; yes it was for the pilot study and in the revised manuscript the detail part the pilot study was placed in the supplementary material (line number 194).

**Reviewer #2:**  The pilot study takes up a lot of space in the manuscript but, if I understand correctly, it is meant to convey background information to support the use of the questionnaire in the main study. The manuscript body should focus on the main study.

**Authors’ response:** Thank you for your suggestion, sure the manuscript body should focus on the main study and we tried to minimize pilot study part and we moved majority of pilot findings to supplementary file.

**Reviewer #2:**  Remove the sentence end of line 320 to 321. Connect the sentence at line 34 to the previous paragraph.

**Authors’ response:** Accepted and modified (line number 201).

**Results?**

**Reviewer #2:**  The results section is generally far too long, with too many explanatory details about how structural equation modeling works. If you aim to show your thoroughness in carefully undertaking this analysis, only mention the specific ways in which you did this in the paper. Assume the audience knows something about SEM.

**Authors’ response:** We acknowledged your recommendation, and in the updated manuscript, we attempted to minimize information about how the model works.

**Reviewer #2:**  Suggest re-ordering reporting of results to follow same order as mentioned in introduction and as the order of measures described in the methods section – depression, anxiety, somatic symptoms, then interrelated of these.

**Authors’ response:** Thank you for your suggestions to improve our manuscript, we accepted the suggestion and in the revised manuscript we follow the same pattern as the introduction; depression, anxiety and somatic symptom. (Example: see line 331, 337 and 343 for the prevalence of depression, anxiety and somatic symptom respectively).

**Reviewer #2:**  It is difficult the follow the list of findings in the paragraphs for each of these outcomes. Could you organize into direct effects only, unpack these, then indirect effects, unpack these and then perhaps deal with variables with both direct and indirect effects on the outcome that is the focus of that paragraph (depression, anxiety or somatic symptoms).

**Authors’ response:** Thank you, we accepted and modified it according to your feedback; (page 23-26).

**Reviewer #2:**  Line 677. “…Bangladesh use a cutoff above five…” please add “whereas in the current study, we used a cutoff on the X measure of Y”. Fill in X and Y accordingly.

**Authors’ response:** Thank you for the comment; we accepted it. However, based editors’ suggestion specific details about previous studies were not included in the revised manuscript.

Reviewer #2:  Interesting point. Please elaborate briefly on how socio-cultural factors might help to account for discrepancies in findings here.

**Authors’ response:** We acknowledge the reviewer comments to improve the quality of our manuscript, in the revised manuscript we tried to elaborate how socio cultural factors may account for the discrepancy in mental health illness (line number 455 to 461).

**Reviewer #2:**  Line 683, It could also be that females are more likely to report on their depression!

Cultures vary in terms of how males and females ought to deal with things such as depression and this might have an impact on how willing or unwilling males and females are report being depressed or having other mental health challenges.

**Authors’ response:** Thank you for the suggestion, we accepted your suggestion and it is included in the revised manuscript (line number 473-476).

**Reviewer #2:**  Line 689, regarding the following sentence:

“Besides, adolescent girls are more likely to have negative life events in relation to their parents and peers, and females are more emotion focused and have a distracting coping style than males”

Please unpack these findings so the reviewer can understand exactly what the authors of the cited paper found. It is not clear to me why adolescent girls are more likely to have negative life events in relation to their parents and peers. What does it mean to be “more emotion focused and to have a distracting coping style than males”?

**Authors’ response:** We respect your thoughtful observation, after we dig out different source, there is no tangible evidence which help us to elaborate this idea and in the revised manuscript the justification adolescent girls are more likely to have negative life events in relation to their parents and peers, and females are more emotion focused and have a distracting coping style than males are omitted

**Reviewer #2:**  Line 699 – 700, these claims need supporting references.

**Authors’ response**: Accepted and cited (line number 513).

**Reviewer #2:**  Line 701, it is also possible that people who are more depressed are more likely to use alcohol as a coping mechanism.

**Authors’ response:** We appreciate your suggestion and agree with your worry. People who are depressed are more inclined to use alcohol as a coping strategy. It is difficult to tell whether alcohol usage or depression develops first, and this is one of our study's shortcomings (line number 546-547).

**Reviewer #2:**  Line 707. It could also be that being depressed means you have low motivation to study or even that you do not see any point in striving in school given low or no expectations regarding a well-adjusted, productive or successful future.

**Authors’ response:** Thank you for your thoughtful comment. As with the link between drinking and depression, bad academic performance may be caused by depression, and the two conditions may be correlated in both directions. However, because of the cross-sectional nature of the data, we are unable to determine which comes first in our study and you failed to consider how depression affects academic performance. We included it to the study's limitations in order to concern future researchers (line number 547-547).

**Reviewer #2:**  In reporting results, it would be group according to outcome e.g. depression. Then review of the variables with a direct effect, briefly unpack, those with indirect effects, briefly unpack and then with both direct and indirect impact, then describe – in other words follow the same list and organization suggested for reporting results.

**Authors’ response:** We accepted the wise advice you provided, and in the revised manuscript we unpack the direct effect first, then indirect effect and finally both direct and indirect effect were unpacked (page 23-26).

**Reviewer #2:**  Losing a loved one is a traumatic and by definition a sad experience. This is not a surprising finding and does not seem to require much more unpacking. Respondents could still be recovering from this loss.

**Authors’ response:** Even though the depressive symptoms persist in some individuals with a history of death loved one, majority of the individuals may recover from it by themselves. So we accepted your suggestion and the justification related with death of loved one is omitted in the revised manuscript.

**Reviewer #2:**  Line 743 and 744, interesting finding re: private school and higher rates of depression but in the results you report both direct (negative) and indirect (positive) with a small net positive effect. It’s worth referring to this and elaborating briefly here.

**Authors’ response:** We respect your thoughtful observation, being in private increase the likelihood of depression both directly and indirectly with a small net positive effect. Even though the net effect is little, it is still important; although it is important to prioritize and elaborate on aspects that have a greater impact, we still believe that it is important to examine or explore small but important significant effects.

**Reviewer #2:**  Line 777-778, if you are going to suggest that cultural differences might account for inconsistencies in results across studies, good to give an example of exactly how this might play out.

**Authors’ response:** We appreciate the reviewer's efforts to enhance the quality of our research; the updated manuscript describes the role of cultural variation to the inconsistencies (line number 455-461 and 474 -476).

**Reviewer #2:**  Lines 786-788, “…students with high self-rated academic ability will be more

likely to make decisions, express their feelings without feeling embarrassed” Doing better academically is likely to increase confidence compared to doing poorly and these might lead to more confidence in decision making but it is not clear to me why this would increase the willingness to express feelings without being embarrassed and necessarily to more optimism.

**Authors’ response:** We appreciate your query and your agreement that adolescents who excel academically will be more valued by their family, friends, and teachers, which will help them gain the confidence to communicate their feelings. It is essential for young people's successful social development. Students who do well in school have a lower risk of suicide, which makes them more prepared to transition into adulthood. Additionally, adolescent who have excelled academically will say, "I can do anything," and those around them will support and encourage them with resources and ideas, which will improve their decision-making abilities.

**Reviewer #2:**  Lines 799-805, again it could be that women are more likely to disclose struggles with anxiety compared to men.

**Authors’ response:** Accepted and incorporated (line number 473-476).

**Reviewer #2:**  The point above about organizing study findings in the results and discussion applies to all of the outcomes. Another way to organize might be to treat each predictor in turn and then consider the impact on all three outcomes. There were some common findings for predictors with different outcomes e.g. alcohol consumption and private school both had a positive relationship with depression and also with anxiety. The explanations for the impact on different outcomes might be the same, so would only need to be mentioned once.

**Authors’ response:** We took your wise advice into account. As we already indicated for previous comment, we attempted to break down the direct effect first, followed by the indirect effect, and then both the direct and indirect effects in the result part. In the discussion section, we first talk about the factors that affected all of the outcomes, and then we talk about the factors that had a particular impact on one of the outcome (page 23-25 for result part and page 27-31 for discussion part).

**Reviewer #2:**  Study Strengths and Limitations

Generalizability is also constrained by the specific, targeted sample. The authors should recognize and address this point. Is the sample nonetheless diverse and representative of Ethiopian adolescents and if yes, how do you know this and what then does this mean for study generalizability but also for policy and practice in Education, in Ethiopian society?

**Authors’ response:** Your thoughtful suggestion was accepted, and it has been incorporated in to the limitation part (line number 543 to 544).

**Reviewer #2:**  In several places in the discussion, the authors raise the point of cultural differences and how these might account for discrepancies in study findings across studies conducted in different cultures. But they never really expand on the nature of those differences. Yet this would be so interesting here and contribute to the literature on factors that contribute to mental health outcomes and their context specificity or universality, which has knock on effects for how to mitigate these struggles or how to approach treatment.

**Authors’ response:** Acknowledged, and we provide a justification (line number 455-461 and 474 -476).

**Reviewer #2:**  It would be better if the recommendations were not stated in bullet form.

**Authors’ response**: Thank you, we have accepted and modified it in the revised manuscript (line number 555 -562).

Line 987 and on

**Reviewer #2:**  To avoid the possibility of stigma surrounding coping with and addressing mental health issues, preventative and coping strategies at every level might be better if offered to everyone, not targeting those with certain characteristics highlighted by the study findings. Targeted treatment might invoke stigma which could actually negatively impact mental health.

**Authors’ response:** Thank you accepted and updated in the revised manuscript (line number 556 to 557).

**Reviewer #2:**  Line 991, see suggestion above

**Authors’ response**: Accepted and modified (line number 557-558).

**Reviewer #2:**  Line 999, I do not think you established that drinking alcohol caused mental health issues. If this is a coping mechanism or addiction is in play, it might be better to seek treatment for the mental health issues as alcohol consumption is likely to come up as part of this. Parental sudden restriction but exacerbates symptoms without expert treatment.

**Authors’ response:** Thank you for your comments. Due to the cross-sectional nature of the data, we did not establish a causal relationship between alcohol intake and mental illness and we have putted it in the limitation (line number 547-548).

**Reviewer #2:**  Line 1001, again alcohol consumption might be a coping mechanism or indeed a symptom of struggles rather than causally related. Not as well that you only asked about “ever consumption” not about how much or how frequently or for how many years consumed (another study limitation).

**Authors’ response:** Accepted, and we incorporate it in the limitation (line number 546-547).

**Reviewer #2:**  Line 1006, interesting suggestion regarding out-of-school adolescents. It would be good to provide prevalence data – these might be indicators of struggles with mental health in themselves.

**Authors’ response:** Thank you for your suggestion; since we did not collect data on out-of-school adolescents, we are unable to give prevalence information at this time. However, we will conduct another study on them in the future.

Thanks again

Yours sincerely,

On behalf of the authors,

**Zenebe Abebe**
