# Supplementary material for: Determinants of adolescents’ depression, anxiety, and somatic symptoms in Northwest Ethiopia: A non-recursive structural equation modeling
Source: PLoS One. 2024 Apr 10;19(4):e0281571. doi: 10.1371/journal.pone.0281571 (PMC11006201; doi:10.1371/journal.pone.0281571)
Supplement: Attachment — Submitted filename: Response to reviewers.docx [file pone.0281571.s017.docx]

**Response to Editor and Reviewers**

1. **Response to chief academic editor**

**Manuscript title:** Determinants of adolescent’s depression, anxiety, and somatic symptoms in Northwest Ethiopia: A Non-recursive structural equation modeling.

**Manuscript ID:** PONE-D-23-02244R2

**Authors response**: Dear Editors and Reviewers, Thank you for giving us the opportunity to submit a revised version of the manuscript “Determinants of adolescent’s depression, anxiety, and somatic symptoms in Northwest Ethiopia: A Non-recursive structural equation modeling.” for publication in the PLOS ONE journal. We appreciate the time and effort you and the reviewers put into providing feedback on our article, and we appreciate the insightful remarks and useful advice you gave, which considerably improved the manuscript. We have taken into account the reviewers' suggestions. Track modifications are used to highlight these changes in the revised document.

Journal Requirements:

Please review your reference list to ensure that it is complete and correct. If you have cited papers that have been retracted, please include the rationale for doing so in the manuscript text, or remove these references and replace them with relevant current references. Any changes to the reference list should be mentioned in the rebuttal letter that accompanies your revised manuscript. If you need to cite a retracted article, indicate the article’s retracted status in the References list and also include a citation and full reference for the retraction notice.
**Authors’ response:** The editor's use of full insight to check for the inclusion of retracted paper in our reference list is really appreciated. We reviewed our references, and there is no reference list of works that have been retracted in our manuscript.
**Additional Editor Comments (if provided):**

**Editor**: Please address reviewer's comment as advised.

**Authors:** Dear Chief Academic Editor, Thank you for assisting us in emphasizing the reviewer's comment. We attempted to answer the reviewer's comments one by one.

**Reviewers' comments:**
Reviewer's Responses to Questions

**Comments to the Author**
1. If the authors have adequately addressed your comments raised in a previous round of review and you feel that this manuscript is now acceptable for publication, you may indicate that here to bypass the “Comments to the Author” section, enter your conflict of interest statement in the “Confidential to Editor” section, and submit your "Accept" recommendation.

Reviewer #1: All comments have been addressed

Reviewer #2: All comments have been addressed

2. Is the manuscript technically sound, and do the data support the conclusions?
The manuscript must describe a technically sound piece of scientific research with data that supports the conclusions. Experiments must have been conducted rigorously, with appropriate controls, replication, and sample sizes. The conclusions must be drawn appropriately based on the data presented.

Reviewer #1: Yes

Reviewer #2: Yes

3. Has the statistical analysis been performed appropriately and rigorously?

Reviewer #1: Yes

Reviewer #2: Yes

4. Have the authors made all data underlying the findings in their manuscript fully available?

The [PLOS Data policy](http://www.plosone.org/static/policies.action#sharing) requires authors to make all data underlying the findings described in their manuscript fully available without restriction, with rare exception (please refer to the Data Availability Statement in the manuscript PDF file). The data should be provided as part of the manuscript or its supporting information, or deposited to a public repository. For example, in addition to summary statistics, the data points behind means, medians and variance measures should be available. If there are restrictions on publicly sharing data—e.g. participant privacy or use of data from a third party—those must be specified.

Reviewer #1: Yes

Reviewer #2: Yes

5. Is the manuscript presented in an intelligible fashion and written in Standard English?
PLOS ONE does not copyedit accepted manuscripts, so the language in submitted articles must be clear, correct, and unambiguous. Any typographical or grammatical errors should be corrected at revision, so please note any specific errors here.

Reviewer #1: Yes

Reviewer #2: No

6. Review Comments to the Author

Please use the space provided to explain your answers to the questions above. You may also include additional comments for the author, including concerns about dual publication, research ethics, or publication ethics. (Please upload your review as an attachment if it exceeds 20,000 characters)

Reviewer #1: (No Response)

Reviewer #2: In general the paper is greatly strengthened and I appreciate the close attention to the reviewers' comments and the changes made to the manuscript. My main remaining comments are .

2. ***Response to reviewer 2***

**Author responses**: Dear Dr. Tracy Solomon, first and foremost, we would want to express our gratitude for your tremendous effort, dedication, humility, and time spent commenting on our paper. Following that, we'd like to respond to the comments point by point.

***Reviewer #2***: 1. As another reviewer/editor has pointed out, the occurrence of anxiety, depression and somatic symptoms in adolescence is not a new hypothesis or finding. The unique contribution of the paper is documenting these issues in an Ethiopian adolescents and the exploration of potential contributing factors in that population. Hence, I would start the paper by making this point before moving on to define anxiety, depression (I would use this order) and somatic disorders, their prevalence in other regions, other parts of Africa and then Ethiopia, perhaps pointing out the dearth of research on Ethiopian adolescents in the conclusion of this section of the introduction.

**Authors’ response:** Dear Dr. Tracy Solomon, we are grateful for pointing out this. The paper's distinctive contribution is not only that it documents the issue in Ethiopian teenagers, but it also addresses the indirect effect of predictors, and we attempted to incorporate what distinguishes adolescents in northwest Ethiopia.Kindly look at line number 108-114 in the revised manuscript without track changes. We prefer the sequence of depression, anxiety, and somatic symptoms (DASS) in terms of severity.

**Reviewer #2.** Efforts at writing in English which may not be the native language of the authors is always admirable, in my view, and should not prohibit the publishing of important and interesting data as we have here. However, for maximum impact, it would make sense for the authors to have a reviewer read the paper for smooth flow in the English language as a final polish before submitting. In general, the paper is clear but this is scientific writing and precision in language counts. No criticism of the authors, just a suggestion to "professionalize" the writing further before being considered for acceptance. I wish the authors the best of luck in getting the paper accepted for publication.

**Authors’ response:** Dear Dr. Tracy Solomon, thank you for pointing out this issue. All authors evaluated the text several times; senior writers were consulted, and internet tools such as Grammarly (<https://app.grammarly.com/ddocs/2167802892>) were used in the updated manuscript to make the paper clearer.

Thanks again

On behalf of the authors,

**Zenebe Abebe**
