# Supplementary material for: Determinants of adolescents’ depression, anxiety, and somatic symptoms in Northwest Ethiopia: A non-recursive structural equation modeling
Source: PLoS One. 2024 Apr 10;19(4):e0281571. doi: 10.1371/journal.pone.0281571 (PMC11006201; doi:10.1371/journal.pone.0281571)
Supplement: Attachment — Submitted filename: Response to reviewers.docx [file pone.0281571.s019.docx]

**Response to Editor and Reviewer**

**Manuscript title:** Determinants of adolescent’s depression, anxiety, and somatic symptoms in Northwest Ethiopia: A Non-recursive structural equation modeling.

**Manuscript ID:** PONE-D-23-02244R3

**Author’s response**

Dear Editors and Reviewers,

We would like to express our gratitude for allowing us to submit a revised version of our manuscript titled "Determinants of adolescent's depression, anxiety, and somatic symptoms in Northwest Ethiopia: A Non-recursive structural equation modeling" for publication in the PLOS ONE journal. We sincerely appreciate the time and dedication that both you and the reviewers have invested in providing feedback on our article. We are truly grateful for the insightful remarks and valuable advice that have significantly enhanced the manuscript. We have carefully considered the suggestions put forth by the reviewers, and the revised document incorporates these changes. To highlight these modifications, we have utilized track modifications in the revised version. Thank you once again for your invaluable support and guidance throughout this process.

Journal Requirements:

Please review your reference list to ensure that it is complete and correct. If you have cited papers that have been retracted, please include the rationale for doing so in the manuscript text, or remove these references and replace them with relevant current references. Any changes to the reference list should be mentioned in the rebuttal letter that accompanies your revised manuscript. If you need to cite a retracted article, indicate the article’s retracted status in the References list and also include a citation and full reference for the retraction notice.
**Authors’ response:** We are grateful for the editor's thorough review, specifically in checking for the presence of retracted papers in our reference list. After carefully reviewing our references, we can confirm that our manuscript does not include any works that have been retracted. We appreciate the editor's attention to detail in ensuring the accuracy and integrity of our reference list.

**Comments to the Author**

1. If the authors have adequately addressed your comments raised in a previous round of review and you feel that this manuscript is now acceptable for publication, you may indicate that here to bypass the “Comments to the Author” section, enter your conflict of interest statement in the “Confidential to Editor” section, and submit your "Accept" recommendation.

Reviewer #1: All comments have been addressed

Reviewer #2: (No Response)

2. Is the manuscript technically sound, and do the data support the conclusions?

The manuscript must describe a technically sound piece of scientific research with data that supports the conclusions. Experiments must have been conducted rigorously, with appropriate controls, replication, and sample sizes. The conclusions must be drawn appropriately based on the data presented.

Reviewer #1: Yes

Reviewer #2: Yes

3. Has the statistical analysis been performed appropriately and rigorously?

Reviewer #1: Yes

Reviewer #2: Yes

4. Have the authors made all data underlying the findings in their manuscript fully available?

The [PLOS Data policy](http://www.plosone.org/static/policies.action#sharing) requires authors to make all data underlying the findings described in their manuscript fully available without restriction, with rare exception (please refer to the Data Availability Statement in the manuscript PDF file). The data should be provided as part of the manuscript or its supporting information, or deposited to a public repository. For example, in addition to summary statistics, the data points behind means, medians and variance measures should be available. If there are restrictions on publicly sharing data—e.g. participant privacy or use of data from a third party—those must be specified.

Reviewer #1: Yes

Reviewer #2: (No Response)

5. Is the manuscript presented in an intelligible fashion and written in standard English?

PLOS ONE does not copyedit accepted manuscripts, so the language in submitted articles must be clear, correct, and unambiguous. Any typographical or grammatical errors should be corrected at revision, so please note any specific errors here.

Reviewer #1: Yes

Reviewer #2: Yes

6. Review Comments to the Author

Please use the space provided to explain your answers to the questions above. You may also include additional comments for the author, including concerns about dual publication, research ethics, or publication ethics. (Please upload your review as an attachment if it exceeds 20,000 characters)

Reviewer #1: (No Response)

***Reviewer 2***

Reviewer #2: This paper is so very much improved from my previous reading of it. The study is well executed, the writing so much improved and the analysis thorough and responsibly reported. I have made suggestions for further tightening of writing only - I sincerely hope to be helpful here not to delay the manuscript processing further. My comments have been made on the manuscript PDF directly. Please see the attachment that bears my initials TS at the end. If the authors attend to these minor suggestions the paper will be in fine shape for publishing in my view.

**Authors’ response:** Dear Dr. Tracy Solomon, First and foremost, we would like to extend our sincere gratitude for your exceptional effort, dedication, humility, and the significant amount of time you devoted to reviewing and providing comments on our paper. We highly value your feedback, and we would like to address each comment point by point as follow.

1. **ABSTRACT**

***Reviewer 2:*** *line number 25% missing*

***Author response:*** Thank you for your suggestion. We have made the necessary corrections in the revised manuscript (Kindly look at line number 25).

***Reviewer 2:*** *Line number 27: Ever use of alcohol, change it to alcohol use*

***Author response:*** *We appreciate your insightful suggestion, and we have addressed it in the revised manuscript. We kindly request you to review line number 27 for the specific changes made.*

2. ***INTRODUCTION***

***Reviewer 2: line number: which is associated with increased …?***

***Authors’ Response:*** Thank you for your valuable comments. We have incorporated them into the revised manuscript, and we kindly request you to review the end of line 42 to 43 for the specific changes made.

***Reviewer 2: involving significant***

***Authors’ Response:*** We greatly appreciate your valuable input. Taking your feedback into consideration, we have made the necessary corrections in the revised manuscript (kindly look at line number 44 in the revised manuscript).

***Reviewer 2:*** *Line number 46: This has been linked to…*

***Author response:*** Accepted and modified (kindly looks at line 45 in the revised manuscript).

***Reviewer 2:*** *Line number 56: add most*

***Author response:*** *Thank you for your feedback. We have accepted your suggestions and made the appropriate modifications in the revised manuscript (kindly looks at line number 55 )* ***Reviewer 2: line 58:*** *As mentioned in the earlier review, why not start with the most common anxiety and then move to the second common depression. Odd start to this paragraph if the next one is about anxiety*

***Author response:*** Thank you for providing your comments once again. We have carefully considered your suggestions and made the necessary amendments in the revised manuscript. We kindly request you to review lines 57-83 for the specific changes implemented.

***Reviewer 2: line number 96:*** *Female sex: female*

***Author response:*** Accepted and modified (kindly looks at line 95 in the revised manuscript).

***Reviewer 2: line number 97:*** *Extra school change to extra curricular*

***Author response:*** Thank you for providing your feedback. We have carefully corrected it in the revised manuscript (line number 95).

***Reviewer 2:*** *Line 99: remove although and add yet after the comma*

***Author response:*** *thank you, accepted and modified (kindly looks at line number 98-99 in the revised manuscript)*

***Reviewer 2:*** *Line 103: although adolescents*

***Author response:*** thank you for your feedback; we have corrected it according to your suggestion (kindly looks at line number 102).

***Reviewer 2:*** *Line 108: replace moreover..And make it the third sentence*

***Author response:*** We greatly appreciate your suggestion. It has been accepted, and we have made the necessary modifications in the revised manuscript accordingly (kindly look line in the 108).

***Reviewer 2:*** Adolescents in northwest Ethiopia are especially vulnerable to mental illness, due to repeated internal conflicts in northern Ethiopia: ***Suggest making this your second sentence in paragraph.***

***Author response:*** Thank you for your input. We have accepted your suggestions and made the appropriate modifications (line 107-108).

***Reviewer 2:*** *Line 116: Therefore, this study will assess the prevalence and determinants of … and the relationship between these variables.*

***Author response:*** accepted and corrected (line 115).

3. ***METHODS AND MATERIALS***

Participants:

***Reviewer 2:*** line 141suggests gives total sample size here, put information regarding sample size calculation in footnote or in supplementary material. Then give description of the sample, perhaps in summary table. Proportion private and public schools, identified as male and female, by grade include average age with range or sd, any other demographic info if available such as SES, sighted vs blind individuals

***Author response:*** *Thank you for providing your invaluable comments. We have accepted and incorporated your suggestions into the revised version (kindly looks at page 7-9).*

***Reviewer 2:*** Line 159: this paragraph fine to include in the sample

***Author response:*** *thank you, we have accepted your feedback and made the necessary modifications (line number 140-141).*

***Reviewer 2:*** Line 169: I don’t know that you need both figure and textual description here.

***Author response:*** Thank you for your suggestion and feedback. As per your advice, we have decided to omit the textual description (kindly looks at line 141) from the revised version***.***

Data collection procedures and tools

***Reviewer 2:*** Line 178: did participants complete the questionnaire in group setting or were they assessed individually. Where were they assessed? In the school library? Classroom? Quite area in the school? How long was the test session? Were breaks taken as needed?

***Author response:*** Thank you for your feedback, which allowed us to incorporate this information. In the revised manuscript, we have included the details that the participants completed the questionnaire in classroom setting, and the completion time varied between 15 and 25 minutes (kindly look line 175-176).

***Reviewer 2:*** Page 10, table 1: ever use of alcohol: please change to alcohol use?

***Author response:*** *We appreciate your suggestion, and we have taken it into account in the revised manuscript. The term "alcohol use" has been included consistently throughout the entire document, as you recommended.*

*Data quality assurance*

***Reviewer 2:*** Line 185: suggest place this section in supplementary material

***Author response:*** Thank you for your input. In the revised manuscript, we have moved this section to the supplementary material as per your suggestion (kindly look at line 187).

*Ethical consideration*

***Reviewer 2:*** Line 311: as judged by …? State if they were excluded from the study. What was the resulting effective sample?

***Author response:*** Dear Dr. Tracy Solomon, We would like to express our gratitude for bringing this issue to our attention. In our study, non-volunteer individuals were indeed excluded (line *number 314-315 in the revised manuscript).* However, we did not exclude individuals with moderate to severe depression, anxiety, and somatic symptoms, as their status became known only after they completed the questionnaire.

4. **RESULTS**

***Reviewer 2:*** Line number 318: suggest place all of this section under participants, at the start of method section.

***Author response:*** Thank you for your suggestion. We have taken it into consideration and made the necessary modifications accordingly (page 7-9).

***Reviewer 2:*** Line 327: I would also include under sample description-text only. Move the table here to supplementary materials

***Author response:*** Thank you for your suggestion. We have accepted it and made the necessary modifications in the revised manuscript. Kindly refer to lines 150-155 in the revised version for the specific changes implemented.

***Reviewer 2:*** Line 336: suggest for this section:

“Results “The subtitle:”plan for analysis” then subtitles for each of your study goal “prevalence” “contributing factors “you could combine the last 2 sub-sections. If you will speak to them simultaneously.

***Author response:*** Dear Dr. Tracy Solomon, Thank you for your suggestion. We appreciate your recommendation and acknowledge that including the plan of analysis in the result section is one of the possible options. However, in health science writing, it is also common to present the plan for analysis in the method section, which is the approach we have chosen to follow in this study. In the previous manuscript, we had already incorporated the plan for analysis under the section titled "Data Processing, Model Building, and Analysis" (line 188). Therefore, we did not specifically include the plan of analysis in the result section. Instead, we opted to start the result section with the title "Results," followed by subsections that cover the prevalence of depression, anxiety, somatic symptoms, and contributing factors.

***Reviewer 2:*** Line 356: best under plan for analysis. Condense this section as much as possible, describe in sufficient detail for the reader to understand what you did but micro stuff in supplementary material section. Such a good paper, so thoroughly in approach but this gets lost in overwhelming the reader with all of details in the main manuscript. You don’t have to scarify by cutting all your work but consider moving to supplementary material.

***Author response:*** We appreciate your suggestion, and as per your recommendation, we have incorporated this section into the subsection titled "Data Processing, Model Building, and Analysis." Thank you for your valuable input, which has helped us improve the organization of the manuscript (kindly look line 289-295).

**DISCUSSION**

***Reviewer 2:*** Line number 439: we were also interested in the inter-relation between these factors

***Author response:*** accepted and modified (kindly look line 410 in the revised manuscript).

***Reviewer 2:*** Line 440: “moderate” leave details to the next paragraph

***Author response:*** Thank you for your valuable comment, which has greatly contributed to strengthening our paper. We have carefully considered your feedback and made the necessary modifications accordingly (line 411).

***Reviewer 2:*** Line 460: do you mean only somatic disorders here or disparity for depression, anxiety to?

***Author response:*** Thank you for bringing this issue to our attention. We intended to highlight the variation in depression, anxiety, and somatic symptoms, and we have now incorporated this information into the revised document (line 430).

***Reviewer 2:*** Line 468: ongoing internal conflict

***Author response:*** *and made the necessary modifications in the revised manuscript. Kindly refer to line 439 in the revised version to see the specific changes that have been implemented.*

***Reviewer 2:*** Line 470: suggest you discuss findings with respect to predictors as follow:

1. Higher incidence in female and why?
2. Group academic ability, private school and perceived social support together. For example have an opening statement that all of these had an impact on all 3 of your outcomes , then unpack for each of these, the stress of being in private school with high academic ability….

**Authors’ response**: Dear Tracy Solomon, We would like to express our sincere appreciation for your valuable efforts in improving our paper. Your feedback has been invaluable, and we have accepted your comment and made the necessary modifications accordingly (kindly looks at page 27 -29).

***Reviewer 2:*** line 483: good here to also mention gender roles, if there are greater disparities in male and female expectation, and these do not favor female, they would have more reason to experience depression, anxiety and possibly somatic disorders.

***Author response:*** Thank you for bringing this issue to our attention. We have accepted your feedback and incorporated the necessary changes in the revised manuscript. Please refer to lines 483-486 in the revised version to review the specific modifications that have been made.

***Reviewer 2:*** Line 484: Please use "alcohol use" throughout your paper

***Author response:*** Thank you for your comment. We have taken your suggestion into account and used the term "alcohol use" consistently throughout the entire revised manuscript.

***Reviewer 2:*** Line 494: could alcohol be the symptom of these disorders?

***Author response:*** Thank you for guiding us in this direction. We acknowledge that your suggestion could indeed be beneficial, and we have included it in the revised manuscript (kindly loot line 537-538).

***Reviewer 2:*** Line 539: subtitle here for relation between variables. Suggest place after the paragraph on prevalence and before the long section predictors.

***Author response:*** Thank you for your suggestion. We have accepted your suggestion and implemented the required changes accordingly. Please refer to line 445 in the revised manuscript.

5. **STRENGTHS AND LIMITATIONS**

***Reviewer 2:*** Line 551: public and private schools included and data on under-represented sample in the broader literature are the strengths.

***Author response****: We have taken your recommendation into account and integrated it into our work (kindly look line 551-552).*

***Reviewer 2:*** Line 554: question says “in the last 3 months”

***Author response:*** We have embraced your suggestion and implemented the required edits accordingly (kindly looks at line 556).

*END!*

Dear Dr Tracy Solomon: We value your feedback and have taken it into careful consideration during the revision process. We believe that your suggestions have significantly enhanced the quality and clarity of our paper. Once again, we sincerely thank you for your invaluable contribution.

Best regards,

Zenebe Abebe (corresponding author)
